# Bayesian genome scale modelling identifies thermal determinants of yeast metabolism

**DOI:** 10.1038/s41467-020-20338-2

**Published:** 2021-01-08

**Authors:** Gang Li, Yating Hu, Jan Zrimec, Hao Luo, Hao Wang, Aleksej Zelezniak, Boyang Ji, Jens Nielsen

**Affiliations:** 1 Department of Biology and Biological Engineering, Chalmers University of Technology, SE-412 96 Gothenburg, Sweden; 2 National Bioinformatics Infrastructure Sweden, Science for Life Laboratory, Chalmers University of Technology, SE-41258 Gothenburg, Sweden; 3 Wallenberg Center for Molecular and Translational Medicine, University of Gothenburg, SE-41258 Gothenburg, Sweden; 4 Science for Life Laboratory, Tomtebodavägen 23a, SE-171 65 Stockholm, Sweden; 5 Novo Nordisk Foundation Center for Biosustainability, Technical University of Denmark, DK-2800 Kgs. Lyngby, Denmark; 6 BioInnovation Institute, Ole Måløes Vej 3, DK2200 Copenhagen N, Denmark

**Keywords:** Computational models, Machine learning, Bayesian inference

## Abstract

The molecular basis of how temperature affects cell metabolism has been a long-standing question in biology, where the main obstacles are the lack of high-quality data and methods to associate temperature effects on the function of individual proteins as well as to combine them at a systems level. Here we develop and apply a Bayesian modeling approach to resolve the temperature effects in genome scale metabolic models (GEM). The approach minimizes uncertainties in enzymatic thermal parameters and greatly improves the predictive strength of the GEMs. The resulting temperature constrained yeast GEM uncovers enzymes that limit growth at superoptimal temperatures, and squalene epoxidase (ERG1) is predicted to be the most rate limiting. By replacing this single key enzyme with an ortholog from a thermotolerant yeast strain, we obtain a thermotolerant strain that outgrows the wild type, demonstrating the critical role of sterol metabolism in yeast thermosensitivity. Therefore, apart from identifying thermal determinants of cell metabolism and enabling the design of thermotolerant strains, our Bayesian GEM approach facilitates modelling of complex biological systems in the absence of high-quality data and therefore shows promise for becoming a standard tool for genome scale modeling.

## Abstract (web summary)

While temperature impacts the function of all cellular components, it’s hard to rule out how the temperature dependence of cell phenotypes emerged from the dependence of individual components. Here, the authors develop a Bayesian genome scale modelling approach to identify thermal determinants of yeast metabolism.

## Introduction

Temperature is a key environmental and evolutionary factor that shapes the physiology of living cells. Organisms have successfully adapted to survive in diverse temperature ranges^1–3^, where minor deviations from the optimal temperature by merely a few degrees can dramatically impair cell growth. For instance, the model eukaryotic organism *Saccharomyces cerevisiae* has an optimal growth temperature (OGT) of ~30 °C, whereas a temperature of 42 °C is already lethal to the organism^4,5^. Since cell growth fundamentally requires all cellular components to be functional in the temperature window of cell growth, proteins, an abundant group of biomolecules that carry out the majority of catalytic functions and are also highly sensitive to changes in temperature^5–7^, are considered to have a critical effect on cell physiology in relation to temperature. However, despite all our knowledge of temperature effects at both the cellular and molecular levels, including recent breakthroughs in temperature-dependent protein folding^7–10^ and enzyme kinetics^11,12^, the temperature association between proteins and cell physiology is still poorly understood.

Multiple studies have attempted to model the temperature effects on cell growth, though with very few proteome-wide parameters^13^. Examples include the dominant activation barrier and the number of essential proteins to cell growth^14^ and the activation energy of the growth process and the free energy change of protein denaturation^15^. These models showed excellent performance when describing the general cell growth rate at various temperatures, however, they could not pinpoint the specific rate-limiting enzymes, nor predict the amount of improvement in growth rate by replacing these enzymes with temperature-insensitive homologs.

To this end, genome-scale metabolic models (GEMs)^16–18^, which are a comprehensive mathematical representation of cellular biochemical reactions^19^, have been used to model the thermosensitivity of metabolism in *Escherichia coli*, for instance by associating metabolic reactions with protein structures^20^ or by modeling protein-folding networks^21^. It however remains challenging to model more complex, eukaryotic organisms, such as *S. cerevisiae*, due to their metabolic complexity^16^ as well as due to the lack of availability of the required enzymatic data^7,22^, including high-quality protein structures^20,21^. In addition, such GEMs rely on thousands of parameters to describe the temperature effects on protein folding and kinetics^16^, which have to be empirically or computationally estimated^20,21^. This leads to large statistical uncertainties in model parameters and can make the models unreliable, due to inaccurate temperature associations between proteins and cell physiology. Therefore, in order to enable accurate modeling of the temperature dependence of cell metabolism, a key requirement is to develop a modeling approach that resolves the issues with large uncertainties of temperature-related parameters and produces accurate temperature constrained predictions.

Hence, in the present study, we introduce a Bayesian genome-scale modeling approach to model the temperature effects on cellular metabolism in *S. cerevisiae*, the most widely used industrial organism with readily available thermal experimental data^5,23,24^ and highly sophisticated GEMs^16,18,25^. We first quantify and reduce the large uncertainties in the parameters describing enzyme thermosensitivity using Bayesian statistical learning^26^ to simulate phenotypic data. We show that the resulting models are capable of reproducing various experimental datasets and provide explicit insight into how yeast metabolism is affected by temperature. Our approach identifies the sterol metabolism as a key factor in the yeast thermal adaptation and predicts the flux-controlling enzymes in superoptimal temperature ranges as potential targets for the future design of thermotolerant yeast strains. We then experimentally validate the predicted most rate-limiting enzyme by replacing it with an ortholog from a known thermotolerant yeast *Kluyveromyces marxianus*. We hereby demonstrate the power of Bayesian genome-scale modeling for studying complex biological systems.

## Results

### Using Bayesian statistical learning to integrate temperature dependence in ecGEMs

**Fig. 1:**
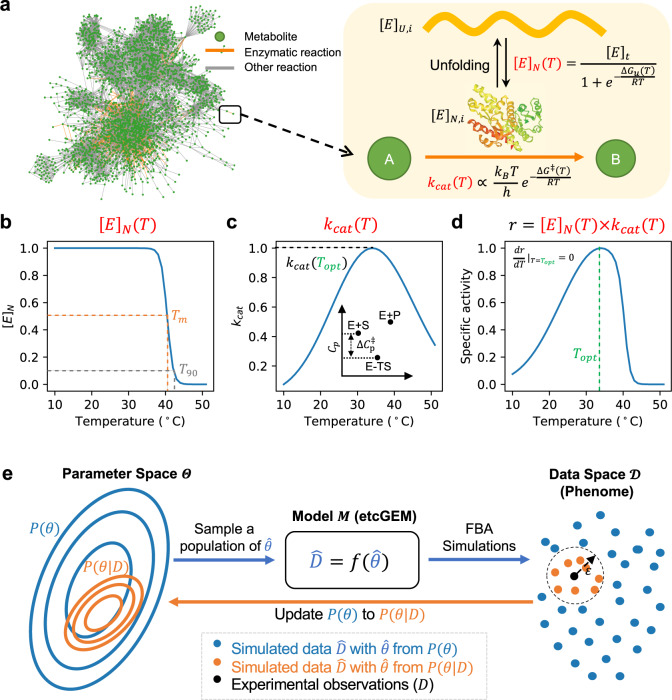
Using Bayesian statistical learning to integrate temperature dependence in enzyme-constrained GEMs. **a** An illustration of the temperature effects on enzyme-catalyzed reactions and their integration into an etcGEM (see detailed description and equations in “Methods” section). The metabolic network ecYeast7.6^16^ is shown. **b** A two-state denaturation model^20,21,58^ was used to describe the temperature-dependent unfolding process. [*E*]_*N*_ is the concentration of the enzyme in the native state; *T*_opt_ is the optimal temperature at which the specific activity is maximized; *T*_m_ and *T*_90_ are temperatures at which there is a 50 and 90% probability that an enzyme is in the denatured state, respectively. **c** Macromolecular rate theory^31,33^ describing the temperature dependence of enzyme *turnover number k*_cat_. Inset shows the heat capacity difference between ground state (E + S) and transition state (E − TS), adapted from Hobbs et al.^31^. **d** Temperature dependence of enzyme-*specific activity r*, which is a product of (**b**) and (**c**). **e** Overview the Bayesian statistical learning approach, where the problem can be formulated as given a *generative model (M)* (enzyme and temperature constrained genome-scale metabolic model, etcGEM in this study) corresponding to a set of parameters *θ* and a set of measurements *D *(phenome data), Bayes’ theorem provides a direct way of updating the *Prior* distribution of parameters *P*(*θ*) to a *Posterior* distribution *P*(*θ*|*D*): $P(\theta |D) = \frac{{P(D|\theta ) \times P(\theta)}}{{P(D)}}$. *P*(*θ|D*) is thereby a less uncertain description of the real *θ*. Since *P*(*D*|*θ*) is, in most applications, computationally expensive or even infeasible to obtain, a sequential Monte Carlo based approximate Bayesian computation (SMC-ABC) approach was implemented (“Methods”) to sample a list of parameter sets from the *Posterior*.

In this study, we developed a novel approach for incorporating temperature dependence into an enzyme-constrained GEM (ecGEM)^16^ (Fig. 1) with the resulting model termed enzyme and temperature constrained GEM (etcGEM). The approach combined the following steps: (i) etcGEM construction (Fig. 1a–d), (ii) flux balance analysis (FBA), and (iii) Bayesian statistical learning (Fig. 1e). The ecGEM, which includes, besides the traditional stoichiometric matrix, also enzyme abundances and activities, provided an excellent template to directly integrate the enzyme temperature effects. Firstly, for a given reaction, the flux cannot exceed the capability of the enzyme, which is defined as the product of the functional enzyme concentration [*E*]_*N*_ and its *k*_cat_. Secondly, the total amount of enzymes that the cell can afford is also limited^27^. Inclusion of temperature constraints into ecGEM was thus achieved by making [*E*]_*N*_ and *k*_cat_ temperature-dependent, and by incorporating the additional cost of enzymes in the denatured state (Fig. 1a, “Methods”). Three thermal parameters were required for each enzyme in the resulting etcGEM, including (i) the melting temperature *T*_*m*_ (Fig. 1b), (ii) the heat capacity change ${\Delta}C_p^\ddagger$ (Fig. 1c), and (iii) the optimal temperature *T*_opt_ (Fig. 1d, “Methods”). Moreover, to capture the temperature effects on the energy cost of non-growth associated maintenance (NGAM), a temperature-dependent NGAM expression term was estimated from experimental data and included in the model.

To resolve the challenges arising from the uncertainties in the parameter values, we used Bayesian statistical learning^26^, which is a probabilistic framework that has been successfully applied for quantifying and reducing uncertainties in various fields, including deep learning^28^, ordinary differential equations^29^, and biochemical kinetic models^30^. The approach uses experimental observations (*D*) to update *Prior* distributions (*P*(*θ*)) of model parameters to *Posterior* ones (*P*(*θ|D*)) (Fig. 1e). We refer to the model equipped with *θ* sampled from *P*(*θ*) or *P*(*θ*|*D*) as a *Prior* or *Posterior* etcGEM, respectively. The resulting Posterior etcGEMs provided a more reliable platform to study the thermal dependence of cell metabolism, with an inherent benefit that the uncertainty in the interpretation and prediction from the improved *Posterior* etcGEMs could also be quantified.

### Bayesian modeling improves etcGEM performance by reducing parameter statistical uncertainties

We next applied the above approach to model the temperature dependence of yeast metabolism (Fig. 1). This was done by incorporating temperature effects into the ecYeast7.6^16^ model and the resulting model was termed etcYeast7.6. Enzyme *T*_*m*_ and *T*_opt_ parameters were either collected from literature or predicted by machine-learning models (“Methods”). The heat capacity change ${\Delta}C_p^\ddagger$ was estimated as −6.3 kJ/mol/K by fitting the macromolecular rate theory to the yeast specific growth rate at various temperatures^31^ and then applied for all enzymes. As a result, the etcYeast7.6 model was obtained with an expansion of 2292 temperature-associated parameters for a total of 764 metabolic enzymes (Fig. 1a). The temperature dependence of NGAM was inferred from experimental data (“Methods”, Supplementary Fig. 1). To assess the quality of the models, three datasets were used that included (i) the maximal specific growth rate in aerobic batch cultivations (number of temperature points *n* = 8)^4^, (ii) anaerobic batch cultivations (*n* = 8)^5^, and (iii) fluxes of carbon dioxide (CO_2_), ethanol, and glucose in chemostat cultivations (*n* = 6)^23^ (Supplementary Fig. 2, “Methods”).

We observed that etcYeast7.6 predictions made using the initial parameter values could only accurately reproduce the experimental observations when the temperature was lower than 30 °C, while the method failed at temperatures above 30 °C (Supplementary Fig. 2). This was assumed to occur since at lower temperatures, the temperature dependence of enzyme *k*_cat_ values is the major determining factor, while at higher temperatures there are additional factors, such as the protein denaturation and increased energy for maintenance, that affect the cell growth^5^ (Fig. 1b, c). Particularly, the metabolic shift (Supplementary Fig. 2c) happens within about 2° (36–38 °C) and accurate prediction of this metabolic flux shift may require more precise enzyme parameter values. Moreover, we measured a high level of uncertainty associated with the initial parameter values, as the average standard deviation was estimated as 3.4 and 5.9 °C for enzymes with experimentally measured *T*_*m*_ and those without experimentally measured *T*_*m*_, respectively, and increased up to 13 °C with the *T*_opt_ values predicted by machine learning (“Methods”). Another potential source of error was due to assuming the same ${\Delta}C_p^\ddagger$ values for all enzymes.

**Fig. 2:**
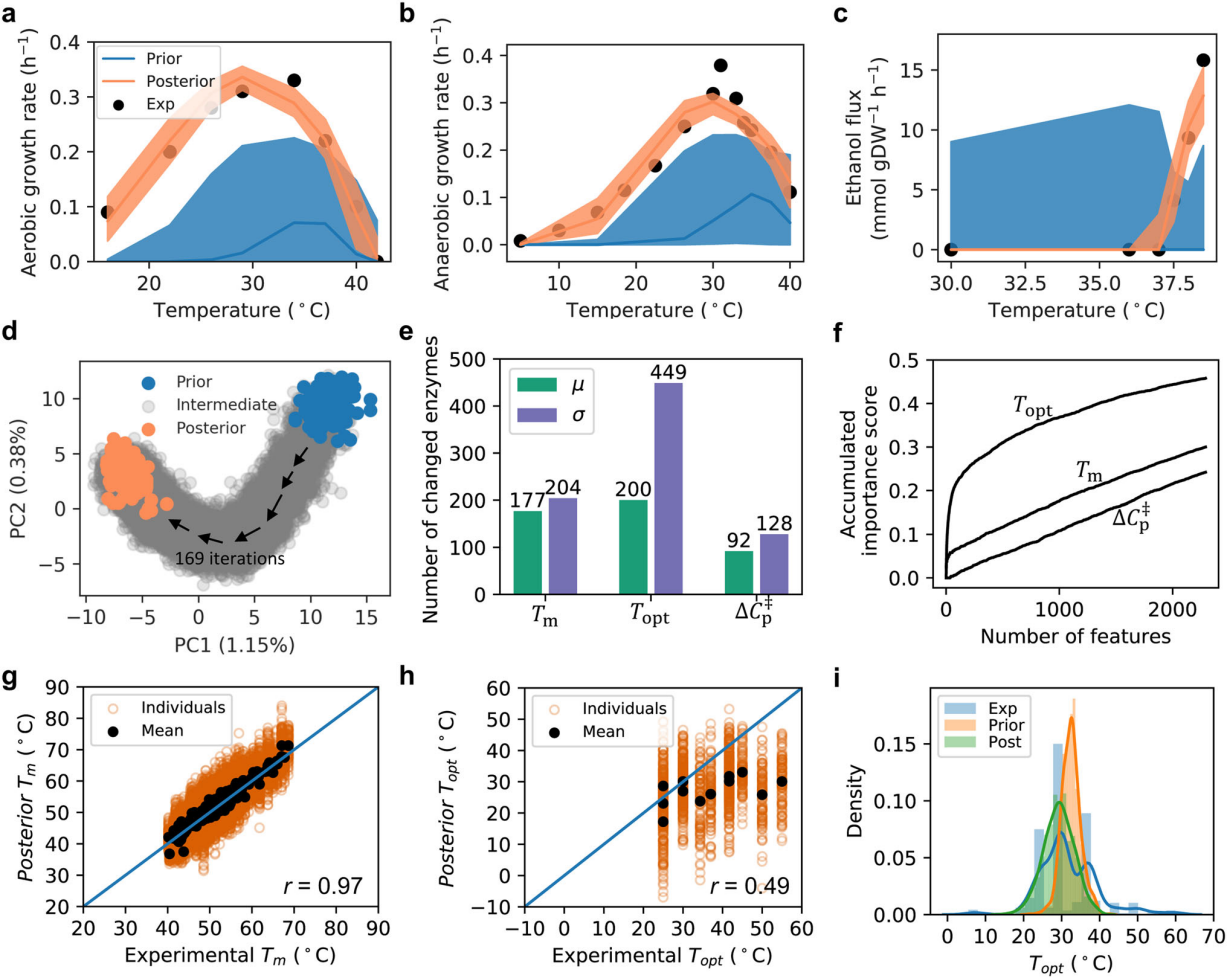
Bayesian modeling improves etcGEM performance by reducing parameter statistical uncertainties. **a**, **b** Simulated (**a**) aerobic and (**b**) anaerobic growth rates in batch cultivations at various temperatures with *Prior* and *Posterior* etcGEMs. **c** Simulated ethanol secretion flux in a chemostat at various temperatures. In **a**–**c**, lines indicate median values and shaded areas indicate regions between the 5th and 95th percentiles (*n* = 128 for *Prior* and *n* = 100 for *Posterior* models). **d** Principal component analysis (PCA) 21,504 parameter sets ($\hat \theta$) sampled in the Bayesian approach. Each parameter in the set θ^*^ was standardized by subtracting the mean and then be divided by the standard deviation before PCA. $\hat \theta$ of 128 *Prior* and 100 *Posterior* etcGEMs are highlighted in blue and orange, respectively. All other $\hat \theta$ were termed as “intermediate” and marked in gray. **e** The number of enzymes, out of all 764, with a significantly changed mean (Šidák adj. Welch’s *t* test *p* value < 0.01, two-sided) and variance (Šidák adj. one-tailed *F*-test *p* value < 0.01) in *T*_*m*_, *T*_opt_, and ${\Delta}C_p^\ddagger$ between *Prior* and *Posterior*. Parameters from 128 *Prior* and 100 *Posterior* etcGEMs were used for statistical tests. **f** A random forest model was used to score the importance of all 2292 parameters during the Bayesian approach (“Methods”). The plot shows the accumulated importance score for each of the three-parameter categories. **g** Parity plot comparing the experimental *T*_*m*_ values and ones in the 100 *Posterior* models. **h** Parity plot comparing the experimental *T*_opt_ values from BRENDA and ones in the 100 *Posterior* models. In **g**, **h**, *r* denotes the *Pearson*’s correlation coefficient between experimental values and *Posterior* mean values. **i** Comparison among distributions of the mean of *Prior T*_opt_ s, mean of *Posterior T*_opt_ s (Post) and experimental *T*_opt_ s (Exp) from BRENDA. The bimodal distribution of *T*_opt_ values is due to the common values in BRENDA occurring at room temperature 25 °C (111 times), the optimal growth temperature of yeast 30 °C (244 times), and 37 °C (142 times). Source data are provided as a Source Data file.

We, therefore, applied the Bayesian statistical learning approach. First, a threefold cross-validation using the above three datasets, showed that there was both overlapping and orthogonal information among the datasets (Supplementary Fig. 3), suggesting that all three datasets should be used for updating the model parameters. Next, the three datasets were combined and split into training (50%) and test (50%) datasets based on the temperature points in each dataset (Supplementary Fig. 4). The training dataset was used to update the *Prior*, which was then tested on the test dataset after each iteration (Supplementary Fig. 4: $R_{\rm{test}}^2$ increased proportionally with the $R_{\rm{train}}^2$). After ~80 iterations, the *Posterior* models achieved a median $R_{\rm{train}}^2$ score of 0.90 (5–95% percentile range: [0.89–0.93]) and a median $R_{\rm{test}}^2$ score of 0.84 (5–95% percentile range: [0.71–0.91]), demonstrating the high generalizability of the *Posterior* models obtained from the SMC-ABC approach. Finally, we used all three datasets to update the *Prior* to *Posterior* and sampled 100 *Posterior* etcGEMs, where each model achieved an average *R*^2^ higher than 0.9 on all three datasets (Supplementary Fig. 5) and could therefore accurately describe the observed measurements (Fig. 2a–c and Supplementary Fig. 6). The increased performance on all three datasets clearly demonstrated the need to update the parameter *Prior* distribution to a *Posterior* one.

We next explored which parameters had been updated in the Bayesian approach. The principal component analysis applied to the 21504 parameter sets generated in the approach showed a clear trend of how the *Prior* distributions were gradually updated to distinct *Posterior* distributions, despite the first two components explaining less than 2% of the total data variance (Fig. 2d). Further comparison between *Prior* and *Posterior* distributions revealed that in all three parameter categories, a reduced variance in the updated parameters was more likely than a change in mean values (Fig. 2e, protein-wise comparison shown in Supplementary Fig. 7). Particularly for enzyme *T*_opt_ s, a significant (Šidák adj. one-tailed *F*-test *p* value < 0.01) reduction in variance was observed with 59% (449/764), whereas a significant (Šidák adj. Welch’s *t* test *p* value < 0.01) change in the mean value was found with merely 26% (200/764). Importantly, we observed that the approach tended to change the enzyme *T*_opt_ rather than its *T*_*m*_ and ${\Delta}C_p^\ddagger$ parameters (Fig. 2e). In addition, a machine learning approach (“Methods”) further revealed that, out of all three parameter types, the largest contribution to the improved *Posterior* etcGEM performance during the Bayesian approach was from enzyme *T*_opt_ s (Fig. 2f). A note, even though the uncertainties of most parameters were reduced (Fig. 2e), there were still big uncertainties in the *Posterior* models (e.g., *T*_opt_, 10.9 vs. 7.1 °C; *T*_*m*_, 4.9 vs. 4.0 °C; ${\Delta}C_p^\ddagger$, 2.0 vs. 1.8 kJ/mol/K, average standard variance comparison between *Prior* and *Posterior*, Supplementary Fig. 7).

To evaluate the *Posterior* parameter sets, the parity plot comparing the *T*_*m*_ in the *Posterior* models and experimental values used in the *Prior* showed that the *Posterior* mean values were strongly correlated with the experimental ones (Pearson’s *r* = 0.97, *p* value < 1e−32) (Fig. 2g). This indicated that the parameter values returned by the SMC-ABC approach, and with which the model fit the experiment data well, were not very different from the experimentally measured values. We also compared the *T*_opt_ values in the *Posterior* models to the experimental data collected from BRENDA, where 14 enzymes with known *T*_opt_ in BRENDA could be mapped to the etcYeast model based on their UniProt IDs. A weak correlation (Pearson’s *r* = 0.49, *p* value = 0.075) was found between the *Posterior* mean values and the experimental data (Fig. 2h). Further comparison of the distribution of *Posterior* mean values of all enzymes and 662 enzymes *T*_opt_ records for *S. cerevisiae* in BRENDA showed that the *Posterior* *T*_opt_ values were more similarly distributed to the experimental *T*_opt_ values than were the original *Prior* values, showing that the SMC-ABC approach indeed improved the estimation of *T*_opt_ values (Fig. 2i).

### The yeast growth rate is explained by temperature effects on its enzymes

**Fig. 3:**
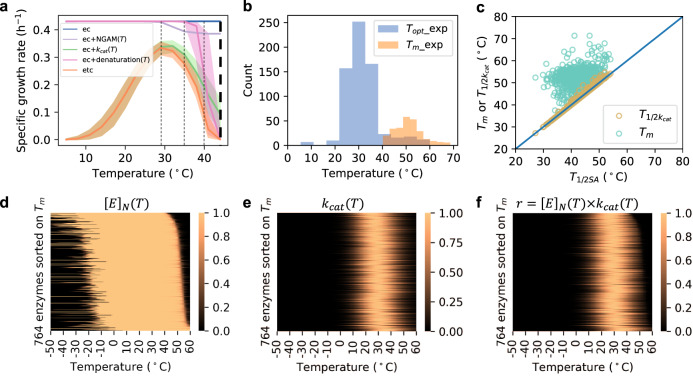
Yeast growth rate is explained by temperature effects on its enzymes. **a** Illustration of how the temperature dependence of different processes combines to affect the growth rate. Fig. legend: ec—predictions with the enzyme constrained model; ec+NGAM(*T*)—incorporates the temperature effects on nongrowth associated maintenance into the ec model (Supplementary Fig. 1); ec+*k*_cat_(*T*)—incorporates the temperature effects on enzyme *k*_cat_ values into the ec model; ec+denaturation(*T*)—incorporates the temperature effects on enzyme denaturation into the ec model; etc—enzyme and temperature constrained model that includes the temperature effects on NGAM, *k*_cat_ and enzyme denaturation into ec model. The growth rate at each temperature point was simulated with all 100 *Posterior* etcGEMs. Lines indicate median values and shaded areas indicate regions between the 5th and 95th percentiles (*n* = 100). **b** Comparison between distributions of experimentally measured enzyme *T*_opt_ values (*n* = 662) from BRENDA^35^ and enzyme *T*_*m*_ values (*n* = 265) from Leuenberger et al.^7^ in *S. cerevisiae*. **c** Comparison between *T*_1/2*SA*_, the temperature at which the specific activity is 50% of its maximum, and *T*_1/2*k*cat_, the temperature at which the *k*_cat_ value is 50% of its maximum, and *T*_*m*_ in the *Posterior* models. **d** Probability of 764 enzymes in the native state. From top to bottom, the enzymes showed increased *s*. Each pixel represents one probability value of an enzyme at a specific temperature. **e** Normalized *k*_cat_ values of 764 enzymes at different temperatures. Each pixel represents one normalized value of an enzyme at a specific temperature. **f** Normalized specific activities of 764 enzymes at different temperatures. The values in (**f**) are products of (**d**) and (**e**). In **d**–**f**, the same ordering of enzymes is shown. Source data are provided as a Source Data file.

With the *Posterior* etcGEMs capable of describing various experimental observations (Fig. 2a–c), we analyzed how the temperature effects on each of the three processes—NGAM, *k*_cat_ and the protein denaturation process—contribute to whole-cell growth (Fig. 3a). We observed that, at temperatures below 29 °C, the temperature-dependent *k*_cat_ was the only factor that affected the cell growth rate. In the range between 29 and 35 °C, both *k*_cat_ and NGAM determined the growth rate. The contribution of enzyme denaturation to the temperature dependence of cell growth, however, was observed only at temperatures higher than 35 °C, with the denaturing effect becoming the dominant effect at ~40 °C and lack of cell growth at 42 °C. Therefore, in contrast to previous reports indicating that an over tenfold increase in NGAM cost with the temperature change from 30 to 33 °C was the major limiting factor to cell growth^5,32^, our modeling approach showed that the increased NGAM has a merely moderate effect on growth rate (Fig. 3a).

Interestingly, the temperature dependence of enzyme *k*_cat_ *s* alone could explain the temperature dependence of cell growth below 35 °C, including the decline in cell growth right after the optimal growth point defined by OGT. According to the macromolecular rate theory^31,33^, *k*_cat_ degeneration at temperatures above the optimal point can be attributed to the negative values of ${\Delta}C_p^\ddagger$ for enzyme catalysis. This can explain the negative curvature of enzyme-specific activities in the absence of the denaturation process^31,33,34^. Given that experimentally measured enzyme melting temperatures (*T*_*m*_) are on average 20 °C higher than enzyme *T*_opt_ *s* collected from BRENDA^35^ (Fig. 3b), protein denaturation alone seems to be insufficient to explain the thermal mechanism underlying enzyme *T*_opt_ *s*. In addition, all posterior *T*_opt_ *s* showed a similar distribution as experimental *T*_opt_ *s*, even though the etcGEM had never seen those experimental *T*_opt_ *s* (Fig. 2i), which supported our use of the macromolecular rate theory in the model. In the *Posterior* models, the degeneration of enzyme-specific activities clearly depended on the *k*_cat_ degeneration instead of protein denaturation (Fig. 3c). This indicates that *k*_cat_ degeneration, in addition to protein denaturation, plays an important role in the temperature dependence of yeast cell growth.

We further observed that, even though the model contained only 764 enzymes from a total of ~6700 proteins^36^, protein denaturation alone could still explain the termination of cell growth at 42 °C (Fig. 3a). However, in the *Posterior* etcGEMs, only 9 enzymes (1%) with a mean melting temperature below 42 °C were present (ERG1, ATP1, ALA1, KRS1, SER1, HEM1, PDB1, ADH1, and TRP3) (Supplementary Fig. 8), of which three (ATP1, HEM1, and PDB1) are located in the mitochondria^37^. The other enzymes remained in the native state even at temperatures several degrees higher than 42 °C (Fig. 3d), though they were enzymatically active only in the temperature window of cell growth between 10 and 42 °C (Fig. 3f), due to the low *k*_cat_ values beyond this temperature range (Fig. 3e). Nearly all yeast enzymes were found to be cold-denatured only at temperatures lower than 0 °C, which is beyond the temperature window for cell growth (Fig. 3d), although there have been studies showing that some yeast proteins have a cold-induced denaturation above 0 °C^38^. This may be due to the fact that the experimental data used to improve the model performance were mainly obtained in the supra-optimal temperature range, with few data available from the suboptimal range.

### Metabolic shifts are explained by temperature-induced proteome constraints

**Fig. 4:**
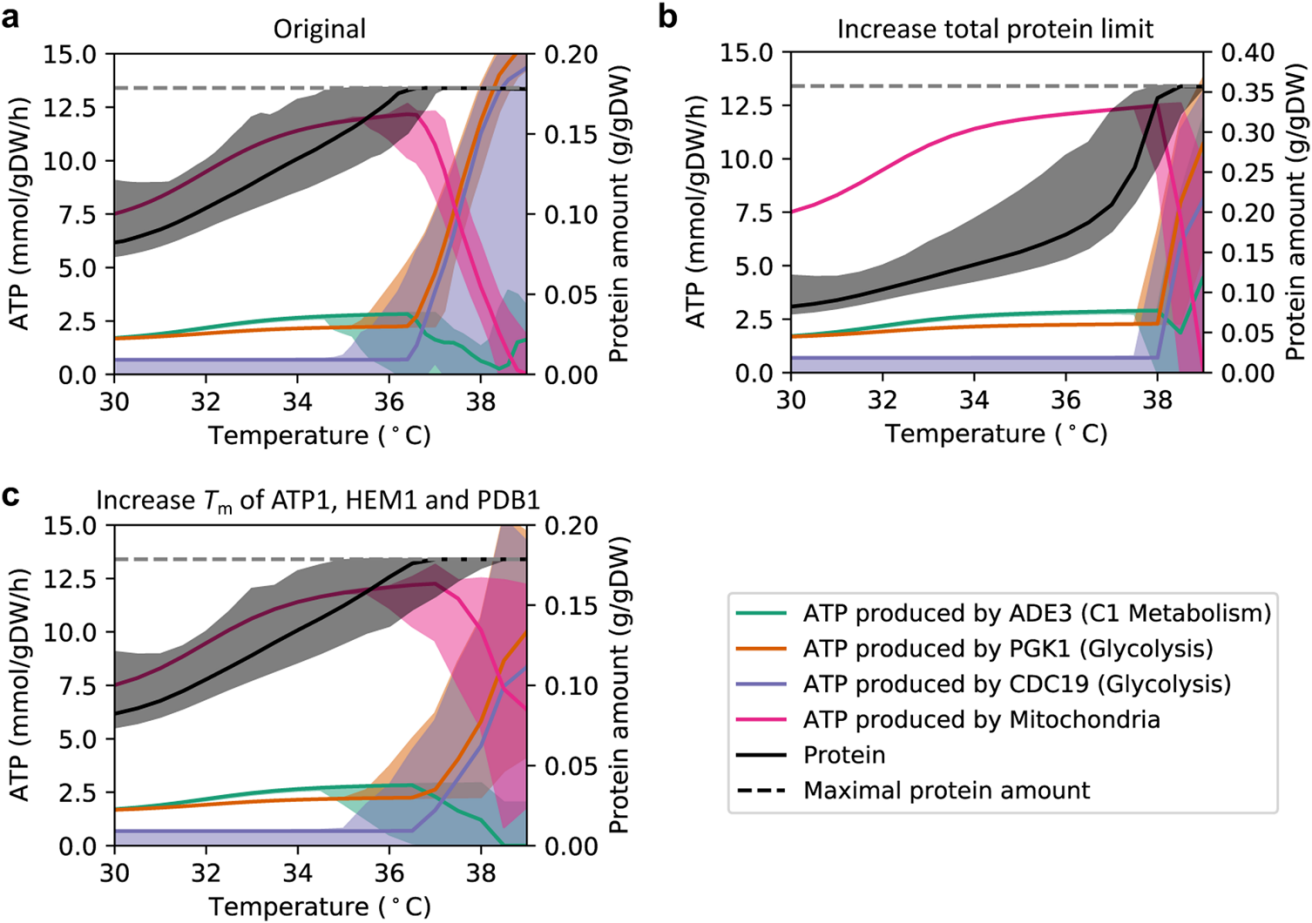
Metabolic shifts are explained by temperature-induced proteome constraints. The ATP production in the cytoplasm and the total protein amount required at different temperatures were simulated using *Posterior* etcGEMs with chemostat culture settings with a dilution rate of 0.1 h^−1^ (“Methods”). Simulation results from **a** the original *Posterior* models, **b** the original *Posterior* models with doubled total protein constraint, **c** the original *Posterior* models with increased *T*_*m*_ of ATP1, HEM1, and PDB1 (to 50 °C) and are shown. Lines indicate median values and shaded areas indicate the region between the 5th and 95th percentile (*n* = 100). Source data are provided as a Source Data file.

Published reports show that at temperatures above 37 °C in chemostat cultures with a dilution rate of 0.1 h^−1^, yeast shifts its metabolism from a completely respiratory one to a partly fermentative one, which is also accompanied by a large increase in glycolytic flux^23^. Since our updated *Posterior* etcGEMs are able to simulate this metabolic shift (Fig. 2c and Supplementary Fig. 6), we used them to further explore the mechanisms behind the observed process. We observed that the shift occurs due to a proteome constraint, meaning that the total protein level in the cell reaches an upper bound (Fig. 4a). The proteome constraint occurs due to the decrease in enzyme specific activities with increasing temperature (Fig. 3f) and since the maximal protein amount in the cell is limited^27^. As a result, the cell has to synthesize more enzymes to maintain cell growth at the given growth rate (Fig. 4a) until the enzyme amount hits the upper bound. Doubling this protein constraint can delay the shift point from 37 to 38 °C (Fig. 4b and Supplementary Fig. 9). These findings are consistent with earlier studies showing that the activation of the Crabtree effect in chemostat cultures at 30 °C is due to a proteome constraint^16,39^. When the temperature increases above 36 °C, ATP production by glycolysis is dramatically increased, while ATP production by the mitochondria decreases (Fig. 4a). Even though the respiratory pathway produces more ATP per glucose amount, the fermentative pathway produces more ATP per protein mass and therefore becomes more energetically efficient when the cell reaches a proteome constraint^39^. In addition, three key mitochondrial enzymes (ATP1, HEM1, and PDB1) (Supplementary Fig. 8) were found to be unstable, which makes the respiratory pathway even more resource-inefficient for ATP production. When increasing the *T*_*m*_ of these three enzymes to 50 °C, the same shift could still be observed while the ATP production shift from mitochondria to glycolysis was reduced (Fig. 4c and Supplementary Fig. 9).

### etcGEM uncovers growth rate-limiting enzymes

**Fig. 5:**
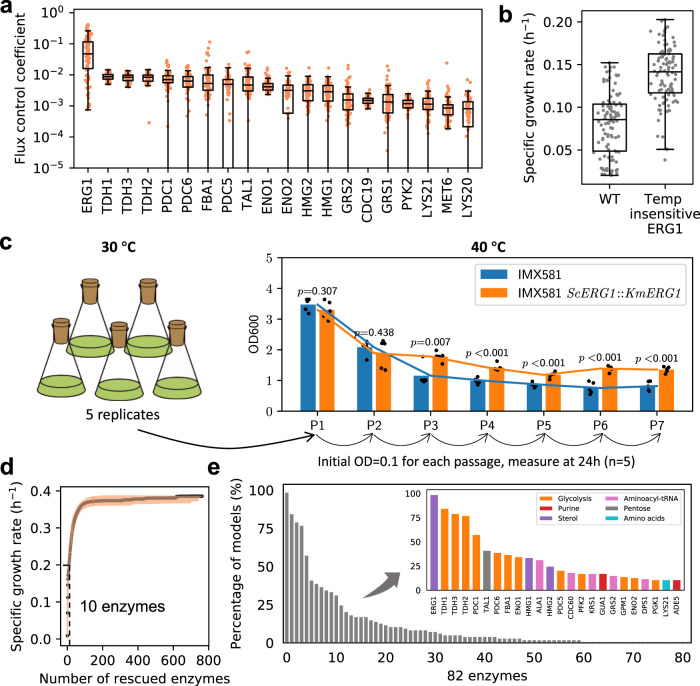
etcGEM uncovers growth rate-limiting enzymes. **a** Twenty enzymes with the highest flux sensitivity coefficients at 40 °C (*n* = 100 for each enzyme). **b** Predicted maximal specific growth rate of wild-type yeast and the one without any temperature constraints (fully functional) on ERG1 enzyme at 40 °C (*n* = 100 for each strain). In **a**, **b**, centerline, median; box limits, upper and lower quartiles; whiskers, 1.5× interquartile range; Each dot represents the prediction from one *Posterior* etcGEMs. **c** The effect of KmERG1 expression on thermotolerance in *S. cerevisiae*. The strains were passaged for 7 times (24 h for each passage) to obtain stable growth at 40 °C. Optical densities (600 nm) are shown at 24 h. Data are represented as mean values of five replicates in the bar chart. Dots represent the values of five replicates. *p* Values denote Welch’s *t* test (two-sided). The exact *p* values for P1–P7 are 0.307, 0.438, 0.0066, 0.00020, 0.00046, 0.00015, 0.00018, respectively. **d** Simulated maximum specific growth rate by removing the temperature constraints of most rate-limiting enzymes at each step in each *Posterior* etcGEM at 42 °C. Lines indicate median values and shaded areas indicate the region between the 5th and 95th percentiles (*n* = 100). **e** The percentage of *Posterior* etcGEMs (*n* = 100) predicts an enzyme to be in the minimal enzyme set required to be fully functional at 42 °C in order to achieve a maximal specific growth rate of 0.2 h^−1^. Inset shows the names and pathways of genes predicted by more than *Posterior* etcGEMs 10% they are involved in. Source data are provided as a Source Data file.

To investigate which enzymes limit the cell growth at superoptimal temperatures, the flux sensitivity coefficient of each enzyme was calculated (“Methods”). Among all the enzymes in the model, the squalene epoxidase ERG1 displayed order of magnitude higher median flux sensitivity coefficient than other enzymes, indicating that it is the most flux-controlling enzyme at 40 °C (Fig. 5a) and above (Supplementary Fig. 10). Furthermore, the removal of the temperature constraint on ERG1 increased the simulated specific growth rate from 0.09 to 0.14 h^−1^ (Fig. 5b). We, therefore, evaluated the impact of replacing the wild-type *ERG1* gene with *ERG1* from the thermotolerant yeast *Kluyveromyces marxianus* (KmERG1, “Methods”). At first, at the lethal temperature of 42 °C, only a small improvement in growth rate (from 0.01 to 0.06 h^−1^) was predicted and no significant growth difference was detected between the wildtype and the strain with kmERG1 (Supplementary Fig. 11). However, already after 2 passages of adaptation at 40 °C, the strain with KmERG1 indeed showed significantly better growth than the wild type (Fig. 5c).

The reduced growth rate at 42 °C is likely caused by an impaired function of several different enzymes, and rescuing a single enzyme is insufficient to improve the growth rate. Therefore, in order to characterize the set of growth rate-limiting enzymes at 42 °C, we gradually removed the temperature constraints on enzymes (set *k*_cat_ and denaturation temperature independent) in the order of decrescent flux sensitivity coefficient values in each of the *Posterior* etcGEMs. Interestingly, in the case of recovering the cell growth rate to 0.2 h^−1^, we found an agreement among all *Posterior* etcGEMs that ten enzymes are required to be fully functional at 42 °C (Fig. 5d). Since each model predicted a different subset of such enzymes, an ensemble approach was used to count the number of models (votes) in which an enzyme is predicted to be one of ten such enzymes (Fig. 5e). In total, 82 enzymes were predicted by at least 1 *Posterior* etcGEM, and only 24 (out of 82) enzymes were each predicted by more than 10% of the *Posterior* etcGEMs (Fig. 5e, inset). Among these 24 enzymes, 12 enzymes were engaged with Glycolysis and 3 enzymes were involved in sterol biosynthesis: ERG1, and HMG1,2 catalyzing the flux-controlling steps in sterol biosynthesis^40^. The remaining enzymes were mainly involved in DNA or protein synthesis related pathways.

## Discussion

Here, we present a Bayesian genome-scale modeling approach to resolve the temperature dependence of cellular metabolism. Using an ecGEM^16^ as a template, we modeled the temperature effects on each individual enzyme by including temperature-dependent terms for the independent processes of denaturation as well as catalysis (Fig. 1a). Due to the high level of uncertainty and low accuracy associated with the initial thermal parameter values (Supplementary Fig. 7), which were a result of experimentally measured noise or variability arising from machine learning or theoretical predictions, the model predictions initially could not correctly recapitulate experimental observations (Fig. 2a–c and Supplementary Fig. 2). We, therefore, used Bayesian statistical learning that enabled updating our *Prior* guess of the highly uncertain thermal parameters to a more accurate *Posterior* estimation of these parameters according to observed phenotypic data (Fig. 1e). The resulting *Posterior* etcGEMs accurately describe the experimental observations (Fig. 2a–c) and thus provide a more reliable platform to study the thermal dependence of yeast metabolism.

Previous studies modeling the temperature dependence of enzyme activities have relied mainly on protein denaturation and the Arrhenius equation, where protein denaturation explained the negative curvature for the temperature dependence of enzyme activity^20,21^. However, with the increasing amount of evidence showing that protein denaturation alone is insufficient to explain the decrease in enzyme specific activity above *T*_opt_, macromolecular molecular rate theory^31,34^ has become a promising alternative. It was successfully applied to many enzymes^31,33,34^, including its use in explaining the evolution of enzyme catalysis^34^. According to the theory, a negative heat-capacity change (${\Delta}C_p^\ddagger$) exists between the transition state and the ground state in the enzyme catalytic process (Fig. 1c), which leads to a negative curvature for the temperature dependence of enzyme activity in the absence of denaturation^31^. We found that with this theory, the temperature dependence of *k*_cat_ *s* acts as a major contributor to the cell growth rate at all temperatures, which can especially explain the decline in cell growth rate right after the OGT (Fig. 3a). Yeast enzymes only maintain high *k*_cat_ *s* in the temperature window of cell growth (Fig. 3e), which means that the metabolism becomes inefficient at superoptimal temperatures due to the general decrease in enzyme turnover without denaturations (Fig. 3d–f).

Using the Bayesian genome-scale modeling approach to quantitatively depict the temperature effects on yeast metabolism led to insights into the long-standing discussion on the roles of different cellular factors in cellular fitness under heat stresses^4,5,7,23,41^. For instance, protein denaturation has been suspected as one of the main causes of the decline in cell growth beyond the OGT point. However, recent high throughput measurements of melting temperatures (*T*_*m*_) for 707 *S. cerevisiae* proteins revealed a *T*_*m*_ distribution with a mean value of 52 °C and a minimum of 40 °C^7^, which suggests that protein denaturation alone might not be sufficient to explain the decline of yeast cell growth between 30 °C (OGT) and 42 °C (lethal temperature point). An alternative explanation is provided by the evidence of a significant increase of NGAM observed with yeast cells grown in anaerobic chemostat cultivations at high temperatures (33–40 °C) compared to ones grown at low temperatures (5–31 °C)^5^, which suggests an imbalance in cellular energy allocation in the superoptimal temperature range. Quantitative assessment using our modeling approach revealed that impaired cell growth is caused by a combination of decreased *k*_*cat*_ values, increased NGAM costs, and protein denaturation (Fig. 3). Furthermore, between 30 and 35 °C, the combined decrease in *k*_cat_ s and increase in NGAM explains the decline in cell growth, whereas, with temperatures above 35 °C, protein denaturation becomes the dominant factor, causing cell death at 42 °C. However, in accordance with published findings that cellular proteomes have a broad distribution of protein stability with only proteins at the tail of the distribution being problematic^42^, using our approach we identified only ~1% unstable enzymes denatured at the lethal point (*T*_*m*_ lower than 42 °C, Fig. 3d).

We identified two interesting metabolic pathways involved in yeast thermotolerance: sterol metabolism and mitochondrial energy metabolism. With sterol metabolism (Fig. 5d), it is known that high sterol levels help yeast cells survive under heat stress^43^ and changes of the sterol composition of the yeast membrane from ergosterol to fecosterol^44^ can significantly increase yeast thermotolerance. However, yeast was found to downregulate its whole ergosterol biosynthesis at both transcription and translation levels when increasing the temperature from 30 to 36 °C (Supplementary Fig. 12). Our modeling approach identified three problematic enzymes (Fig. 5d: HMG1,2 and ERG1) in the sterol metabolism, which are also flux-controlling enzymes in the sterol biosynthesis pathway^45^. We experimentally confirmed that the replacement of ERG1 with its ortholog in the thermotolerant yeast *K. marxianus* can significantly improve the cell growth at 40 °C (Fig. 5c). Further simulations by downregulating the genes involved in ergosterol pathways (except ERG1) showed that when these enzymes were down-regulated by a percentage higher than ~40%, a temperature-insensitive ERG1 can rescue the cell growth, while it failed when they were downregulated to less than 40% (Supplementary Fig. 13). We thereby hypothesize that, since those three enzymes are problematic at superoptimal temperatures, there is no need for the cell to maintain high expression and translation levels of other enzymes in the same pathway. Instead, it has to downregulate its whole ergosterol biosynthesis to save resources and increase fitness.

With mitochondria, previous studies have indicated that the mitochondrial genome plays an important role in yeast thermal adaptation^46–48^. We found that out of the nine unstable enzymes identified with the *Posterior* etcGEMs (with a *T*_*m*_ lower than 42 °C, Supplementary Fig. 8), three (ATP1, HEM1, and PDB1) belonged to the mitochondrial energy metabolism. Simulation of chemostat data (Fig. 4) revealed that at superoptimal temperatures, yeast prefers to produce ATP via the glycolysis metabolism instead of the mitochondrial energy metabolism in the mitochondria. This can be explained by the limited total protein content in the cell and resource-inefficient mitochondrial energy metabolism. A previous study also found that a mitochondrial matrix protein Mge1p, which is essential for mitochondrial functions, loses its functions at temperatures higher than 37 °C due to denaturation and dimer dissociation^49^. Another study found that the mitochondrial inner membrane is severely affected at 38 °C and may impair the function of mitochondria^50^. Furthermore, mitochondria only exist in eukaryotes and almost all of them have evolved to have an OGT below 40 °C^3^. All these findings indicate that mitochondria are not evolved to be functional at very high temperatures. Since mitochondrial energy metabolism is not essential for yeast cell growth, as there are alternative energy pathways (Fig. 4), this also explains why we could not successfully predict mitochondrial enzymes to be engineering targets for the recovery of cell growth at 42 °C (Fig. 5d), despite the existence of three unstable enzymes in the mitochondrial energy metabolism.

As a note, the assumption of two-state denaturation and macromolecular theory to describe the temperature dependence of enzyme activity may be oversimplified for some enzymes. The experimental data used to update the *Prior* guesses of thermal parameters was mainly from the superoptimal temperature range (Fig. 2a–c), whereas there are still big uncertainties in the current *Posterior* (Supplementary Fig. 7). These all indicate that the etcYeast7.6 can still be enhanced in the future by (i) improving the formulation of enzyme temperature-dependence and (ii) more experimental data for model validation and uncertainty reduction.

In conclusion, we demonstrate the usefulness of a Bayesian genome-scale modeling approach for reconciling temperature dependence of yeast metabolism. Describing the link between temperature and cell physiology is of industrial importance, e.g., for finding optimized production of biochemicals^24,51–53^, but also in medicine, e.g., to understand the effects of temperature on human metabolism^54–56^. Furthermore, based on its success here, we foresee that our method can be integrated into genome-scale modeling approaches in general. This approach can also become a staple of GEM modeling in order to resolve uncertainties present in the data, which can be important as GEMs have become a widely used platform for integration of various biological data, such as transcriptomics and proteomics data that are associated with large uncertainties^57^.

## Methods

### The temperature-dependent enzyme-constrained genome-scale metabolic model

The central concepts of an enzyme constrained model^16^ are as follows: (1) the flux through each reaction cannot exceed the capacity of its catalytic enzyme: $v_i \le k_{{\rm{cat}},i} \cdot [E]_i$, where [*E*]_*i*_ is the concentration of enzyme *i*; (2) the total enzyme amount is constrained by the experimental measurement: $\sum [E]_i \le [E]_t.$ Once the temperature-dependent denaturation and *k*_cat_ were considered, [*E*]_*i*_ in the first constraint should be [*E*]_*N*,*i*_, which is the concentration of individual active enzymes. [*E*]_*i*_ in the second constraint should be $[E]_{t,i} = [E]_{N,i} + [E]_{U,i}$, which is the total concentration of enzymes in both active and denatured forms (Fig. 1a). In addition, to capture the increased expenditure for maintenance under increased heat stress, a temperature-dependent non-growth-associated ATP maintenance term can be assumed from experimental measurements. In summary, the updated constraints in etcGEM are

1 $$\left\{ \begin{array}{l} {Sv = 0} \\ 0 \le v_i \le k_{{\rm{cat}},i}\left( T \right) \cdot \left[ E \right]_{N,i}\left( T \right) \\ {\sum} {\left( {\left[ E \right]_{N,i}\left( T \right) + \left[ E \right]_{U,i}\left( T \right)} \right)} \le \left[ E \right]_t\\ {\rm{NGAM}}\left( T \right) = f\left( T \right) \end{array} \right..$$

The effect of temperature on *k*_cat_ values can be described with an expanded Arrhenius equation (macromolecular rate theory), by including a nonzero heat-capacity change (${\Delta}C_p^\ddagger$) between the transition state and the ground state of the enzyme catalytic process^31,33^:

2 $$k_{\rm{cat}}\left( T \right) \propto \frac{{k_BT}}{h}e^{ - \frac{{{\Delta}G^\ddagger \left( T \right)}}{{\rm{RT}}}},$$

in which *k*_B_ is the Boltzmann constant, *h* is Planck’s constant, *R* is the universal gas constant and ${\Delta}G^\ddagger (T)$ is the free energy difference between the ground state and the transition state. The latter can be expanded as

3 $${\Delta}G^\ddagger (T) = {\Delta}H_{T_0}^\ddagger + {\Delta}C_p^\ddagger \left( {T - T_0} \right) - T\left( {{\Delta}S_{T_0}^\ddagger + {\Delta}C_p^\ddagger {\rm{ln}}\left( {\frac{T}{{T_0}}} \right)} \right),$$

where ${\Delta}H_{T_0}^\ddagger$, ${\Delta}S_{T_0}^\ddagger$, and ${\Delta}C_p^\ddagger$ are the differences in enthalpy, entropy, and heat capacity change between the transition and ground states, respectively, and *T*_0_ is the reference temperature. This theory has been successfully applied to study the temperature dependence of enzyme activity^31,33^ and evolution^34^.

Since there is not enough detailed information regarding the heat-induced denaturation process of yeast proteins, a simple two-state model denaturation was assumed as in many other studies^20,21,58^. In such a model, a protein molecule could be either in a native state (*N*) or a denatured state (*U*), and an equilibrium state was assumed: *N* ↔ *U*. Thereby

4 $$[E]_{N,i} = \frac{1}{{1 + e^{ - \frac{{{\Delta}G_u\left( T \right)}}{{\rm{RT}}}}}}[E]_{t,i},$$

in which $[E]_{t,i} = [E]_{N,i} + [E]_{U,i}$, where [*E*]_*t*,*i*_ is the concentration of enzyme $i$ and Δ*G*_*u*_(*T*) is the free energy difference between the denatured state and the native state and can be expressed as

5 $${\Delta}G_u\left( T \right) = {\Delta}H_u\left( T \right) - T{\Delta}S_u\left( T \right),$$

where Δ*H*_*u*_(*T*) and Δ*S*_*u*_(*T*) are the enthalpy and entropy changes between the denatured and native states at temperature *T*. It has been found that convergence temperatures $T_H^ \ast$ (373.5 K) and $T_S^ \ast$ (385 K) exist for Δ*H*_*u*_ and Δ*S*_*u*_, respectively^41,59,60^. At such temperatures, the Δ*H*_*u*_ and Δ*S*_*u*_ converge to a common value of ΔH^*^ and ΔS^*^. Thereby,

6 $${\Delta}G_u\left( T \right) = {\Delta}H^ \ast + {\Delta}C_{p,u}\left( {T - T_H^ \ast } \right) - T{\Delta}S^ \ast - T{\Delta}C_{p,u}{\rm{log}}\left( {\frac{T}{{T_S^ \ast }}} \right),$$

in which ${\Delta}C_{p,u}$ is the difference in heat-capacity change between the denatured and native states.

In summary, the values of ${\Delta}G^\ddagger (T)$ and Δ*G*_*u*_(*T*) need to be determined in order to model the temperature dependence of enzyme activities, and they can be associated with six unknown parameters: ${\Delta}H_{T_0}^\ddagger$, ${\Delta}S_{T_0}^\ddagger$, and ${\Delta}C_p^\ddagger$ for ${\Delta}G^\ddagger (T)$, and ΔH^*^, ΔS^*^, and Δ*C*_*p*,*u*_ for Δ*G*_*u*_(*T*).

### Computation of thermal parameters

Since it is difficult to directly measure those six thermal parameters (${\Delta}H_{T_0}^\ddagger$, ${\Delta}S_{T_0}^\ddagger$, ${\Delta}C_p^\ddagger$, ΔH^*^, Δ*S*,^*^and Δ*C*_*p*,*u*_) for each enzyme, indirect measurements have to be used to approximate the larger set of thermal parameters. As there are six free variables in the system, six different equations are required to solve for those parameters.

1. At the protein melting temperature *T*_*m*_:
  7 $${\Delta}G_u\left( {T_m} \right)=0.$$
2. At the enzyme optimal temperature *T*_opt_, the enzyme activity is maximized:
  8 $$\frac{{dr}}{{dT}}|_{T = T_{opt}} = 0.$$
  in which *r* = *k*_cat_[*E*]_*N*_;
3. *k*_cat_ at the enzyme optimal temperature *T*_opt_ is known:
  9 $$k_{\rm{cat}}(T_{\rm{opt}}) = \frac{{k_{B}T}}{h}e^{ - \frac{{\Delta}{G^\ddagger (T_{\rm{opt}})}}{RT_{\rm{opt}}}}.$$
4. ${\Delta}C_p^\ddagger$ value can be approximate from the temperature dependence of cell growth rate^31^.
5. We found that there is a very strong linear correlation (*r*^2^ = 0.998, *Pearson*’s correlation) between ΔH^*^ and ΔS^*^ of 116 proteins from Sawle et al.^41^ (Supplementary Fig. 14)
  10 $${\Delta}H^ \ast = 299.58{\Delta}S^ \ast + 20008{\mathrm{J}}/{\mathrm{mol}}$$
6. For some enzymes, *T*_90_, where a 90% possibility exists that an enzyme molecule is in the denatured state, is experimentally measured:
  11 $${\Delta}G_u\left( {T_{90}} \right) = - {\rm{RT}}_{90}{\rm{ln}}9.$$

As a result, the six thermal parameters ${\Delta}H_{T_0}^\ddagger$, ${\Delta}S_{T_0}^\ddagger$, ${\Delta}C_p^\ddagger$ ΔH^*^, ΔS^*^, and Δ*C*_*p*,*u*_ can be obtained by solving the above equations.

In the case of lacking *T*_90_ or failed to obtain a positive Δ*C*_*p*,u_, protein sequence length was used to estimate ΔH^*^ and ΔS^*^^41^ as below:

12 $${\Delta}H^ \ast = \left( {4.0N + 143} \right) \times 1000.$$

13 $${\Delta}S^ \ast = 13.27N + 448.$$

### Sequential Monte Carlo-based approximate Bayesian computation

Approximate Bayesian computation^61^ was applied to infer parameter sets from *Posterior* distributions. Given an observed dataset *D* and a model specified by $\hat \theta$ sampled from the *Prior* distribution *P*(*θ*), if the distance between simulated data $\hat D$ and observed *D* is less than a given threshold ${\it{\epsilon }}$, then this $\hat \theta$ is accepted as the one sampled from $P\left( {\rho \left( {D,\hat D} \right)\; < \;{\it{\epsilon }}} \right)$. $P\left( {\rho \left( {D,\hat D} \right) \;<\; {\it{\epsilon }}} \right)$ is often used to approximate the *Posterior* *P*(*θ*|*D*) when ${\it{\epsilon }}$ is sufficiently small. In the case of high-dimensional parameter space and/or when the *P*(*θ*) is very different from *P*(*θ*|*D*), the acceptance rate would be very low and thus this approach becomes computationally expensive to generate a population of $\hat \theta$ from $P\left( {\rho \left( {D,\hat D} \right) \;< \;{\it{\epsilon }}} \right)$. In this work, a sequential Monte Carlo approach was designed (Supplementary Table 1) to generate a population of $\hat \theta$ sampled from $P\left( {\rho \left( {D,\hat D} \right)\; < \;{\it{\epsilon }}} \right)$. This approach was validated on several toy models with known parameter values and was found to outperform existing other population-based methods when the number of parameters is far greater than the number of samples for fitting (Supplementary Note 1, Supplementary Figs. 16–20).

### Melting temperatures

Among the 764 enzymes included in ecYeast7.6^16^, the *T*_*m*_ (melting temperature) and *T*_90_ (the temperature at which 90% of the protein is in the denatured state) for 266 yeast proteins have been reported previously^7^. For enzymes lacking an experimentally measured *T*_*m*_, a melting temperature of 51.9 °C (the average of existing *T*_*m*_ *s* of 707 yeast proteins) was assumed. In the original paper^7^, the 95% confidence interval was reported for peptides measured in the experiments and the average standard error was estimated at 3.4 °C. This same value was used as the uncertainty measure for the experimentally determined *T*_*m*_ *s*, since the standard error for protein *T*_*m*_ was not available. The *T*_*m*_ of the 266 enzymes was then described with a normal distribution *N*(*T*_*m*,*i*_, 3.4), in which *T*_*m*,*i*_ is the experimentally measured melting temperature of protein *i*. For enzymes that use the mean *T*_*m*_ of 707 proteins^7^ as *T*_*m*_ estimation, the corresponding uncertainty is described as the standard deviation of the 707 *T*_*m*_ *s*, equaling 5.9 °C. Thereby, a normal distribution *N*(51.9,5.9) was used.

### Enzyme optimal temperature

*T*_opt_ values of all enzymes in this study were calculated using a previously developed machine-learning method Tome v1.0 (https://github.com/EngqvistLab/Tome)^22^, which predicts enzyme *T*_opt_ based on primary sequences. This model has a coefficient of determination (*R*^2^ score) of 0.5 on the test dataset. Root-mean-squared error (RMSE) of the prediction was then estimated with

14 $$R^2 = 1 - \frac{{\mathop {\sum }\nolimits_{i = 1}^n \left( {y_i - f_i} \right)^2}}{{\mathop {\sum }\nolimits_{i = 1}^n \left( {y_i - \bar y} \right)^2}} = 1 - \frac{{\frac{1}{n}\mathop {\sum }\nolimits_{i = 1}^n \left( {y_i - f_i} \right)^2}}{{\frac{1}{n}\mathop {\sum }\nolimits_{i = 1}^n \left( {y_i - \bar y} \right)^2}} = 1 - \frac{{\rm{MSE}}}{{\delta _{\rm{DB}}^2}}.$$

15 $${\rm{RMSE}} = \sqrt {\rm{MSE}} = \sqrt {\left( {1 - R^2} \right)\delta _{\rm{DB}}^2} = \sqrt {\left( {1 - 0.5} \right) \times 337} = 13.0\,^\circ {\rm{C}}.$$

where *f*_*i*_ is the predicted value and *y*_*i*_ is the observed true value of enzyme *i*. Then each one of these predicted *T*_opt_ *s* was described with a normal distribution *N*(*T*_opt,*i*_, 13.0).

### Heat capacity change

${\Delta}C_p^\ddagger$ value was approximated by assuming temperature dependence of yeast cell growth rate as −6.3 kJ/mol/K for all enzymes^31^. Given that ${\Delta}C_p^\ddagger$ should be in the general negative for most enzymes^33^, a standard variance of 2.0 was selected from testing a wide range of values because it covers a broad range of ${\Delta}C_p^\ddagger$ and with a very low possibility of getting a positive value (Supplementary Fig. 15). A normal distribution of *N*(−6.3, 2.0) was subsequently used to describe the ${\Delta}C_p^\ddagger$ of all enzymes.

### Non-growth associated ATP maintenance

To capture the increased expenditure for maintenance under increased heat stress, an empirical equation (Supplementary Fig. 1) was constructed to estimate the NGAM at different temperatures:

16 $${\rm{NGAM}}\left( T \right) = 0.740 + \frac{{5.893}}{{1 + e^{31.920 - \left( {T - 273.15} \right)}}} + 6.12 \times 10^{ - 6} \times (T - 273.15 - 16.72)^4,$$

based on the experimental data^5^. Since the experimental data only covers the temperature range of between 5 and 40 °C, any NGAM for temperatures lower than 5 °C was set to the value at 5 °C and for those higher than 40 °C was set to the value at 40 °C. The Eq. (16) was used for the anaerobic growth data as well as for aerobic growth since no experimental data were available for this condition.

### FBA simulations with etcYeast7.6

At a given temperature, first the *k*_cat_ values and $\frac{{[E]_{N,i}}}{{[E]_{N,i} + [E]_{D,i}}}$ were calculated and integrated into the enzyme-constrained model and then the NGAM at this temperature was calculated and included in the model. For batch growth simulations, unlimited substrates were used, the same as described in ref. ^16^. The enzyme saturation factor *σ* of 0.5 was used^39^. For the simulation of anaerobic growth, in addition to the above changes, the uptake of oxygen was blocked and fatty acids and sterols were supplied into the medium as described in^16^. The growth associated with ATP maintenance (GAM) was estimated from experimental data^5^ as 70.17 mmol ATP/gdw. Other parameters were unchanged. For the simulation of fluxes at aerobic chemostat conditions, with the same model settings as aerobic batch conditions, the simulation was carried out by first fixing the growth rate to a given dilution rate (0.1 h^−1^) and minimizing the glucose uptake rate. Then the glucose uptake rate was fixed to the simulated value multiplied by a factor of 1.001 (for simulation purposes). Finally, the total enzyme usage was minimized (same as used in ref. ^16^). To get the flux sensitivity coefficient of an enzyme at a given temperature, the *k*_cat_ of all reactions associated with this enzyme were perturbed by a factor of (1 + *δ*). Then the maximal growth rates were simulated before (*u*) and after (*u*_*p*_) perturbation. Finally, the flux sensitivity coefficient of enzyme *i* was calculated as $\frac{{\frac{{u_p - \mu }}{u}}}{\delta }$, where *μ* and *u*_*p*_ are maximal specific growth rate before and after perturbation. *δ* of 10 was used in this study.

### The distance function used in SMC-ABC approach

The observed data used in this study was the maximal specific growth rate in aerobic^4^ and anaerobic^5^ batch cultivations at different temperatures, and glucose, carbon dioxide, and ethanol flux values at different temperatures measured in chemostat cultivations with a dilution rate of 0.1 h^−1^ ^23^. The distance function was designed as follows: first, the coefficient of determination (*R*^2^) between simulated and experimental data was calculated for each of the above conditions. Then the average *R*^2^ across these three conditions multiplied by −1 was used to represent the distance $\rho \left( {D,\hat D} \right)$. ${\it{\epsilon }}$ of −0.9 was used in the SMC-ABC simulation.

### Statistical tests for comparison between *P*(*θ*) and *P*(*θ*|*D*)

The significance test for the difference in mean values between *Prior* and *Posterior* was carried out by Welch’s *t* test^62^. The significance test for reduced variance was carried out by the one-tailed *F*-test. *p* values were adjusted with the correction^63^ using a family-wise error rate of 0.01. The significance cutoff was set to 0.01 (Fig. 2e).

### Machine learning applied to score the importance of parameters

Totally, 2292 parameters of 21504 parameter sets were used as the input feature matrix and the average *R*^2^ scores obtained with the Bayesian approach were used as target labels. The dataset was split into train (80%), validation (10%), and test (10%) datasets. A random forest regressor with 1000 estimators was used. The train and validation datasets were used to optimize the hyperparameter. The obtained model could explain in total 23% the variance in the test dataset. The feature importance scores were extracted directly from the obtained model.

### Genetic manipulation for the experimental validation of ERG1

The background strain we used in this study was IMX581 derived from CEN.PK113-5D, which contains an integrated Cas9 expression cassette controlled by TEFp promoter^64^. All the genetic manipulations were conducted based on the CRISPR/cas9 system. The codon-optimized kmERG1 were ordered from GenScript (Supplementary Data 1), and the PrimerSTAR HS polymerase was utilized for gene amplification through PCR. Based on strain IMX581, the codon-optimized gene ERG1 from *K. marxianus* (KmERG1) was integrated to replace the native ERG1 (ScERG1) using CRISPR/cas9, yielding HL01. All the design and construction of the plasmid follows the previously described method^64^. The gRNA cassette for target gene scERG1 was obtained using the single-stranded oligos gRNA-ERG1-F/gRNA-ERG1-R, followed by assembling with the linearized backbone plasmid pMEL10, the single gRNA plasmid was constructed by Gibson assembly. The repair fragment containing kmERG1 with round 60 bp overlap was amplified by primers kmEGR1-scERG1up-F/kmEGR1-scERG1dn-R using codon-optimized kmERG1 as a template. Then the repair fragment and single gRNA plasmid were co-transformed into IMX58. All the strains and primers used in this study were listed in Supplementary Tables 2 and 3.

### Strain cultivation under different temperatures

The thermotolerance was tested and compared between *S. cerevisiae* IMX581 and HL01. Five single colonies of each strain were selected and precultured in YPD media at 30 °C, and cells were then transferred to flasks in 20 mL YPD media to reach 0.1 initial OD600 cultured at 40 ± 0.5 °C, 200 rpm. After that, the cells were transferred into fresh YPD media every 24 h with 0.1 initial OD600 and cultivated at 40 ± 0.5 °C, 200 rpm.

### Reporting summary

Further information on research design is available in the Nature Research Reporting Summary linked to this article.

## Supplementary information

Supplementary information

Peer Review File

Reporting Summary

Description of Additional Supplementary Files

Supplementary Data 1

## Data Availability

Data supporting the findings of this work are available within the paper and its Supplementary Information files. A reporting summary for this article is available as a Supplementary Information file. The datasets and plant materials generated and analyzed during the current study are available from the corresponding author upon request. The data for reproducing the figures in both the main and supplementary files are provided as a Zenodo repository (https://zenodo.org/record/3996543#.X0J1BNP7S3I). Source data are provided with this paper.

## Source data

Source Data

## Code availability

All simulations of genome-scale models have carried out with Cobrapy^64^ with Gurobi (Gurobi Optimization, LLC) solver. All code is available on Github (https://github.com/SysBioChalmers/BayesianGEM).
