# Supplementary material for: Bayesian genome scale modelling identifies thermal determinants of yeast metabolism
Source: Nat Commun. 2021 Jan 8;12:190. doi: 10.1038/s41467-020-20338-2 (PMC7794507; doi:10.1038/s41467-020-20338-2)
Supplement: Supplementary file 1 — Supplementary information [file 41467_2020_20338_MOESM1_ESM.docx]

Bayesian genome scale modelling identifies thermal determinants of yeast metabolism

Li et al.

## Supplementary Note 1: Validation of SMC-ABC approach with toy models

To evaluate the performance of the SMC-ABC approach proposed in this work, several toy models with known parameters, including linear models and ordinary differential equations (ODEs) were used. Several other methods, including least squares fitting, genetic algorithm and classical Sequential Monte Carlo based Approximate Bayesian Computation [^1,2^](https://paperpile.com/c/9r1FLj/ttp7D+r6TXp) were also tested and compared to our approach.

**Least squares fitting**. Levenberg-Marquardt algorithm was used in this work. **scipy.optimize.leastsq** function from the python package scipy [^3^](https://paperpile.com/c/9r1FLj/3Vqn2) was used.

#### **Genetic Algorithm** For a sample described with $m$parameters ($[x_{1},x_{2},...,x_{m} ]$), each parameter was considered as one gene for this sample (an individual). The crossover between two individuals (call them father and mother) was designed as follows: for each gene of the child, there is equal probability (50%) that it is from its father or mother. Each gene in the child has a mutation probability of $p_{i}$. The mutation was done by adding a random value sampled from a normal distribution $N(0,\sigma_{i}^{2})$. The negative mean squared error (MSE) between simulated data and observed experimental data was used as the fitness score. Each individual in the population has a different probability of being selected as a parent for crossover. This probability was designed as follows: convert all fitness scores to non-negative by subtracting the minimal fitness score and then divide by the summation of all these converted scores. The resulting scores have a summation of 1 and thus were used as weights for parents sampling.

#### **Classical SMC-ABC.** The approach was done by using the python package pyabc [^4^](https://paperpile.com/c/9r1FLj/NoBCh). Multivariate normal transitions were used. The median epsilon of the last population was used for the next iteration.

For all population-based methods (GA, classical SMC-ABC and SMC-ABC in this work), a population size of 100 was used. Same stop criteria and initial parameter distribution were applied. In all cases, the stop criterion was designed as the median MSE of simulated results in the last population is less than the mean squared error between calculated true $y$ values and ones after adding noise.

Several toy models were tested. In the first case, a *p<N* problem where the number of variables is smaller than the number of samples was designed. A simple linear model was used: $y=2x_{1}+3x_{2}$. 100 samples were generated by randomly sampling a list of $\boldsymbol{x}=[x_{1},x_{2}]$, in which $x_{1}$ and $x_{2}$ were randomly sampled from a standard normal distribution. Gaussian noise of $N(0,2^{2})$was added to the calculated $y$. The initial guess of [5,5] was used for the least square method and the normal distribution $N(5,3^{2})$ for both coefficients for other population-based methods. All fitting algorithms including the SMC-ABC approach proposed in this work can converge to the desired precision (Supplementary Figures 16a-c). All three population-based algorithms showed similar performance in estimating two coefficients ($\beta_{1}$and $\beta_{2}$) (Supplementary Figure 16d).

In the second case, a nonlinear model $\boldsymbol{y}=asin{(x}_{1})+bcos(x_{2})+c$ was tested. 100 samples were generated by randomly sampling a list of $\boldsymbol{x}=[x_{1},x_{2}]$, in which $x_{1}$and $x_{2}$were randomly sampled from a standard normal distribution. Gaussian noise of $N(0,2^{2})$was added to the calculated $y$. The true parameter values were assumed as $a=2,b=3,c=1$. The initial guess of [5, 5, 5] for [*a, b, c*] was used for least square method and the normal distribution $N(5,3^{2})$ for all parameters for other population-based methods. All fitting algorithms including the SMC-ABC approach proposed in this work can converge to the desired precision (Supplementary Figures 17a-c). The SMC-ABC approach proposed in this work showed similar performance to the classic SMC-ABC approach. The GA approach achieved different distributions of estimated parameters (Supplementary Figure 17d).

In the third case, a *p>N* problem (with relatively small *p*) where the number of variables is greater than the number of samples was designed. A linear model $y=\sum_{i=1}^{20} a_{i}x_{i}$ with 20 parameters was used. The true values of those coefficients were randomly sampled from a uniform distribution $U(0, 10)$. 5 samples were generated by randomly sampling a list of $\boldsymbol{x}=[x_{1},x_{2},...,x_{20}],$ of which each $x_{i}$was randomly sampled from a standard normal distribution. Gaussian noise of $N(0,2^{2})$ was added to each sample. The normal distribution $N(5,3^{2})$ was used as an initial guess (*prior*) for all coefficients $a_{i}$. All fitting algorithms including the SMC-ABC approach proposed in this work can converge to the desired precision (Supplementary Figures 18a-c). The SMC-ABC approach proposed in this work showed the best estimation of true parameters with the smallest MSE value between true parameter values and mean values of 100 *Posterior* parameter sets. Since this is a *p>N* problem, the resulting estimated parameters should not converge to a single solution. As shown in Supplementary Figure 18d, the estimated parameters by GA approach tends to converge to a single solution, with a lower parameter variance compared to the SMC-ABC approaches (Supplementary Figures 18ef). We demonstrate a better description of *Posterior* estimation of parameters with the SMC-ABC approach proposed in this work (Supplementary Figure 18f), as it showed a bigger feasible *Posterior* parameter space compared to the classical one (Supplementary Figure 18e).

In the fourth case, a *p>>N* problem (with big *p*) where the number of variables is greater than the number of samples was designed. A linear model $y=\sum_{i=1}^{100} a_{i}x_{i}$ with 100 parameters was used. The true values of those coefficients were randomly sampled from a uniform distribution $U(0, 10)$. 10 samples were generated by randomly sampling a list of $\boldsymbol{x}=[x_{1},x_{2},...,x_{100}]$ of which each $x_{i}$was randomly sampled from a standard normal distribution. Gaussian noise of $N(0,{10}^{2})$ was added to each sample. The normal distribution $N(5,3^{2})$ was used as an initial guess (*prior*) for all coefficients $a_{i}$. The GA algorithm and the SMC-ABC approach proposed in this work converge to the desired precisions (Supplementary Figures 19ab). The classic SMC-ABC failed in this case as it is very slow in sampling when the parameter dimension is high [^5^](https://paperpile.com/c/9r1FLj/iXIWM). The SMC-ABC approach proposed in this work showed a better parameter estimation than GA in terms of MSE between true parameter and mean values of 100 final parameter sets (Supplementary Figure 19). Furthermore, GA tends to overfit the model as estimated parameters showed a very low variance and tends to converge to a single solution in the parameter space (Supplementary Figure 19c). We demonstrate a better description of *Posterior* estimation of parameters with the SMC-ABC in this work (Supplementary Figure 19d), as it showed a bigger feasible *Posterior* parameter space compared to GA, and thereby avoids overfitting (Supplementary Figure 19c).

In the last case, the repressilator model [^6^](https://paperpile.com/c/9r1FLj/2npsh), which is an ODE toy model for a three-gene transcriptional regulation system that consists of four parameters to describe the dynamic behaviour. In the regulatory feedback loop, each gene produces the repressor protein for the next gene. Mathematical description is as follows:

$\frac{dm_{1}}{dt}=-m_{1}+\frac{\alpha}{1+p_{3}^{n}}+\alpha_{0}$ (1)

$\frac{dp_{1}}{dt}=-\beta\left( p_{1}-m_{1} \right)$ (2)

$\frac{dm_{2}}{dt}=-m_{2}+\frac{\alpha}{1+p_{1}^{n}}+\alpha_{0}$ (3)

$\frac{dp_{2}}{dt}=-\beta\left( p_{2}-m_{2} \right)$ (4)

$\frac{dm_{3}}{dt}=-m_{3}+\frac{\alpha}{1+p_{2}^{n}}+\alpha_{0}$ (5)

$\frac{dp_{3}}{dt}=-\beta\left( p_{3}-m_{3} \right)$ (6)

where the $p_{1}, p_{2}, p_{3}$are three repressor-protein concentrations, $m_{1}, m_{2}, m_{3}$are their corresponding mRNA concentrations. $\theta(\alpha_{0},n,\beta,\alpha)$ are the parameter vector where$\alpha_{0}$ is the number of protein copies of per cell in the presence of saturating amounts of repressor; $\alpha$is the increment when repressor absence;$\beta$ is the ratio of the protein decay rate to the mRNA decay rate; $n$is a Hill coefficient; $t$ is the lifetime of units of the mRNA. Default conditions for simulation were taken from [^1^](https://paperpile.com/c/9r1FLj/ttp7D): $\theta\left( \alpha_{0}, n, \beta, \alpha\right)=(1, 2, 5, 1000)$; initial conditions are ($m_{1},m_{2},m_{3}, p_{1},p_{2},p_{3})_{t0}=(0, 0, 0, 2, 1, 3)$; and add Gaussian noise as $N(0,5^{2})$ to get the experiment data. The model assumed that only the mRNA ($m_{1},m_{2},m_{3})$ concentrations are available, and the protein concentrations are considered as missing data as described in the above reference. Thereby, only data for $m_{1},m_{2},m_{3}$ were used to calculate the MSE. In this case, GA and SMC-ABC proposed in this work failed to converge to the desired precision after 200 iterations (desired MSE: 24.97; GA: 55.18; SMC-ABC this work: 25.86) (Supplementary Figures 20ac). The classic SMC-ABC converges very fast to the desired precision (Fig S20b). Only the classic SMC-ABC approach gives a good estimation of *Posterior* estimation of parameter values while GA and SMC-ABC approach proposed in this work failed. Particularly, the SMC-ABC approach converged to a single solution where all sampled 100 *Posterior* parameter sets are identical and differ from the true values (Supplementary Figure 20d). This is due to the covariance between different parameters should be considered in the *Posterior* (Supplementary Figure 20e). The SMC-ABC approach proposed in this work assumed zero covariance between parameters and thus failed for such cases where parameters are correlated in the true *Posterior*.

From the above simulations we can conclude that

1. GA, classic SMC-ABC and the one proposed in this work showed similar performance when the model is simple, and the number of parameters is smaller than that of samples (*p<N*) (Supplementary Figures 16&17);
2. In case of *p>N,* The SMC-ABC proposed in this work showed the best performance and can make the mode less overfitted by showing a larger feasible parameter space (Supplementary Figures 18&19); GA and classic SMC-ABC tends to overfit the model. The classic SMC-ABC fails when the number of parameters is high (~100).
3. In case of correlated parameters in the true *Posterior*, the SMC-ABC proposed in this work can fail as it ignores the covariance between parameters (Supplementary Figure 20).

Thereby, in the case of etcYeast model where there are 2,292 parameters to be estimated, the SMC-ABC proposed in this work is the best option among the other methods tested here.

###

## Supplementary Figures


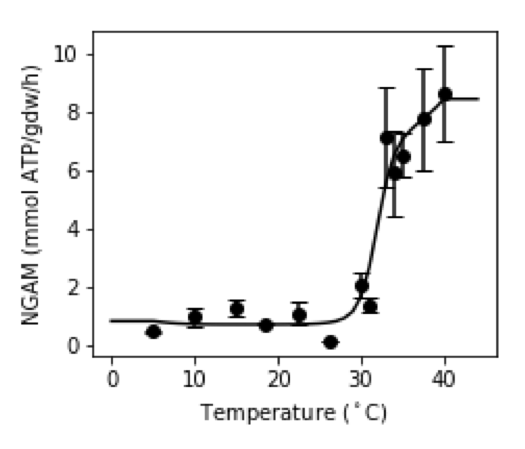


**Supplementary Figure** **1**. Fit an empirical equation to describe the temperature dependence of non-growth associated ATP maintenance (NAGM). The experimental data was collected from Zakhartsev M. *et al.* [^7^](https://paperpile.com/c/9r1FLj/WEnj). The line represents the fitted curve: $NGAM\left( T \right)=0.740+\frac{5.893}{1+e^{31.920-\left( T-273.15 \right)}}+6.12\times{10}^{-6}{\times(T-273.15-16.72)}^{4}$. The values and error bars were collected from the original paper, which were given ﻿as average$\pm$max/min (n=2).


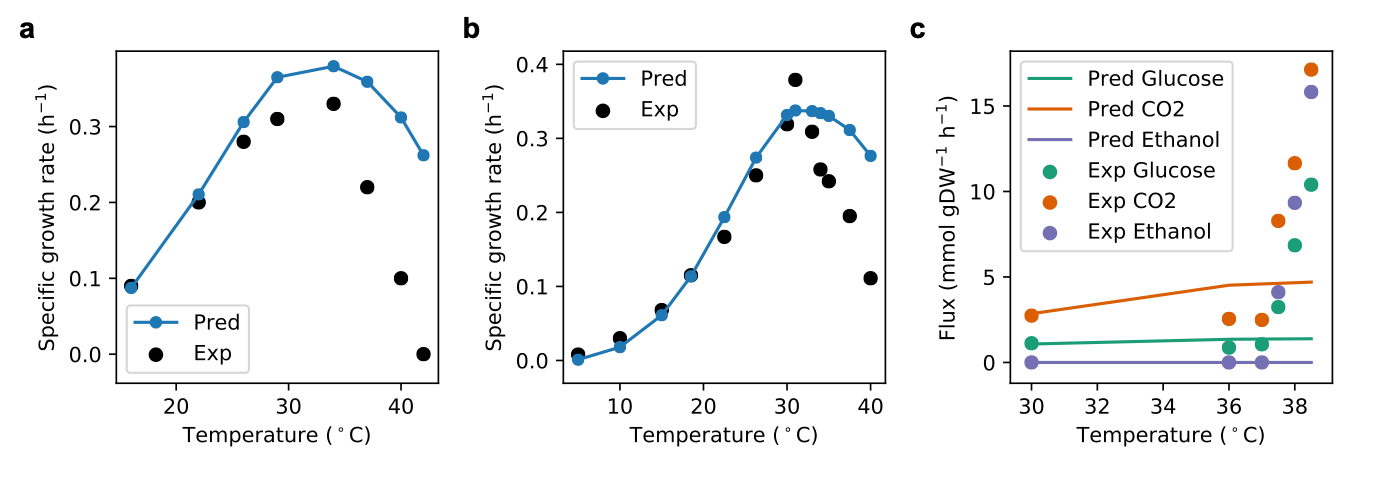


**Supplementary Figure** **2**. Simulated results of specific growth rate at (a) aerobic, (b) anaerobic batch cultivations and (c) fluxes at chemostat cultivation, with the model equipped with initial parameters as described in Methods M5. The black dots are experimental data collected from Caspeta L. *et al*. [^8^](https://paperpile.com/c/9r1FLj/oB6s) for (a), Zakhartsev M. *et al*. [^7^](https://paperpile.com/c/9r1FLj/WEnj) for (b) and Postmus J. *et al*. [^9^](https://paperpile.com/c/9r1FLj/0T9i) for (c). Pred, predicted. Exp, experimental.


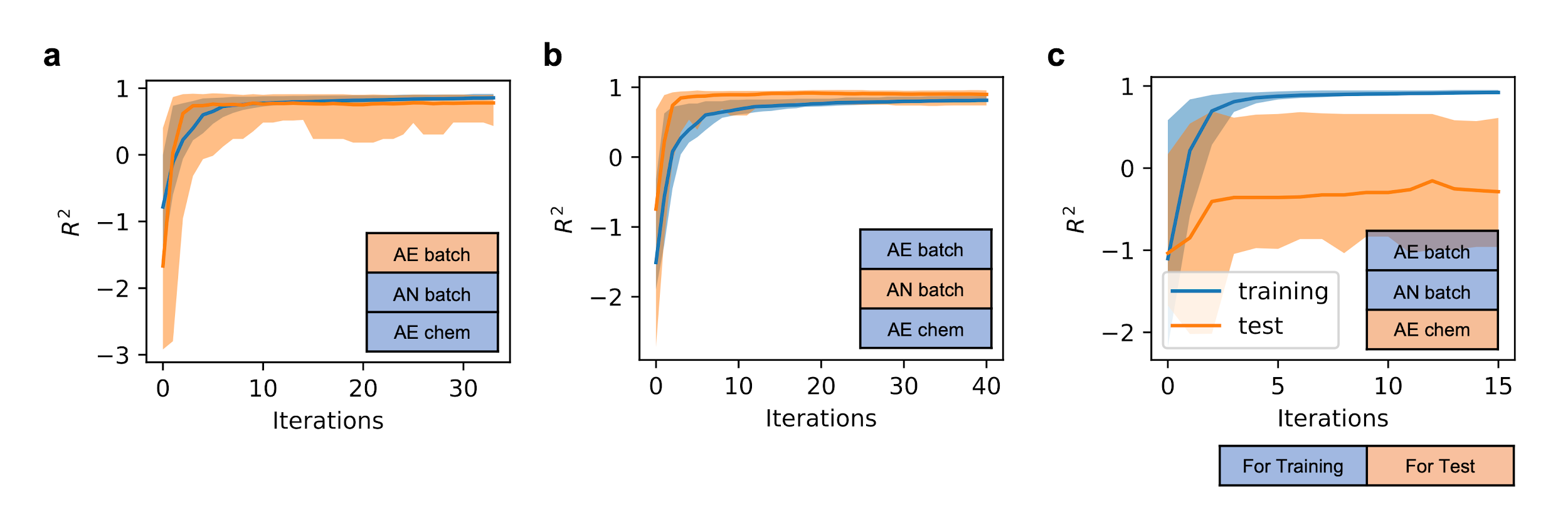


**Supplementary Figure 3**. Three-fold cross-validation of SMC-ABC approach. Figure legends: AE batch and AN batch are the specific growth rate datasets in aerobic [^10^](https://paperpile.com/c/9r1FLj/dBST) and anaerobic [^7^](https://paperpile.com/c/9r1FLj/WEnj) batch cultivations, respectively, and AE chem are the experimental flux measurements in aerobic chemostat cultivation [^9^](https://paperpile.com/c/9r1FLj/0T9i). For each fold, the two datasets (blue) were used to update the *Prior* model and were then tested on the remaining dataset (orange) during iterations. All the *Posterior* models showed an improved performance on the test dataset through iterations. Whereas *Posterior* models trained on both batch and chemostat data showed good performance on the rest of the batch data (aerobic or anaerobic, Supplementary Figures 3ab), ones trained on batch data (aerobic and anaerobic growth rate) showed poor performance on chemostat data (Supplementary Figure 3c: *R^2^*_test_ < 0). These findings indicated it is necessary to use all datasets to update the *Prior,* especially since batch and chemostat data provide non-overlapping, orthogonal information. In a-c, lines indicate median values and shaded areas indicate regions between the 5-th and 95-th percentiles (n=100).


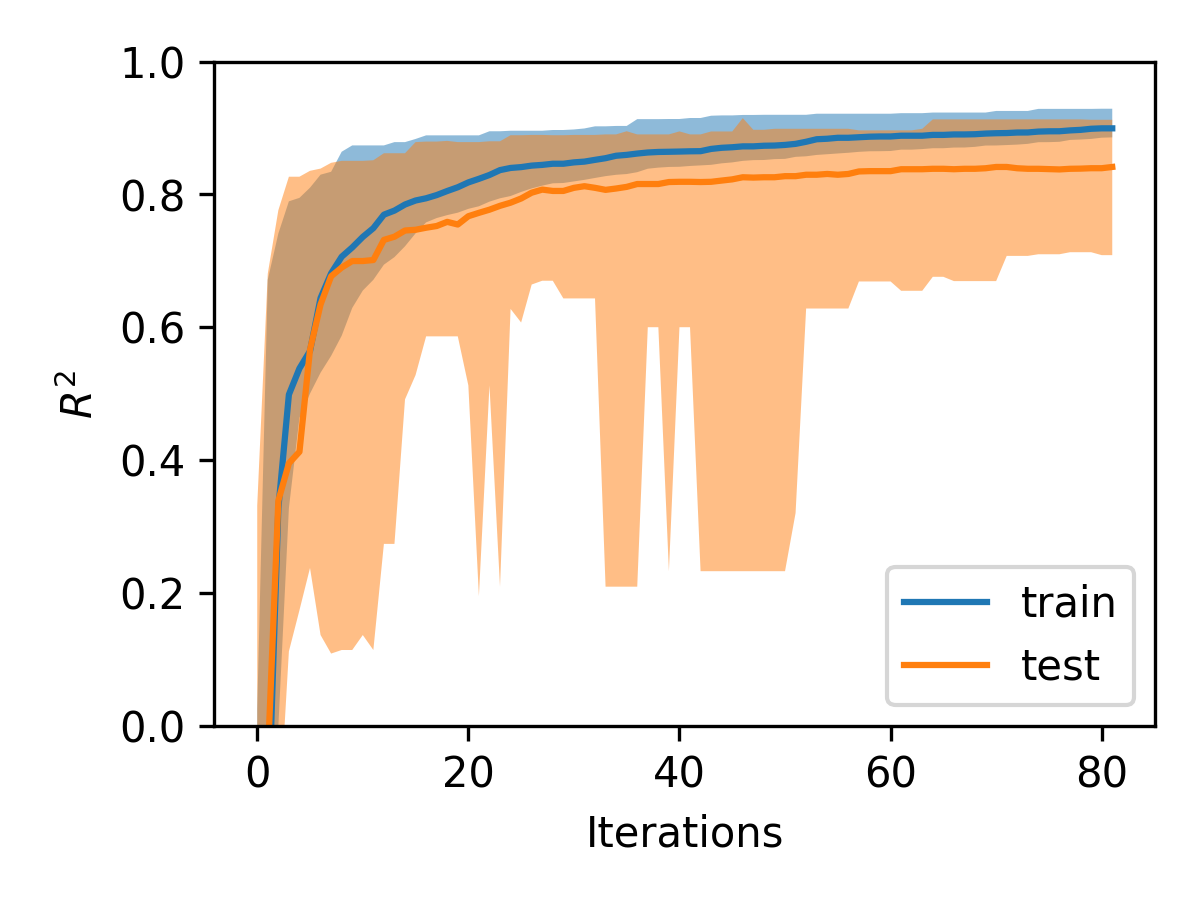


**Supplementary Figure** **4**. Validation of SMC-ABC approach. In this validation approach, data at 50% of temperature points were used to update the *Prior* model and then tested on the remaining 50%. All three datasets as used in Supplementary Figure 2 were used. For each dataset, the split was done by first sorting all the data based on temperatures, then choosing the ones with even index (starts from 0) for training and others for test. Temperature points for training: aerobic batch, 16.0, 26.0, 34.0, 40.0 °C; anaerobic batch: 5.0, 26.3, 33.0, 37.5 °C; chemostat: 30.0, 37.0, 38.0 °C. Temperature points for test: aerobic batch: 22.0, 29.0, 37.0, 42.0 °C; anaerobic batch: 15.0, 30.0, 35.0, 40.0 °C; chemostat, 36.0, 37.5, 38.5 °C. Then the training and test datasets from those three datasets were combined. *R*^2^ was calculated as described in Method M6. Lines indicate median values and shaded areas indicate regions between the 5-th and 95-th percentiles (n=100).


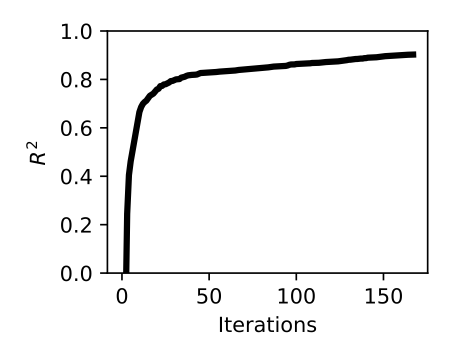


**Supplementary Figure** **5**. The average *R*^2^ score on three datasets during iterations in the Sequential Monte Carlo based Approximate Bayesian Computation (SMC-ABC) approach.


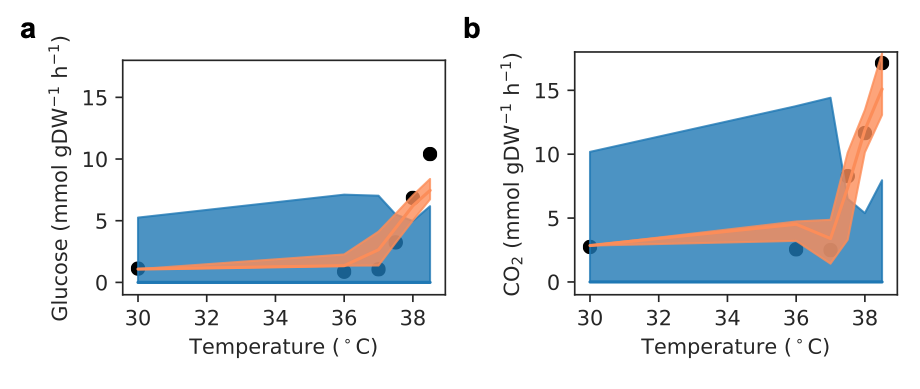


**Supplementary Figure** **6**. Simulated (a) glucose uptake and (b) CO_2_ secretion fluxes at various temperatures with *Posterior* models. Lines indicate median values and shaded areas indicate regions between the 5-th and 95-th percentiles (n=128 for *Prior* and n=100 for *Posterior* models). Black dots show the experimental values from Postmus J. *et al*. [^9^](https://paperpile.com/c/9r1FLj/0T9i) Blue and orange denote the predictions from *Prior* and *Posterior* models, respectively.


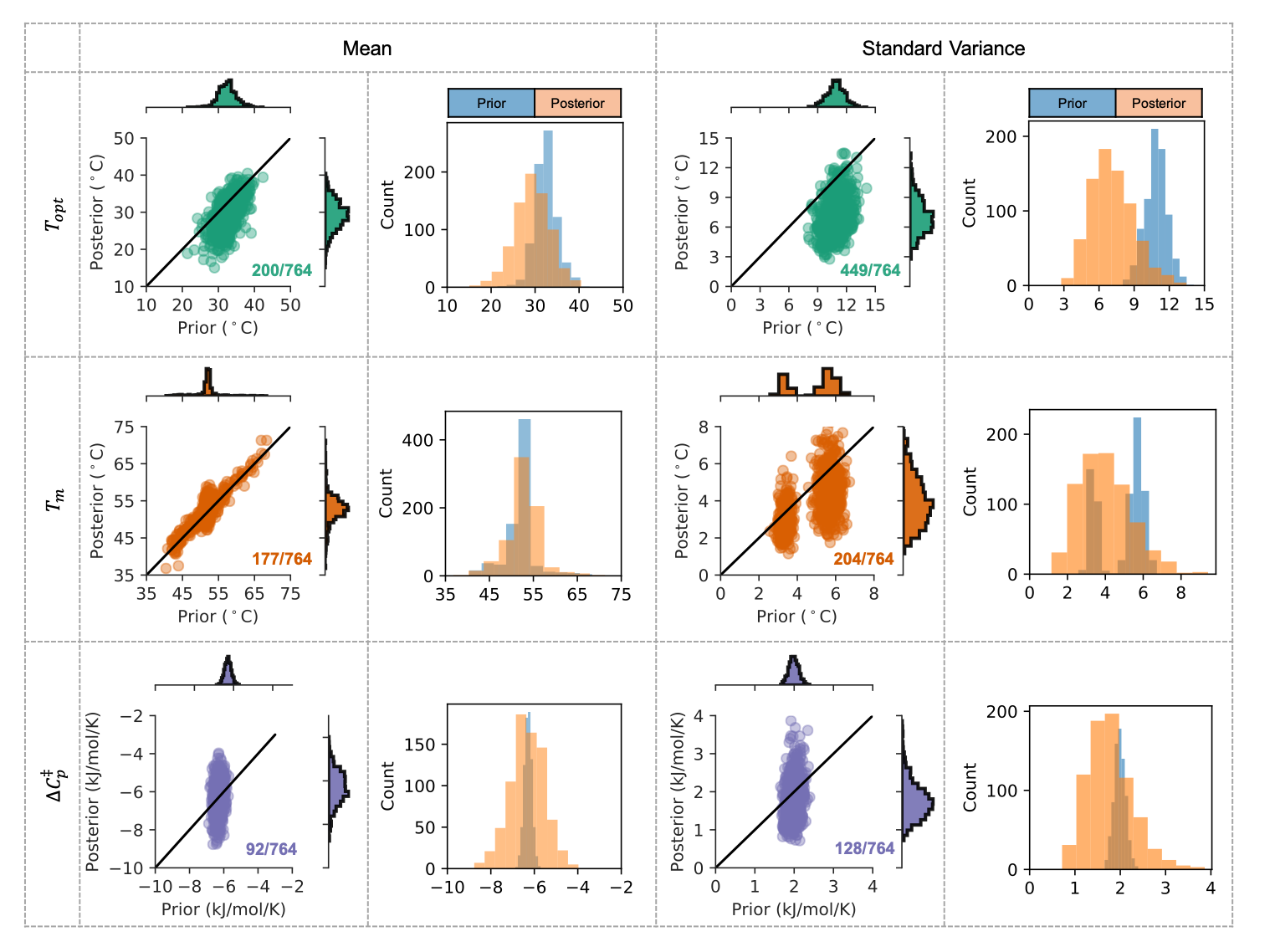


**Supplementary Figure** **7**. Parity plots and histograms for comparing mean and standard variance of $T_{opt}$s, $T_{m}$s and $\Delta C_{p}^{\ddagger}$s in the *Prior* and *Posterior* models (n=764 enzymes). The mean and standard variance are calculated based on 128 *Prior* and 100 *Posterior* models. The inset numbers indicate the number of enzymes, out of all 764, with a significantly changed mean (Šidák adj. Welch's *t*-test *p*-value < 0.01, two-sided) and variance (Šidák adj. one-tailed *F*-test *p*-value < 0.01). Source data are provided as a Source Data file.


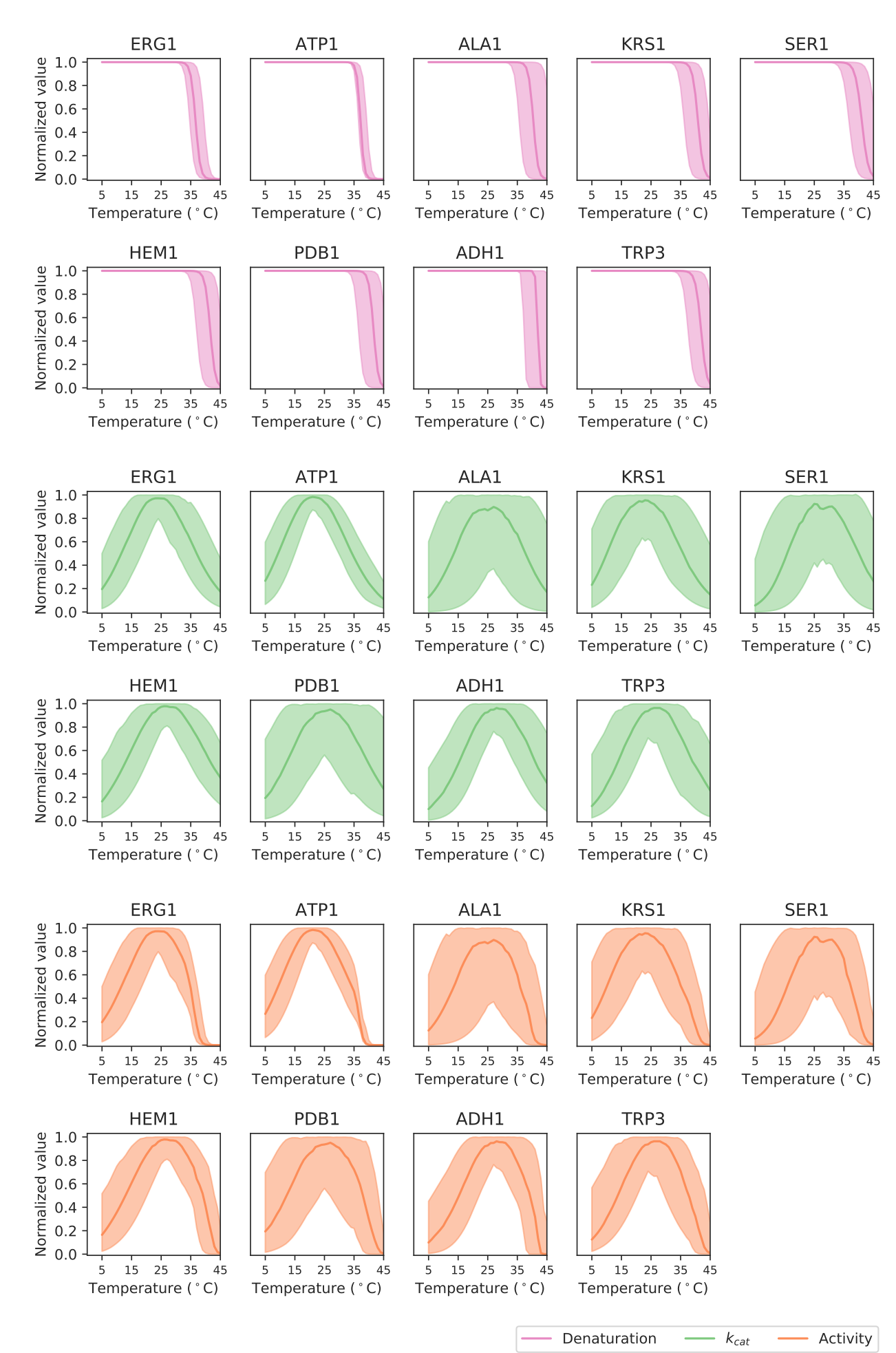


**Supplementary Figure** **8**. The temperature effect on 9 enzymes with a mean melting temperature below 42 °C in the 100 *Posterior* models. The denaturation is shown as the probability of an enzyme in the native state. The kcats is shown as normalized values by the maximal kcat. Activity, the specific activity of an enzyme is shown as the product of values in denaturation plots and kcat plots. Lines indicate median values and shaded areas indicate regions between the 5-th and 95-th percentiles (n=100).


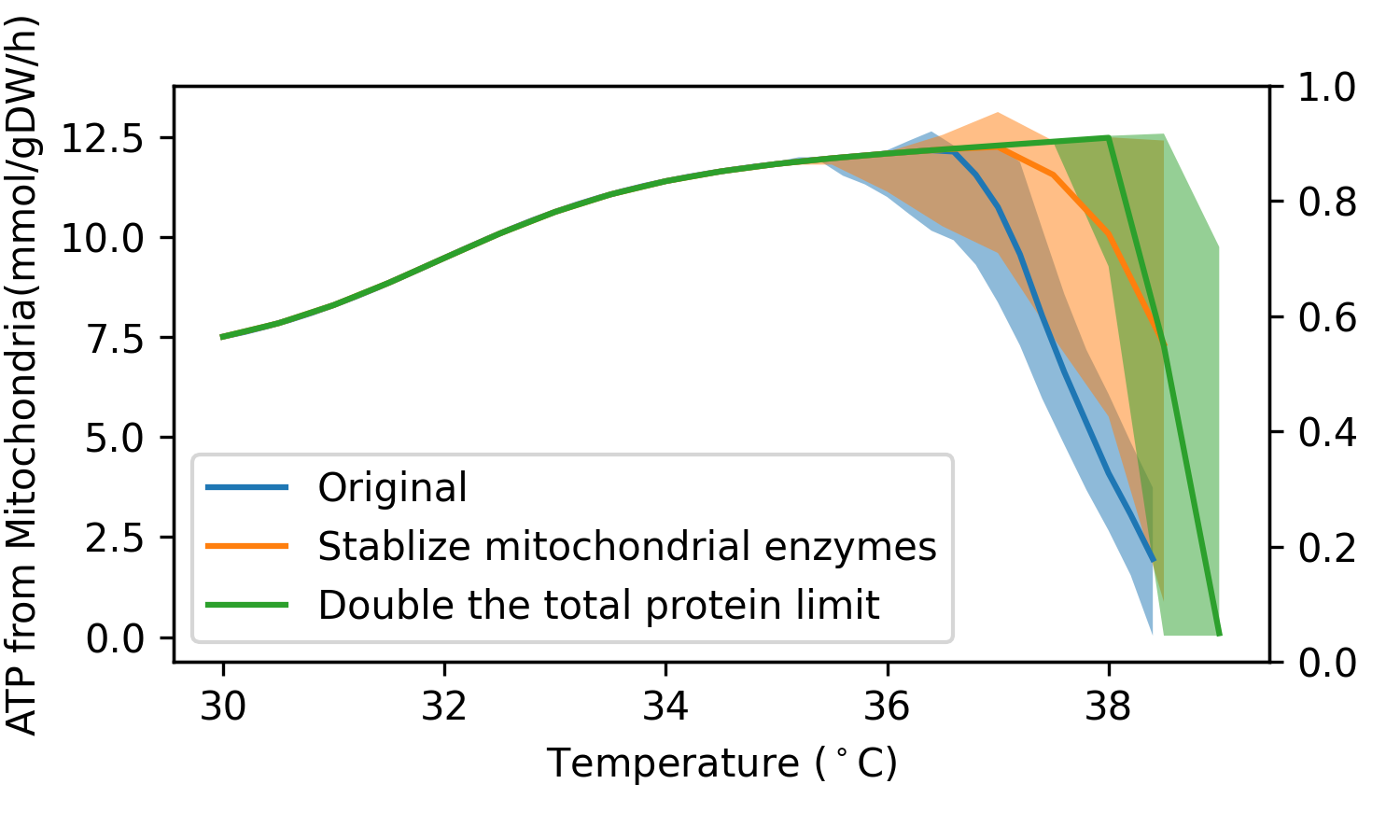


**Supplementary Figure** **9.** Comparison of ATP production from mitochondria with three model settings: (1) original *Posterior* models (correspond to Fig 4a), (2) original *Posterior* models with doubled total protein limit (Fig 4b) and (3) original *Posterior* models with three stabilized mitochondrial enzymes (ATP1, HEM1 and PDB1) (Fig 4c), at different temperatures. Lines indicate median values and shaded areas indicate regions between the 5-th and 95-th percentiles (n=100).


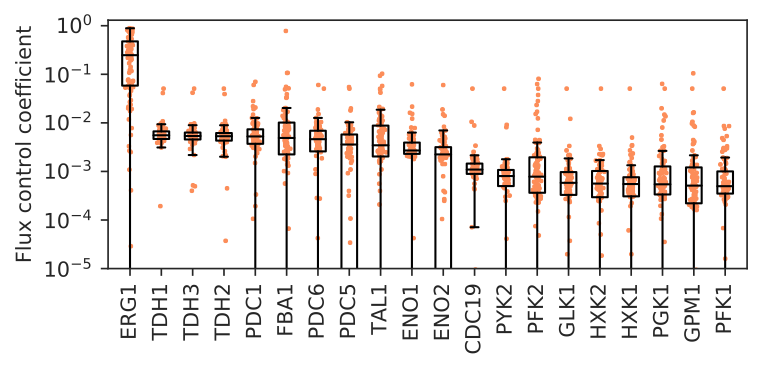


**Supplementary Figure** **10**. 20 enzymes with the highest flux sensitivity coefficients at 42 °C (n=100 for each enzyme). Center line, median; box limits, upper and lower quartiles; whiskers, 1.5x interquartile range.


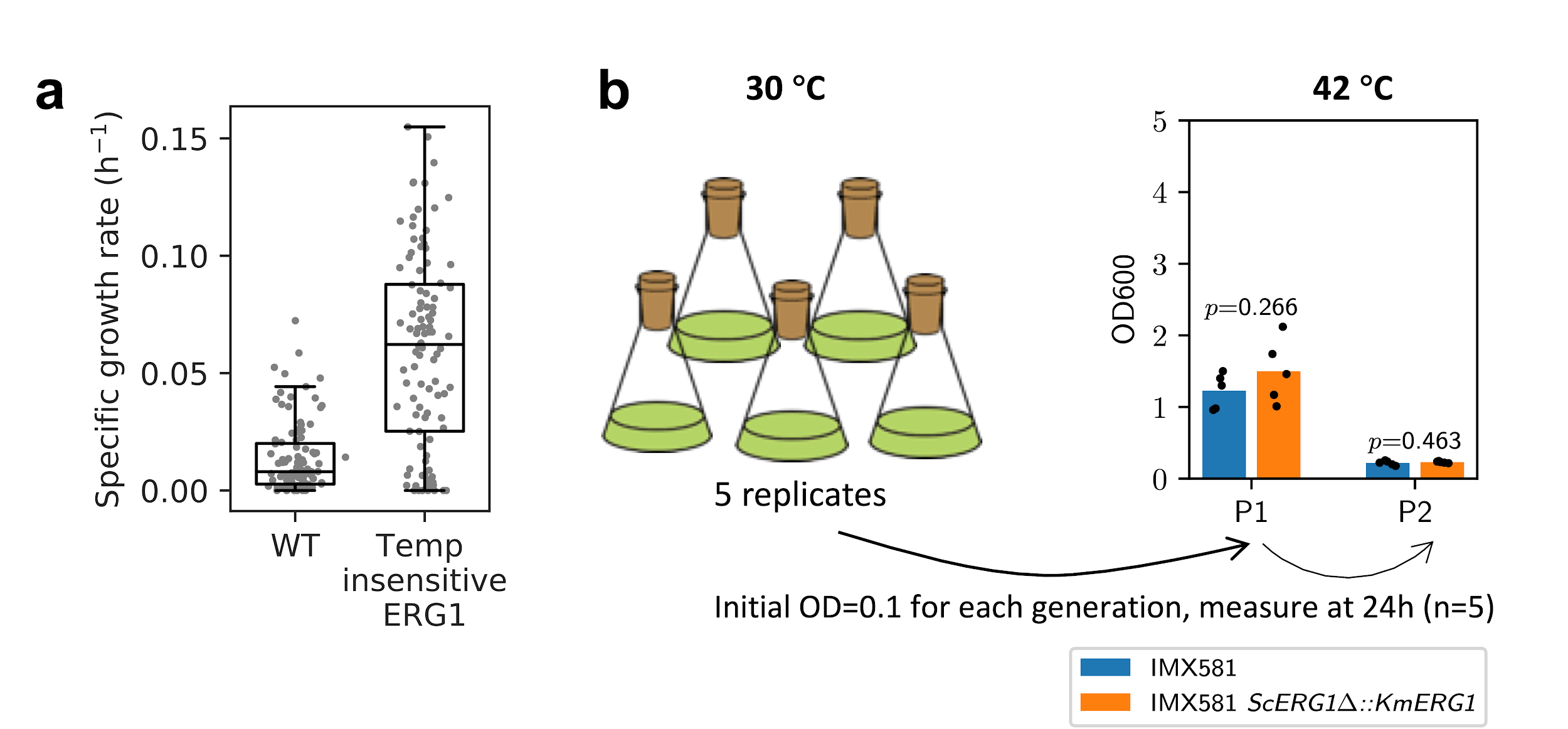


**Supplementary Figure** **11**. ERG1 at 42 °C. (a) Predicted maximal specific growth rate of wild-type yeast and the one without any temperature constraints (fully functional) on ERG1 enzyme at 42 °C (n=100 for each strain). Center line, median; box limits, upper and lower quartiles; whiskers, 1.5x interquartile range. (b) The effect of KmERG1 expression on thermo tolerance in *S. cerevisiae*. The strains were cultivated at 42 °C for two passages to reach the steady stage of growth. Optical densities (600 nm) are shown at 24 h. Data are represented as mean values of 5 replicates in the bar chart. Dots represent the values of 5 replicates. *p*-values denote Welch's *t*-test (two-sided). Source data are provided as a Source Data file.

###


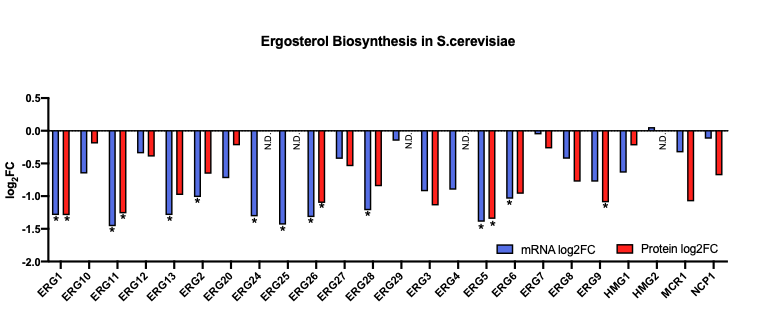


**Supplementary Figure** **12**. Downregulated ergosterol biosynthesis pathway at both transcription and translation levels when increasing the temperature from 30 °C to 36 °C. The proteomics and transcriptomics data were from Doughty T. *et al* [*^11^*](https://paperpile.com/c/9r1FLj/A49d). * indicates the genes with an absolute log2FC>1 and FDR<0.01.


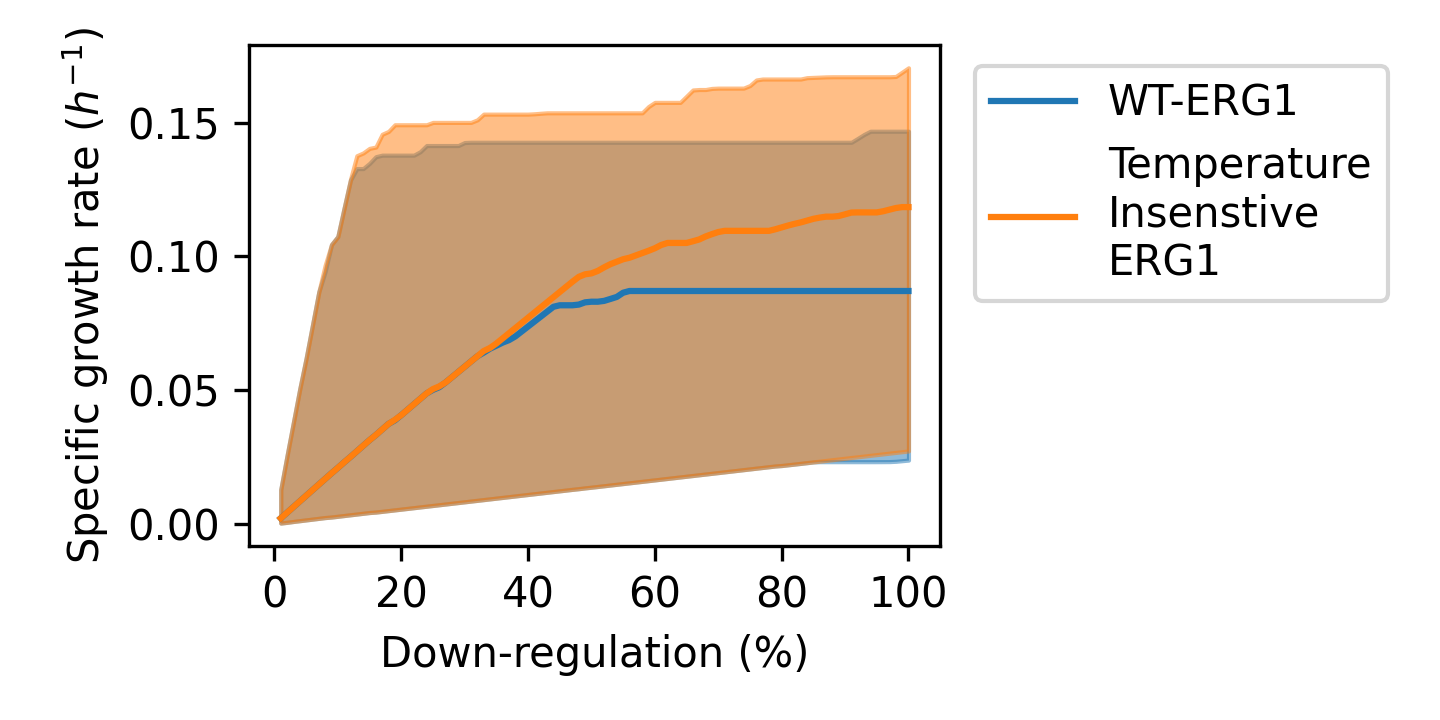


**Supplementary Figure** **13**. Predicted maximal specific growth rate of wild-type yeast and the one without any temperature constraints (fully functional) on ERG1 enzyme at 40 °C when down-regulating other enzymes involved in the ergosterol pathway (ERG10, ERG13, HMG1, HMG2, ERG12, ERG8, MVD1, IDI1, ERG20, ERG9, ERG7, ERG11, ERG24, ERG25, ERG26, ERG27, ERG6, ERG2, ERG3, ERG5 and ERG4. This is the same list as used in Supplementary Figure 12). The simulation was done by firstly identifying the maximal flux (*V*_max_) through reactions catalyzed by those enzymes with flux variability analysis, then reducing the upper bound of those reactions by multiplying a percentage value. For example, 50% of down-regulation means that set the upper bound of those reactions as 50%*V*_max_.


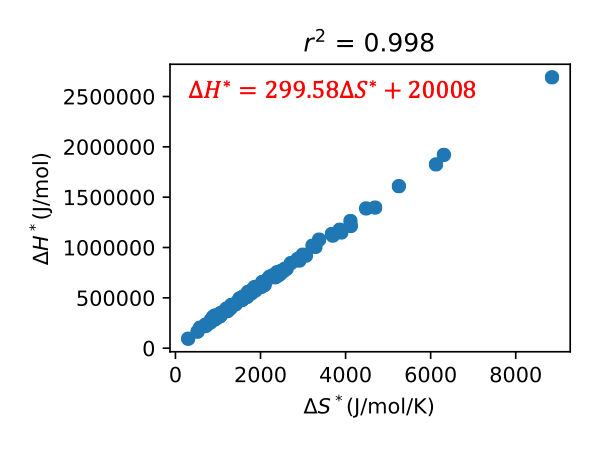


**Supplementary Figure** **14**. The correlation between enthalpy and entropy changes of protein denaturation process at convergence temperatures (373.5 K for $\Delta H^{*}$ and 385 K for $\Delta S^{*}$). The plot shows data of 116 proteins from Sawle L et al [^12^](https://paperpile.com/c/9r1FLj/6bBf).


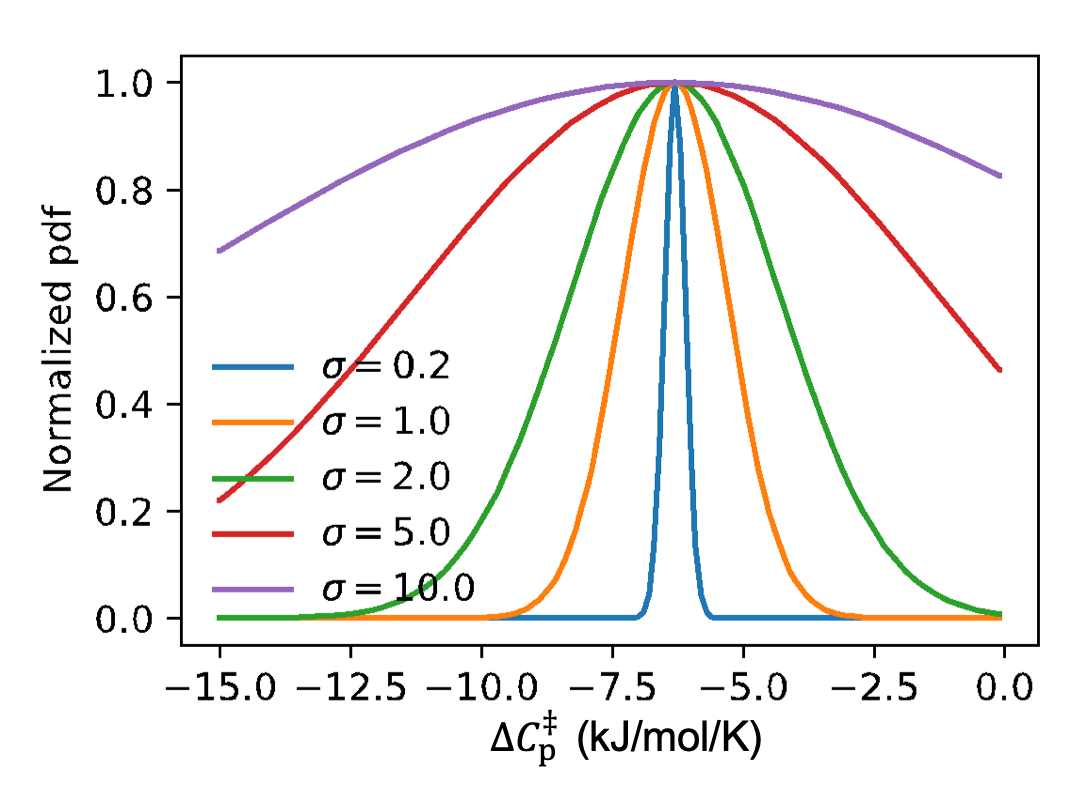


**Supplementary Figure** **15**. The normal distributions of $\Delta C_{p}^{\ddagger}$with a mean of -6.3 kJ/mol/K and different standard variances.


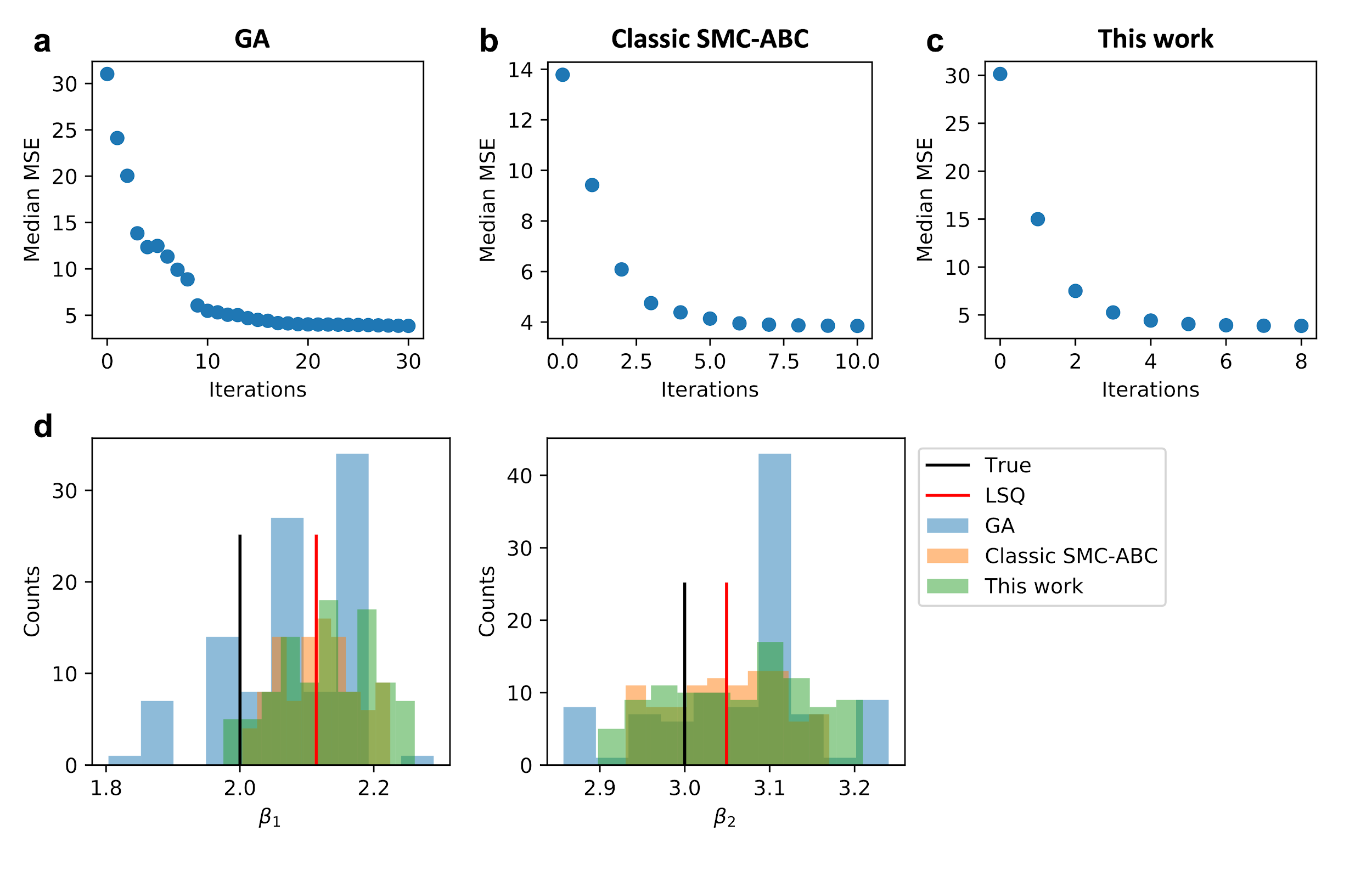


**Supplementary Figure** **16**. Validation results on the linear model $y={\beta_{1}x}_{1}+\beta_{2}x_{2}$. (a-c) Median MSE values during iterations when using (a) genetic algorithm, (b) classical SMC-ABC and (c) SMC-ABC approach proposed in this work. (d) Distributions of inferred parameter values for two coefficients in the linear model. True, the true parameter values ($\beta_{1}=2, \beta_{2}=3$); LSQ, least square fitting; GA, genetic algorithm.


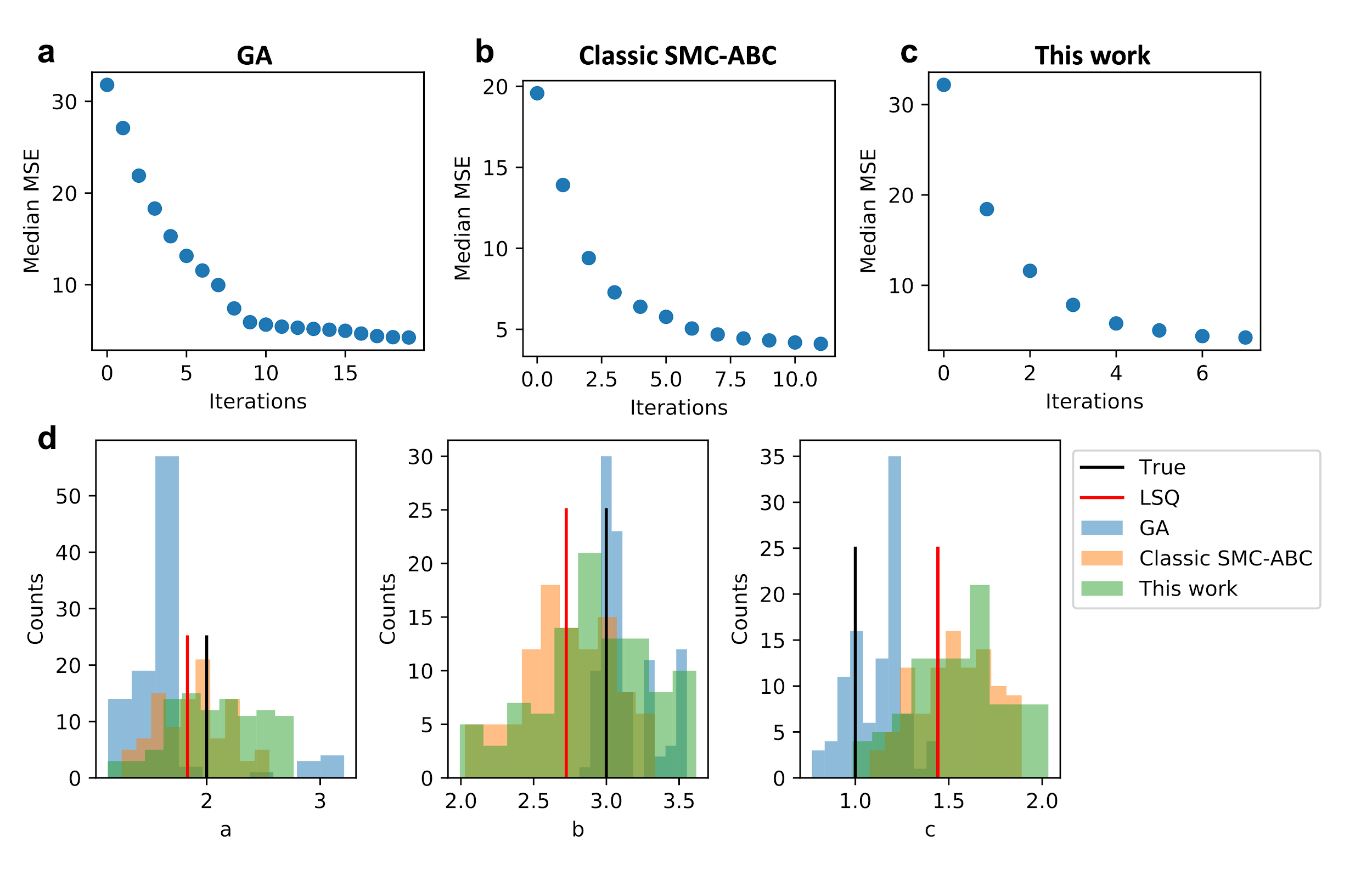


**Supplementary Figure** **17**. Validation results on the nonlinear model $y=asin{(x}_{1})+bcos(x_{2})+c$. (a-c) Median MSE values during iterations when using (a) genetic algorithm, (b) classical SMC-ABC and (c) SMC-ABC approach proposed in this work. (d) Distributions of inferred parameter values. True, the true parameter values ($a=2,b=3,c=1$); LSQ, least square fitting; GA, genetic algorithm.


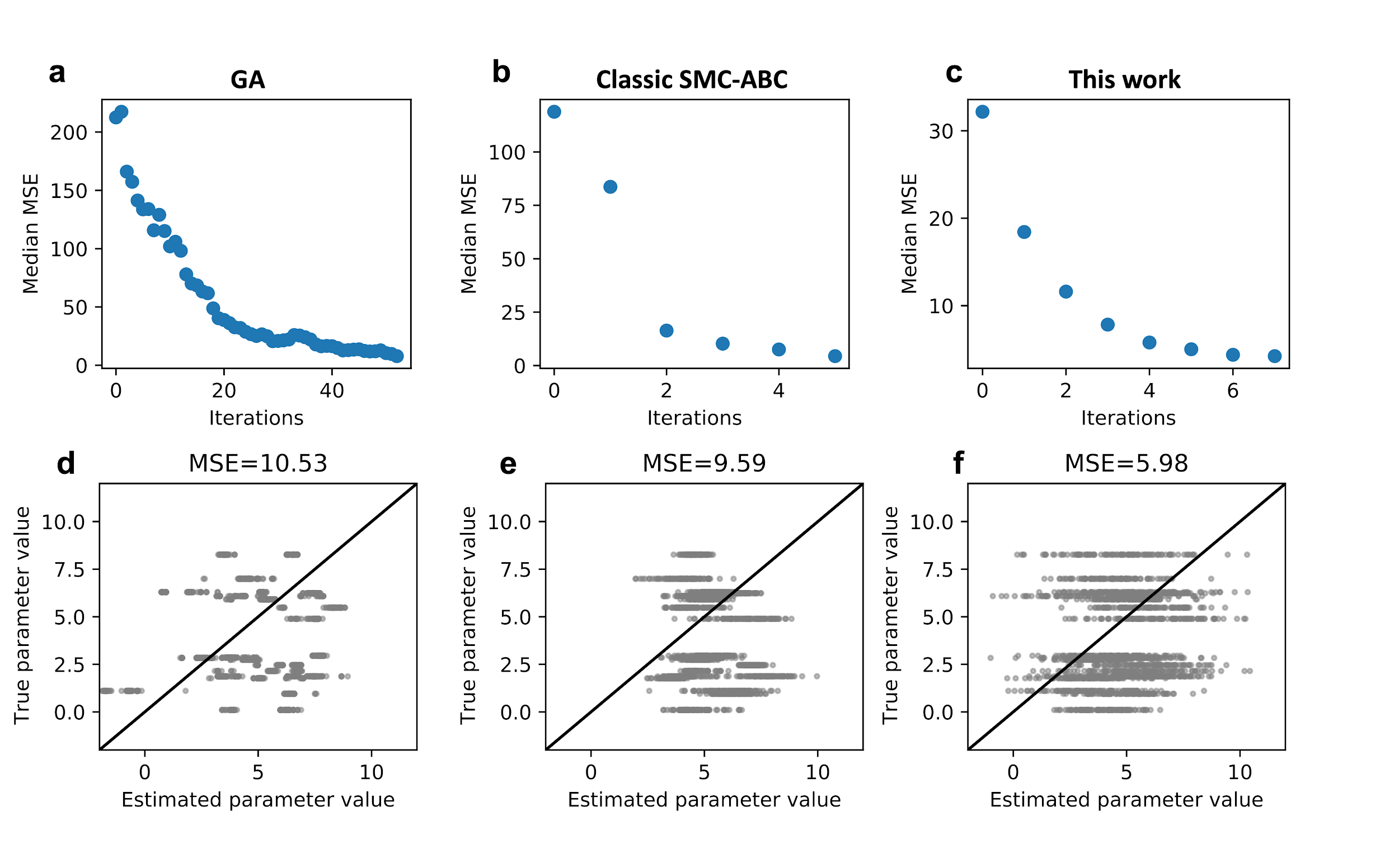


**Supplementary Figure** **18**. Validation results on the linear model $y=\sum_{i=1}^{20} a_{i}x_{i}$. (a-c) Median MSE values during iterations when using (a) genetic algorithm, (b) classical SMC-ABC and (c) SMC-ABC approach proposed in this work. (d-f) Parity plot for comparison between true parameter values and 100 sets of estimated parameters when using (d) GA, (e) classical SMC-ABC and (f) SMC-ABC approach proposed in this work. Mean squared error between true parameter values and mean values of 100 parameter sets are shown.


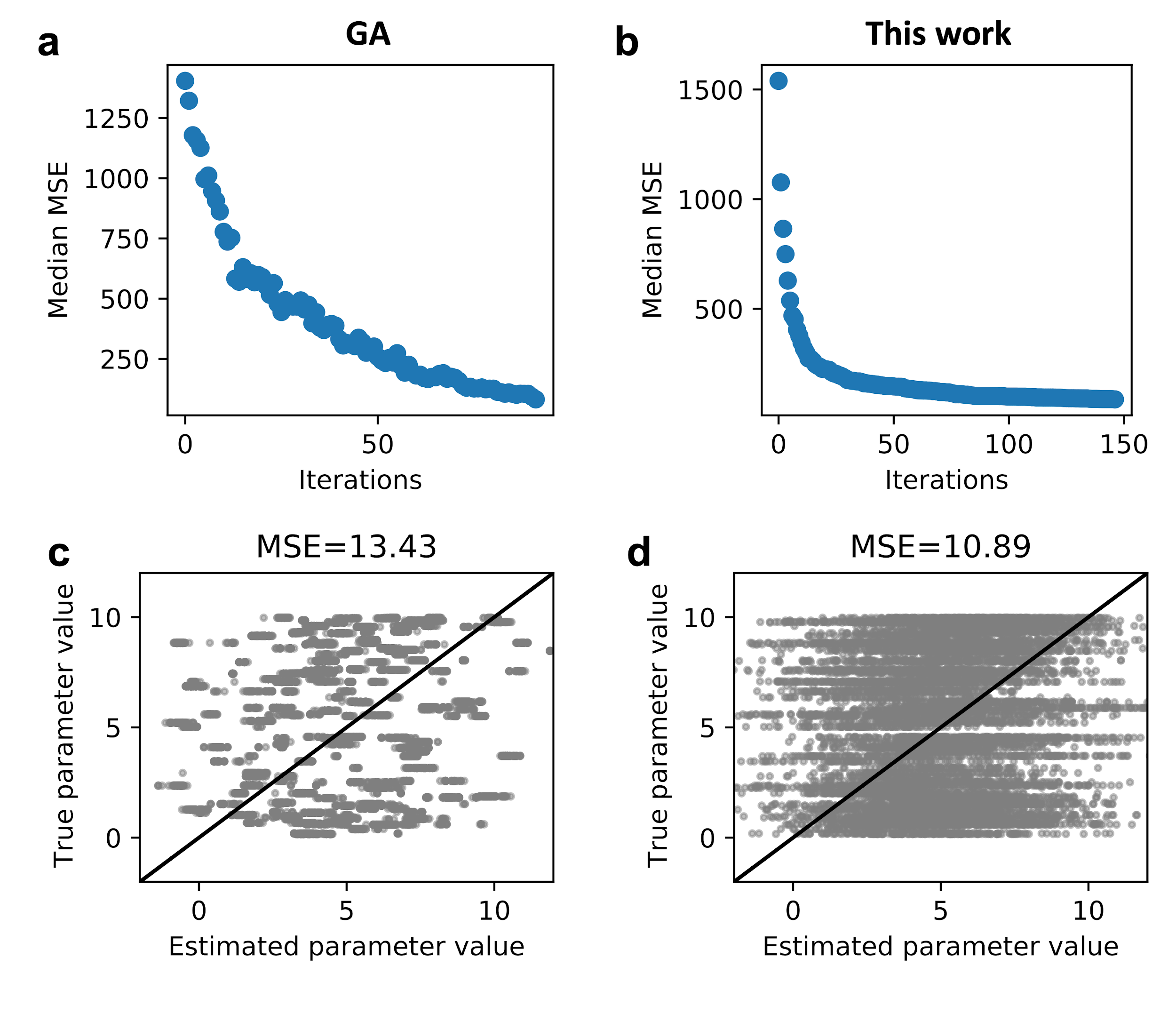


**Supplementary Figure** **19**. Validation results on the linear model $y=\sum_{i=1}^{100} a_{i}x_{i}$. (a-b) Median MSE values during iterations when using (a) genetic algorithm and (b) SMC-ABC approach proposed in this work. (c-d) Parity plot for comparison between true parameter values and 100 sets of estimated parameters when using (c) GA and (d) SMC-ABC approach proposed in this work. Mean squared error between true parameter values and mean values of 100 parameter sets are shown.


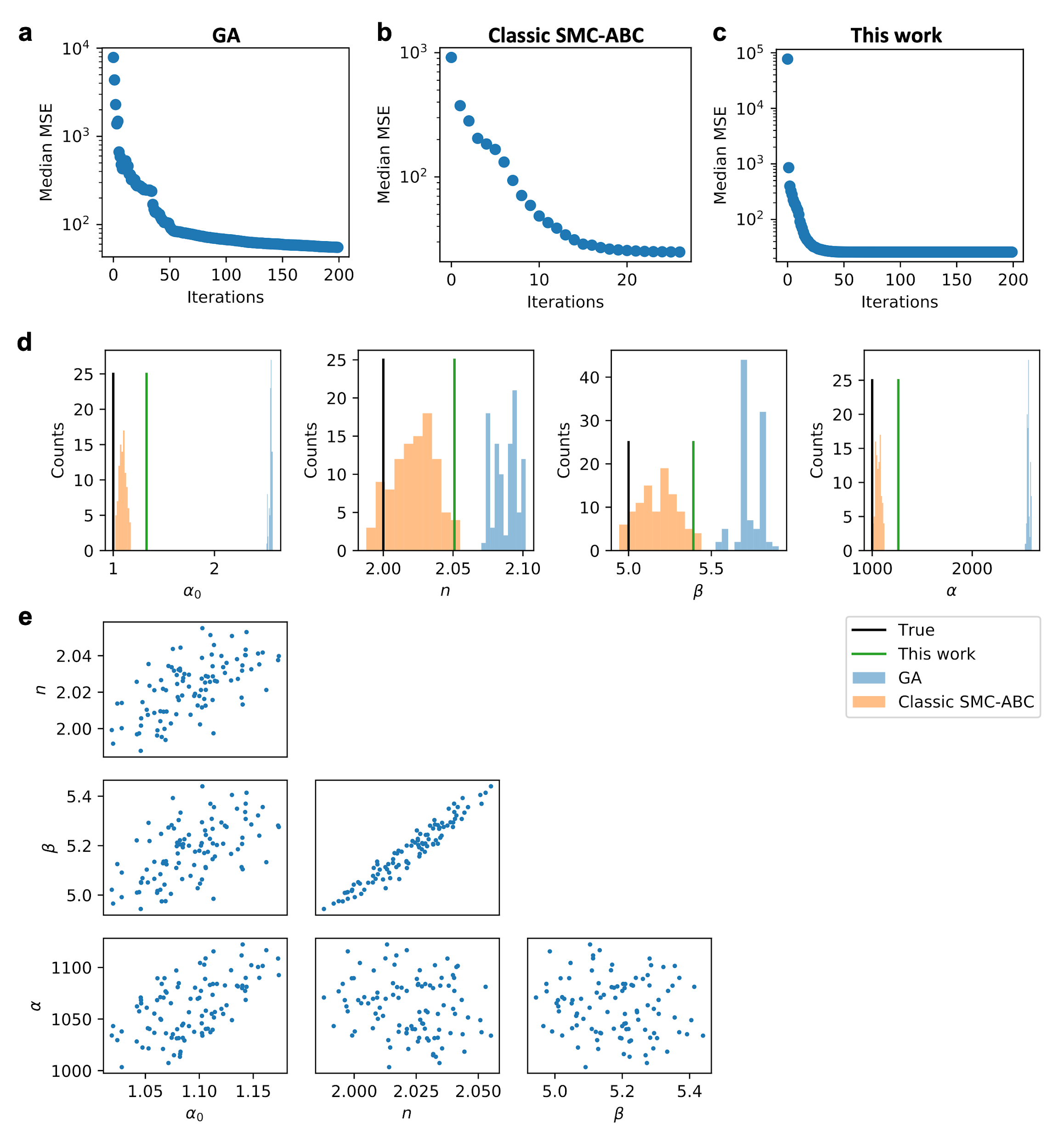


**Supplementary Figure** **20**. Validation results on the ODE model. (a-c) Median MSE values during iterations when using (a) genetic algorithm, (b) classical SMC-ABC and (c) SMC-ABC approach proposed in this work. (d) Distributions of inferred parameter values. (e) The scatter plots for parameters sampled by classic SMC-ABC approach.

## Supplementary Tables

**Supplementary Table 1.** Pseudo code for Sequential Monte Carlo based Approximate Bayesian Computation used in this work.

| Input: | Observed data $D$, distance function $\rho$ and the distance threshold $\epsilon$ |
| --- | --- |
| Output: | 100 samples from $P\left( \rho\left( D,\hat{D} \right)<\epsilon\right)$ |
| Initialize an empty set $S$ to store all $\hat{\theta}$ simulated | |
| Initialize an empty set $B$ to store the best 100 $\hat{\theta}$ after each step | |
| Repeat: |  |
|  | Sample 128 $\hat{\theta}$ from *Prior* distribution $P(\theta)$ |
|  | Simulate observed data $D$ to get $\hat{D}$ for all $\hat{\theta}$ |
|  | Calculate distance $\rho\left( D,\hat{D} \right)$ for all $\hat{\theta}$ |
|  | Add those 128 $\hat{\theta}$ into population set $S$ |
|  | Select the best 100 $\hat{\theta}$ from $S$ with smallest $\rho\left( D,\hat{D} \right)$ and replace old $\hat{\theta}$ in $B$ |
|  | Update $\epsilon_{t}$ with the minimal $\rho\left( D,\hat{D} \right)$ of $\hat{\theta}$ in $B$ |
|  | If $\epsilon_{t}\leq\epsilon$, break |
|  | Else: update *Prior* distribution $P(\theta)$ with $\hat{\theta}$ in $B$. Assume a normal distribution for each parameter $\theta_{i}$ in $\theta$ and use the mean and variance of $\theta_{i}$ in all 100 $\hat{\theta}$ in $B$ as the new mean and variance. |
| End |  |

**Supplementary Table 2**. List of strains.

| **Strain name** | **Genotype** | **Reference** |
| --- | --- | --- |
| IMX581 | *MATa ura3-52 can1∆::cas9-natNT2 TRP1 LEU2 HIS3* | [^13^](https://paperpile.com/c/9r1FLj/6t1T) |
| HL01 | *MATa ura3-52 can1∆::cas9-natNT2 TRP1 LEU2 HIS3 ERG1∆::kmERG1* | This study |

**Supplementary Table 3**. List of primers

| **Primer name** | **Sequences (5´-3´)** |
| --- | --- |
| tCYC1-X-2dn-R | GATAAATCTTCAGCATAGATGGGTAACGGGATCCCTCTGTGAGGGCCGATTATGCAGGCCTAGACCCGGCCGCAAATTAAAGCCTTCGAG |
| gRNA-ERG1-F | GTTGATAACGGACTAGCCTTATTTTAACTTGCTATTTCTAGCTCTAAAACAATGTTACTAGAGTGCAAGGGATCATTTATCTTTCACTGCGGAGAAGTTTCGAACGCCGAAACATGCGCA |
| gRNA-ERG1-R | TGCGCATGTTTCGGCGTTCGAAACTTCTCCGCAGTGAAAGATAAATGATCCCTTGCACTCTAGTAACATTGTTTTAGAGCTAGAAATAGCAAGTTAAAATAAGGCTAGTCCGTTATCAAC |
| kmEGR1-scERG1up-F | CAATTGTCCAGTATTGAACAATACAGGTTATTTCGAACAATTGAAAAAAAAAAATCACAGAAAAACATATCGAGAAAAGGGTCATGTCTTCAGCTACTGATAAGAAAG |
| kmEGR1-scERG1dn-R | GCCTTCCAAGCCGACTTCTGTAATAAAAAAAAAAGGTGCAGCTTAATGTTTGACGGTTCCTATCCTCTCTCCCTTATAAGCTGTAGTTAACCTGTCAATTCTCTAAAC |

###

## Supplementary references

1. [Toni, T., Welch, D., Strelkowa, N., Ipsen, A. & Stumpf, M. P. H. Approximate Bayesian computation scheme for parameter inference and model selection in dynamical systems. *J. R. Soc. Interface* **6**, 187–202 (2009).](http://paperpile.com/b/9r1FLj/ttp7D)

2. [Toni, T. & Stumpf, M. P. H. Simulation-based model selection for dynamical systems in systems and population biology. *Bioinformatics* **26**, 104–110 (2010).](http://paperpile.com/b/9r1FLj/r6TXp)

3. [Virtanen, P. *et al.* SciPy 1.0: fundamental algorithms for scientific computing in Python. *Nat. Methods* **17**, 261–272 (2020).](http://paperpile.com/b/9r1FLj/3Vqn2)

4. [Klinger, E., Rickert, D. & Hasenauer, J. pyABC: distributed, likelihood-free inference. *Bioinformatics* **34**, 3591–3593 (2018).](http://paperpile.com/b/9r1FLj/NoBCh)

5. [Prangle, D., Everitt, R. G. & Kypraios, T. A rare event approach to high-dimensional approximate Bayesian computation. *Stat. Comput.* **28**, 819–834 (2018).](http://paperpile.com/b/9r1FLj/iXIWM)

6. [Elowitz, M. B. & Leibler, S. A synthetic oscillatory network of transcriptional regulators. *Nature* **403**, 335–338 (2000).](http://paperpile.com/b/9r1FLj/2npsh)

7. [Zakhartsev, M., Yang, X., Reuss, M. & Pörtner, H. O. Metabolic efficiency in yeast Saccharomyces cerevisiae in relation to temperature dependent growth and biomass yield. *J. Therm. Biol.* **52**, 117–129 (2015).](http://paperpile.com/b/9r1FLj/WEnj)

8. [Caspeta, L. & Nielsen, J. Thermotolerant Yeast Strains Adapted by Laboratory Evolution Show Trade-Off at Ancestral Temperatures and Preadaptation to Other Stresses. *MBio* **6**, e00431 (2015).](http://paperpile.com/b/9r1FLj/oB6s)

9. [Postmus, J. *et al.* Quantitative analysis of the high temperature-induced glycolytic flux increase in Saccharomyces cerevisiae reveals dominant metabolic regulation. *J. Biol. Chem.* **283**, 23524–23532 (2008).](http://paperpile.com/b/9r1FLj/0T9i)

10. [Caspeta, L. & Nielsen, J. Thermotolerant Yeast Strains Adapted by Laboratory Evolution Show Trade-Off at Ancestral Temperatures and Preadaptation to Other Stresses. *MBio* **6**, e00431 (2015).](http://paperpile.com/b/9r1FLj/dBST)

11. [Doughty, T. W. *et al.* Stress-Induced Expression is Enriched for Evolutionarily Young Genes in Diverse Budding Yeasts. *bioRxiv* doi:](http://paperpile.com/b/9r1FLj/A49d)[10.1101/660274](http://dx.doi.org/10.1101/660274)[.](http://paperpile.com/b/9r1FLj/A49d)

12. [Sawle, L. & Ghosh, K. How do thermophilic proteins and proteomes withstand high temperature? *Biophys. J.* **101**, 217–227 (2011).](http://paperpile.com/b/9r1FLj/6bBf)

13. [Mans, R. *et al.* CRISPR/Cas9: a molecular Swiss army knife for simultaneous introduction of multiple genetic modifications in Saccharomyces cerevisiae. *FEMS Yeast Research* vol. 15 (2015).](http://paperpile.com/b/9r1FLj/6t1T)
