# Supplementary material for: Bayesian genome scale modelling identifies thermal determinants of yeast metabolism
Source: Nat Commun. 2021 Jan 8;12:190. doi: 10.1038/s41467-020-20338-2 (PMC7794507; doi:10.1038/s41467-020-20338-2)
Supplement: Supplementary file 3 — Reporting Summary [file 41467_2020_20338_MOESM3_ESM.pdf]

nature research

Corresponding author(s): Jens Nielsen

Last updated by author(s): Nov 12, 2020

Reporting Summary

Nature Research wishes to improve the reproducibility of the work that we publish. This form provides structure for consistency and transparency in reporting. For further information on Nature Research policies, see Authors & Referees and the Editorial Policy Checklist .

Statistics

For all statistical analyses, confirm that the following items are present in the figure legend, table legend, main text, or Methods section.

n/a	Confirmed	
☐	☒	The exact sample size (*n*) for each experimental group/condition, given as a discrete number and unit of measurement
☒	☐	A statement on whether measurements were taken from distinct samples or whether the same sample was measured repeatedly
☐	☒	The statistical test(s) used AND whether they are one- or two-sided<br>*Only common tests should be described solely by name; describe more complex techniques in the Methods section.*
☒	☐	A description of all covariates tested
☐	☒	A description of any assumptions or corrections, such as tests of normality and adjustment for multiple comparisons
☐	☒	A full description of the statistical parameters including central tendency (e.g. means) or other basic estimates (e.g. regression coefficient) AND variation (e.g. standard deviation) or associated estimates of uncertainty (e.g. confidence intervals)
☐	☒	For null hypothesis testing, the test statistic (e.g. *F*, *t*, *r*) with confidence intervals, effect sizes, degrees of freedom and *P* value noted<br>*Give P values as exact values whenever suitable.*
☐	☒	For Bayesian analysis, information on the choice of priors and Markov chain Monte Carlo settings
☐	☒	For hierarchical and complex designs, identification of the appropriate level for tests and full reporting of outcomes
☐	☒	Estimates of effect sizes (e.g. Cohen's *d*, Pearson's *r*), indicating how they were calculated

Our web collection on statistics for biologists contains articles on many of the points above.

Software and code

Policy information about availability of computer code

Data collection

For the prediction of enzyme Topt values, Tome v1.0 (Li et al., ACS Synth Biol.,2019) was used. This information is already in the manuscript.

Data analysis

All data analysis were carried out by Python scripts (mainly Jupyter notebooks) which are provided in a Github repository (https://github.com/SysBioChalmers/BayesianGEM). The link to the repository is included in the manuscript. The version of python packages:

numpy	1.15.0
pandas	0.23.4
scikit-learn	0.20.3
seaborn	0.9.0
jupyter	1.0.0
cobra	0.15.3
Gurobi	8.0.0

For manuscripts utilizing custom algorithms or software that are central to the research but not yet described in published literature, software must be made available to editors/reviewers. We strongly encourage code deposition in a community repository (e.g. GitHub). See the Nature Research guidelines for submitting code & software for further information.

Data

Policy information about availability of data

All manuscripts must include a data availability statement. This statement should provide the following information, where applicable:

- Accession codes, unique identifiers, or web links for publicly available datasets
- A list of figures that have associated raw data
- A description of any restrictions on data availability

The data for reproducing the figures in both main and supplementary file are provided as a Zenodo repository (https://zenodo.org/record/3996543#.X0J1BNP7S3I)

that is linked to scripts in the Github Repository (https://github.com/SysBioChalmers/BayesianGEM). The experimental datasets used for the development and validation of etcYeast model were manually collected from the various publications as referenced in the manuscript where they were mentioned.

Field-specific reporting

Please select the one below that is the best fit for your research. If you are not sure, read the appropriate sections before making your selection.

- [x] Life sciences
- [ ] Behavioural & social sciences
- [ ] Ecological, evolutionary & environmental sciences

For a reference copy of the document with all sections, see nature.com/documents/nr-reporting-summary-flat.pdf

Life sciences study design

All studies must disclose on these points even when the disclosure is negative.

Sample size	The sample size used in SMC-ABC part was chosen based on the computational power we have. We choose 128 (parallel purpose) for each iteration. With this sample size, the whole approach takes 3-5 days on a compute node with 32 cores (Intel Xeon Gold 6130 CPU). Five biological replicates were used for validation of ERG1. This number was chosen by considering the labour expense. It is also a common number of replicates used in testing the phenotype of genetically modified yeast strains.
Data exclusions	We didn't exclude any data.
Replication	The SMC-ABC approach was repeated once without specifying the random seed, by re-running the script performing SMC-ABC approach. The conclusions made in this manuscript are successfully reproduced, even though the exact values in the final results are slightly different. Future reproducibility is supported by making all scripts publicly available. The experimental validation results of ERG1 was also successfully reproduced once by performing another set of the identical experiments.
Randomization	Randomization is only relevant in SMC-ABC approach, where all values are randomly sampled from prior/posterior distributions without any seeds. In other section, the results are just the downstream analysis of the results from SMC-ABC approach without any randomization used.
Blinding	Not relevant to the study, as there was no group allocation involved.

Reporting for specific materials, systems and methods

We require information from authors about some types of materials, experimental systems and methods used in many studies. Here, indicate whether each material, system or method listed is relevant to your study. If you are not sure if a list item applies to your research, read the appropriate section before selecting a response.

Materials & experimental systems

n/a	Involved in the study	
x		Antibodies
x		Eukaryotic cell lines
x		Palaeontology
x		Animals and other organisms
x		Human research participants
x		Clinical data

Methods

n/a	Involved in the study	
x		ChIP-seq
x		Flow cytometry
x		MRI-based neuroimaging
