# Supplementary material for: Bayesian genome scale modelling identifies thermal determinants of yeast metabolism
Source: Nat Commun. 2021 Jan 8;12:190. doi: 10.1038/s41467-020-20338-2 (PMC7794507; doi:10.1038/s41467-020-20338-2)
Supplement: Supplementary file 4 — Description of Additional Supplementary Files [file 41467_2020_20338_MOESM4_ESM.pdf]

Description of Additional Supplementary Files

Supplementary Data 1
Codon-optimized kmERG1
