# Supplementary material for: Bayesian genome scale modelling identifies thermal determinants of yeast metabolism
Source: Nat Commun. 2021 Jan 8;12:190. doi: 10.1038/s41467-020-20338-2 (PMC7794507; doi:10.1038/s41467-020-20338-2)
Supplement: Supplementary file 2 — Peer Review File [file 41467_2020_20338_MOESM2_ESM.pdf]

Reviewer #1 (Remarks to the Author):

The article “Bayesian genome scale modelling identifies thermal determinants of yeast metabolism” by Li et al. combines constraint-based metabolic network flux simulation with Bayesian modeling of thermal parameters for enzyme activity and denaturation to predict metabolic shifts and temperature-sensitive metabolic bottlenecks in S. cerevisiae under a range of mostly superoptimal growth temperatures.
The authors include separate equations to distinguish the contributions of protein denaturation and macromolecular rate to enzyme activity as a function of temperature, which is more completely descriptive than prior efforts in the field to incorporate temperature constraints into genome-scale metabolic models and permits the authors to distinguish overall contribution of protein denaturation versus catalytic rates to temperature-dependent limitations of growth. In addition, the Bayesian approach to mitigate the uncertainty associated with a high number of parameters – many not yet experimentally measured – required to account for these terms describing the effect of temperature on enzyme activity network-wide shows impressive performance and is a clever use of machine learning in the context of genome-scale metabolic models. Both the more descriptive thermal terms and approach to deal with parameter uncertainty are important advances in this field.
Furthermore, the authors well-validate their modeling framework comparing to growth rates and specific flux data from wet lab experiments and recapitulating the metabolic shift associated with ATP maintenance at different temperature ranges. They then used their model framework to predict growth-limiting enzymes at superoptimal temperatures, most notably that ERG1 is the single most growth-limiting enzyme at 40°C, which they then validated by engineering a transgenic yeast strain with an ERG1 ortholog from a thermotolerant yeast.
Overall, this is a very high quality, well-executed study representing an important advance in the field. However, I find that there are some coarse assumptions and unaddressed points that should be analyzed more fully and with caveats discussed so as not to over-conclude from the results. On a more minor note, I also find that there are a number of typographical and figure numbering errors in the manuscript and recommend a thorough proofreading. Major and minor points follow.

Major points:
1. Given that they are using a machine learning approach to fill in the missing and uncertain thermal parameters required for their model while simultaneously predicting metabolic fluxes, the authors should evaluate how well their method has recapitulated experimentally measured values for these parameters. They describe the relationship in parity plots of Fig S6, but all this tells me is that their approach led to often rather different posteriors from priors. Just for the set of directly measured parameters (i.e. not those derived through assumptions and modeling), how closely do their posteriors match? How sensitive is the approach to missing thermal parameter data? It may not be necessary to perform traditional cross-validation here, but at least some assessment of sensitivity to these missing data types should be performed. The authors perform a limited cross-validation with respect to growth rate data (Fig S3) but not the thermal parameters.
2. The authors use a two-state model for protein denaturation (e.g. Fig 1b) that they and others previously have found works well for modeling at superoptimal growth temperatures; however, this model is almost certainly oversimplified at suboptimal temperatures and perhaps even more broadly below 40°C for some proteins. In the present study, below 40°C it is assumed that all proteins are in their native state. Denaturation and aggregation are also induced by sufficiently low temperatures [Lopez et al. J Phys Chem B. 2008. PMID:18181599; Stenzoski et al. Biophys J. 2018. PMID:30098729]. This is especially true for weakly stable proteins [Yan et al. Commun. Chem. 2018], which can have Tm and Tc that are separated by a narrow temperature range and can have Tc >> 0°C and in the physiological range of growth temperatures. Studying cold denaturation of the yeast protein frataxin [Pastore et al. J Am Chem Soc. 2007. PMID:17411056] is an important example in understanding this phenomenon. Effect of cold shock on proteomes has not been given nearly as much attention as heat shock, and thermal parameters necessary to model it are even sparser than for heat. Indeed it is unknown how many proteins exhibit the kind of weak stability exhibited by proteins with Tc in the physiological growth range, but given the genome-scale of this study, it could

very well be that some enzymes fall into this category. As such, it is perhaps misleading to conclude based on a simplified model of protein stability that loss of native state plays no role in metabolism below 40°C. I do not suggest that the authors need account for effects of these additional thermal parameters for the main purpose of the present study. Nevertheless, the authors should be very clear about the limitations of their predictions with respect to protein stability below 40°C and even moreso below OGT.

3. The authors highlight the average difference between experimentally-determined Topts and Tms in support of the claim that protein stability largely does not explain Topt but do not mention the analogous difference between Topts and the T at which kcat is 1/2 maximum. Their conclusion that kcat degeneration may largely explain Topt on its own requires this comparison.

4. Experimentally-measured Topts (Fig 3c) appear bimodally distributed, unlike the model prior and posterior values. Could the authors comment on this observation? Is there any relationship between values of Topt and up/downregulation of genes in response to superoptimal temperature, ergosterol pathway enzymes for instance? Does their model perhaps predict significant flux changes that distinguish enzymes with high experimental Topt from those with lower experimental Topt to satisfy flux balance constraints between 32°C and 40°C?

5. The authors observe by way of validation the recapitulation of ATP production shift from the respiratory pathway to glycolysis and claim that it is due both to the ATP-per-protein-mass efficiency combining with their total protein constraint and also that ATP1, HEM1, and PDB1 have relatively lower Tms. If the authors raise the Tms of these proteins or raise the total protein constraint, do they still observe the same shift? These tests would help to distinguish the contribution of each factor to this observed shift.

6. In their validation of ERG1 replacement with KmERG1, the authors passaged 6 times and compared their background strain to the engineered strain. In G1 there was no significant difference in growth, and in G2, it appeared that the background strain outgrew the KmERG1 strain with weak statistical significance. In successive passages up to G6, the KmERG1 strain significantly outgrew the background strain, but this effect waned with each passage. Given the observed diminishing effect, I would have expected the authors to continue with additional passages to see if the growth patterns of the strains converge after >6 days. Did the authors continue the experiment beyond G6, and if so, what was the result? Yeast can adapt to some extent to non-lethal stress over multiple generations simply by induced gene regulation, which seems to be exhibited by both the background and KmERG1 strains in their data after G2. Therefore, I would have expected the beneficial effect of KmERG1 to be more likely to show at an earlier generation after the shift from 30°C to 40°C before the difference in ERG1 protein stability and activity could be diluted-out by adaptation. It seems those early generations had the opposite effect to that intuition if any. Further complicating the issue, as the authors note, ERG1 and other ergosterol pathway enzymes are downregulated transcriptionally and translationally at superoptimal temperatures. They posit that this could have to do with resource conservation to increase fitness, but if that is the case, how would replacement of just one enzyme far upstream in the pathway be expected to compensate for the likelihood of a new pathway bottleneck due to downregulation of 20 enzymes in the pathway? Do they suspect that squalene or squalene-2,3-epoxide could act as a regulator of these downstream genes to rescue their expression in the KmERG1 strain? Such a claim could be tested by transcriptome profiling, but that seems beyond the scope this study. Could the authors at least layer additional constraints on the ERG genes in their model to represent their downregulation both with and without the thermal constraint of ERG1 to see if their model still predicts the ERG1 rescue? More generally, the authors should discuss the noted experimental observations and offer more comprehensive analysis in the manuscript.

7. Model validations for specific growth rate and specific fluxes (Fig S2) appear quite accurate for T<36°C but noticeably less accurate thereafter. The authors should comment on this observation in the text.

Minor points:

1. The authors introduce some new acronyms (etcGEM, GETCool) in a field that already has a high volume of acronyms and jargon. It is my opinion that such practice should be kept to a minimum to avoid alienating non-experts. GETCool is especially egregious given that the authors never define this

as an acronym in the manuscript. It can be useful to abbreviate complex concepts like their method, but it isn't very helpful if there is no mnemonic relationship defined between the abbreviation and the concept itself. The nested layers of jargon represented in a term like Posterior etcGEM (etymology: GEM --> ecGEM --> etcGEM --> Posterior etcGEM) may also be confusing to non-experts, although the authors do define each of these reasonably well.
2. Typgraphical errors:
a. "Principal component analysis of all 21,504 parameter sets generated in the approach showed how the Priori distributions were gradually updated to distinct Posterior distributions (Fig 2d)." should read "Principal component analysis of all 21,504 parameter sets generated in the approach showed how the Prior distributions were gradually updated to distinct Posterior distributions (Fig 2d)."
b. "Predicted maximal specific growth rate of wide-type yeast and the one without any temperature constraints (fully functional) on ERG1 enzyme at 40 °C." should read "Predicted maximal specific growth rate of wild-type yeast and the one without any temperature constraints (fully functional) on ERG1 enzyme at 40 °C."
c. "Furthermore, mitochondria only exists in eukaryotes and almost all of them have evolved to have an optimal growth temperature below 40 °C 3." should read "Furthermore, mitochondria only exist in eukaryotes and almost all of them have evolved to have an optimal growth temperature below 40 °C 3."
3. The legend for Fig S3 should include more explicit definitions of panels a, b, and c. I can see that they represent each fold of the validation, but that may not be immediately obvious to all readers.
4. The legend for Fig S5 does not explain the blue areas in the plots.
5. In the supplement there are two Fig S9s and two Fig S10s. The authors need to fix the figure numbering and also double-check the references to these figure numbers in the text throughout. Also, the description "The strains were cultivated at 40°C for six generations to reach the steady state of growth" corresponds to Fig 5; the first Fig S9 should say "cultivated at 42°C for two generations."

Roger Larken Chang, Ph.D.
Associate Lab Director
Department of Systems Biology
Harvard Medical School

Reviewer #2 (Remarks to the Author):

In this manuscript, the authors aim to incorporate enzyme temperature dependences into genome scale matabolic models, using a machine learning approach to determine and optimize the temperature dependent properties of all enzymes contributing to the fluxes in the model. They start with a well-established GEM of the yeast Saccharomyces cerevisiae, and describe the temperature dependent properties of enzymes as the T dependent folded/functional fraction and an Arrhenius function for kcat, to generate T dependent specific activities based on known, set temperature kinetics, on primary sequences. The model they generate in this way with known data does not reflect available data describing temperature dependent growth and central carbon metabolic fluxes. From this prior model, they optimize the Tdependent parameters using statistical methods. After optimization, they can predict an important contribution of several mitochondrial proteins and (reduction of) mitochondrial ATP flux to the reduced respiratory rate and reduced growth at high temperatures, as well as a strong temprature sensitivity of ergosterol synthesis. The latter is confirmed when the authors supplement cerevisiae ERG1 with the ERG1 from the temperature tolerance Kluyveromyces marxianus, which indeed imprves growth at high temperatures.
My main comment stems possibly from lack of precise understanding of the consequences of the fitting approach taken, but I could also not clearly find information on this topic in the manuscript or its supplements: I presume all datasets on temperature dependent bahavior were used in the optimization? I cannot find if some of the data were used as a trainingset, to show that other data were now more closely reproduced? Should such an approach not be used in these kinds of simulations? Because several properties of all enzymes could be optimized, this is essentially a fit with

many variables, so what is the independent validation of he fit if there is no separation between training and testing datasets? Related to this, after the fitting the model fits better, which is logical. One of the major disadvantages of machine learning approaches is that we do not know whether the optimization performed to make the model fit the data, are actually reflecting the optimization that has taken place in nature during evolution, to adjust cellular functioning to temperature, in this case. The authors do carefully analyze the changes that have been introduced during the machine learning optimization, and find that particularly the changes in Topt contribute to the better fit, and that the major change there is the statistical uncertainty, or the variance. I cannot see from the data presented how much these have changed, only the number of proteins for whcih they have changed (Figure 2e). Also, the PCA analysis that appears to describe the fitting trajectory, and the separation of the prior and posterior model fits, has a PC1 whcih only explains 1.15% of the model adjustment, whcih basically implies that there is no way you can truly analyze what has brought about the better fit. I am of the opinion that there should be a validation of the biological relevance of the changes in model parameters, probably tested in vitro. Do the fitted parameters indeed better describe enzyme temperature dependence?

The second major comment concerns the following: Each enzyme specific rate was described as a combination of "active fraction" and T dependent kcat. In figure 1a, the term [E]N(T)is also affected by expression levels, and the term kB also depends on the specific isoenzyme performing the reaction. How does the model account for changes in expression levels, affecting reaction capacity without affecting T dependent active fraction, and changes in isoenzymes expressed, which affect both T dependence of kcat (as it is a different enzyme performing the same reaction) and T dependence of the active fraction (as it has a different primary sequence/ stability, etc).
A last major remark concerns the conclusions about the mechanism for the strong reduction of mitochondrial flux at high temperatures, which the authors attribute to a constraint in total protein synthetic capacity. They themselves already observe a strong temperature dependence of several mitochondrial proteins, how are the two quantitatively contributing? Besides, other authors have suggested a strong temperature dependence of the chaperone machinery for protein import into the mitochondria (Moro and Muga, 2006, https://doi.org/10.1016/j.jmb.2006.03.027) and a defect in mitochondrial biosynthesis/repair (Postmus et al., 2011, https://doi.org/10.1099/mic.0.050039-0). How are these observation related/relatable to the mechnisms inferred form the model?

Minor remarks
page numbers and line numbers really facilitate the reviewing process
In the first paragraph of the introduction the authors generously distribute term like the most common (environmental parameter), the most abundant, the most sensitive and the largest (effect), where the objectivity can be disputed. I particularly struggled with temperature being the most common parameter affecting cellular (cellular?) evolution. I would assume competition to be the most common, but this is an argument that would take forever, and cannot be objectively substantiated. The same hold for these other "quantitative" statements. Please adjust.
2nd paragraph of intro, 2nd sentence is incomplete
4th paragraph, Much data, not multiple data
page 7, paragraph 3: "Here, we first used....approach" is a sentence that is grammatically incorrect and therefore incomprehensible
Figure 2: explain colors in legend, and in panel a rather than d
Figure title: statistical uncertainties, because true uncertainties haven't been changed (yet)
Figure 3 d-f, use color-blind friendly colors.
Figure 3 caption, last word, the same instead of equal?
The erg1 mutant was transferred/passaged multiple times, but this is not the same as generations (=cell number duplications), so growth from OD0.1 to OD1.8 is >4 generations within one transfer. Please correct in text and figure.
Figure 5c, why is growth reduced for both strains after the first passaging? Why does WT also recover after 6 transfers?
Figure 5, caption, I presume the cells were grown until glucose/carbon depletion/stationary phase (a

discussion by itself) rather than to steady state?
Gertien Smits

Reviewer #3 (Remarks to the Author):

In this article, the authors develop a new methodology to incorporate temperature-dependent enzyme activity into genome-scale metabolic models through a Bayesian estimation technique. In the technique, the authors develop a model of temperature-dependent enzyme activity that integrates with flux balance analysis modeling. This model requires parameters for each enzyme that determine how its activity changes with respect to temperature. The authors propose a technique that uses three experimental measurements for each enzyme: melting temperature, heat capacity change, and optimal temperature. These measurements are used as prior distributions for a Bayesian inference optimization. When these experimental measurements are missing, the authors use mean values. Using a new sequential monte-carlo, approximate Bayesian inference technique, the authors find distributions of temperature-dependent enzyme parameters that are consistent with a dataset of growth rates and ethanol fluxes for yeast. From these 'posterior' distributions, the authors identify ERG1 as a key bottleneck in the thermo-tolerant growth of the wild-type organism. Experimental integration of a thermo-tolerant ERG1 appears to improve the growth rate after several generations of adaptive laboratory evolution vs wild-type.

Incorporation of thermotolerance and temperature-dependent growth into predictive metabolic models is indeed an important research topic, and the use of Bayesian modeling to reconcile single-enzyme measurements with systems-level growth rates does indeed appear to be a good way of addressing this issue. However, I have some concerns with the way this strategy was implemented in this study. Most concerning is the sequential monte carlo approach employed by the authors to improve the convergence of their approximate bayesian computation (ABC) algorithm. A key modification they have made to the ABC approach is that the prior distributions are 'updated' during each iteration to be closer to the parameter sets that have been shown to match experimental data. While this would indeed result in more parameter sets that match experimental data, it likely reduces the procedure to a simple stochastic optimization approach (i.e., a genetic algorithm or particle swarm), rather than an estimation of a true posterior distribution. More simply, the prior distributions include information on experimental measurements of enzyme temperature dependence -- by abandoning these priors, the authors likely loose the influence of these measurements on their final parameter distributions. As a result, the authors may be overfitting the relatively few growth and ethanol flux measurements they use as evaluation criteria.

Additional comments:
- using experimental error for the standard deviation of priors with missing measurements seems incorrect, typically you use a wide prior to express uncertainty in those measurements.
- for the experimental confirmation, the wild-type doesn't appear to have reached steady-state growth; additional ALD might make the difference statistically insignificant. I'm also confused why the first generation has such high OD compared to the second generation.

Minor Comments:
- "Fig SX" in Figure 3 caption is unclear
- Fig 5 caption: wide-type instead of wild-type
- how many data points are used during the ABC calculation? This should be spelled out more clearly.
- what's the difference between fig 5C and fig S9?

Response to Reviewers (R: refers to our response):

We thank the reviewers for the constructive comments. Our point-by-point replies to each reviewer's comments are detailed below. All text changes were highlighted in red in the revised manuscript.

REVIEWER COMMENTS

Reviewer 1 comments:

The article "Bayesian genome scale modelling identifies thermal determinants of yeast metabolism" by Li et al. combines constraint-based metabolic network flux simulation with Bayesian modeling of thermal parameters for enzyme activity and denaturation to predict metabolic shifts and temperature-sensitive metabolic bottlenecks in S. cerevisiae under a range of mostly superoptimal growth temperatures.

The authors include separate equations to distinguish the contributions of protein denaturation and macromolecular rate to enzyme activity as a function of temperature, which is more completely descriptive than prior efforts in the field to incorporate temperature constraints into genome-scale metabolic models and permits the authors to distinguish overall contribution of protein denaturation versus catalytic rates to temperature-dependent limitations of growth. In addition, the Bayesian approach to mitigate the uncertainty associated with a high number of parameters – many not yet experimentally measured – required to account for these terms describing the effect of temperature on enzyme activity network-wide shows impressive performance and is a clever use of machine learning in the context of genome-scale metabolic models. Both the more descriptive thermal terms and approach to deal with parameter uncertainty are important advances in this field.

Furthermore, the authors well-validate their modeling framework comparing to growth rates and specific flux data from wet lab experiments and recapitulating the metabolic shift associated with ATP maintenance at different temperature ranges. They then used their model framework to predict growth-limiting enzymes at superoptimal temperatures, most notably that ERG1 is the single most growth-limiting enzyme at 40°C, which they then validated by engineering a transgenic yeast strain with an ERG1 ortholog from a thermotolerant yeast.

Overall, this is a very high quality, well-executed study representing an important advance in the field. However, I find that there are some coarse assumptions and unaddressed points that should be analyzed more fully and with caveats discussed so as not to over-conclude from the results. On a more minor note, I also find that there are a number of typographical and figure numbering errors in the manuscript and recommend a thorough proofreading. Major and minor points follow.

R: We sincerely thank reviewer 1 for the insightful evaluation and constructive comments.

Major points:

1. Given that they are using a machine learning approach to fill in the missing and uncertain thermal parameters required for their model while simultaneously predicting metabolic fluxes, the authors should evaluate how well their method has recapitulated experimentally measured values for these parameters. They describe the relationship

in parity plots of Fig S6, but all this tells me is that their approach led to often rather different posteriors from priors. Just for the set of directly measured parameters (i.e. not those derived through assumptions and modeling), how closely do their posteriors match? How sensitive is the approach to missing thermal parameter data? It may not be necessary to perform traditional cross-validation here, but at least some assessment of sensitivity to these missing data types should be performed. The authors perform a limited cross-validation with respect to growth rate data (Fig S3) but not the thermal parameters.

R: Thanks for this comment. As suggested, we performed comparison between the T_m with experimental measurement (used for *Prior*) and corresponding values from 100 *Posterior* models. The results showed that the mean values from *Posterior* models are strongly correlated with experimental measurements (Fig 2g).

For T_{opt}, the *Prior* estimations were predicted by a machine learning approach since only 14 enzymes with known T_{opt} can be mapped to the etcYeast model using data from the BRENDA database. The T_{opt} of those 14 enzymes in the *Posterior* showed weak correlation with experimental values (Fig 2h). However, there are 662 T_{opt} records in BRENDA that can be mapped to *S. cerevisiae* although they cannot be mapped to specific enzymes. The comparison between the distributions of the mean values in *Posterior* models and those values from BRENDA showed a good agreement with each other (Fig 2i).

All the parameters in the model can be considered as missing values. Experimental measurements and other data- or assumption-based estimations only differ in their accuracies, which are reflected as the uncertainties in the resulting values. As shown in Fig S6, although the uncertainties (variances) in *Posterior* are reduced for 204 T_m, 449 Topt and 128 $\Delta C_p^{\ddagger}$, there were still big uncertainties in the *Posterior* models (e.g. T_{opt}, 10.9 vs 7.1 °C; T_m, 4.9 vs 4.0 °C; $\Delta C_p^{\ddagger}$, 2.0 vs 1.8 kJ/mol/K, average standard variance comparison between *Prior* and *Posterior*, Fig S7). With such big uncertainties, the *Posterior* models can still reproduce the experimental data in Figs 2a-c and the uncertainties in predictions in Fig 4 is small, indicating the robustness of the approach to the uncertainties in parameters. In principle, a good (close to *Posterior*) or bad (far from *Posterior*) *Prior* estimation impacts the efficiency of ABC approach the most, while less on the performance of the resulting *Posterior* models, since all the *Posterior* models are forced to accurately reproduce the experimental data.

In addition, we also tried to use 50% of data (training dataset) to generate *Posterior* models and then test their performance on the rest 50% data (test dataset) (Fig S4). The results showed that the *Posterior* models performed similarly on both training and test dataset, indicating the high generalizability of *Posterior* models obtained from SMC-ABC approach.
New figures Fig S4, Fig 2g and 2h were added in the revised MS. Fig 3c was re-indexed as Fig 2i. Fig S7 was also updated by including the distribution comparisons. Associated results and discussions were added in the revised MS (lines 159-168, 185-200).

2. The authors use a two-state model for protein denaturation (e.g. Fig 1b) that they and others previously have found works well for modeling at superoptimal growth temperatures; however, this model is almost certainly oversimplified at suboptimal temperatures and perhaps even more broadly below 40°C for some proteins. In the present study, below 40°C it is assumed that all proteins are in their native state. Denaturation and aggregation are also induced by sufficiently low temperatures [Lopez et al. J Phys Chem B. 2008. PMID:18181599; Stenzoski et al. Biophys J. 2018. PMID:30098729]. This is especially true for weakly stable proteins [Yan et al. Commun. Chem. 2018], which can have Tm and Tc that are separated by a narrow temperature range and can have Tc >> 0°C and in the physiological range of growth temperatures. Studying cold denaturation of the yeast protein frataxin [Pastore et al. J Am Chem Soc. 2007. PMID:17411056] is an important example in understanding this phenomenon. Effect of cold shock on proteomes has not been given nearly as much attention as heat shock, and thermal parameters necessary to model it are even sparser than for heat. Indeed it is unknown how many proteins exhibit the kind of weak stability exhibited by proteins with Tc in the physiological growth range, but given the genome-scale of this study, it could very well be that some enzymes fall into this category. As such, it is perhaps misleading to conclude based on a simplified model of protein stability that loss of native state plays no role in metabolism below 40°C. I do not suggest that the authors need account for effects of these additional thermal parameters for the main purpose of the present study. Nevertheless, the authors should be very clear about the limitations of their predictions with respect to protein stability below 40°C and even more so below OGT.

R: We agree that the two-state protein denaturation is an oversimplified assumption, which may not be true for some yeast enzymes. We do not think the two-state model assumes the protein is in native state at suboptimal temperatures. It can describe the cold-induced denaturation as well as heat-induced denaturation. In the resulting *Posterior* model, most proteins were found to denature at temperatures either higher than about 40 °C (heat-induced) or lower than 0 °C (cold-induced) (Fig 3c).

We updated the Results and Discussion sections to describe this better (lines 253-258, 424-429).

3. The authors highlight the average difference between experimentally-determined Topts and Tms in support of the claim that protein stability largely does not explain Topt but do not mention the analogous difference between Topts and the T at which kcat is 1/2 maximum. Their conclusion that kcat degeneration may largely explain Topt on its own requires this comparison.

R: We added a comparison between $T_{1/2SA}$, the temperature at which the specific activity is 50% of the maximum, and $T_{1/2kcat}$, the temperature at which kcat is 50% of its maximum and T_m in Fig 3c, as well as associated comments in the revised MS (lines 242-243).

4. Experimentally-measured Topts (Fig 3c) appear bimodally distributed, unlike the model prior and posterior values. Could the authors comment on this observation?

R: The bimodal distribution of T_{opt} values is due to the common values in BRENDA, these T_{opt}s are room temperature 25 °C (111 times), optimal growth temperature of yeast 30 °C (244 times) and 37 °C (142 times) that were frequently measured in experiments. These common values make the T_{opt}s bimodally distributed with two peaks at 30 °C and 37 °C. In the revised MS, Fig 3c was reindexed as Fig 2i and these comments were added into the caption of Fig 2i. Comments were also added in the revised MS (lines 216-219).

5. Is there any relationship between values of Topt and up/downregulation of genes in response to superoptimal temperature, ergosterol pathway enzymes for instance?

R: To address this question, we plotted *Posterior* T_{opt} mean against log2-Fold change of genes in response to superoptimal temperature obtained from Doughty T et al (PMID: 32358542), and found that there is no strong correlation between them (*Pearson*'s r on all genes: r = -0.06, *p-value* = 0.10; on gens in ergosterol pathway: r = -0.31, *p-value* = 0.17). The uncertainties in the *Posterior* T_{opt} values (Fig S6) may prevent us from identifying any significant association.

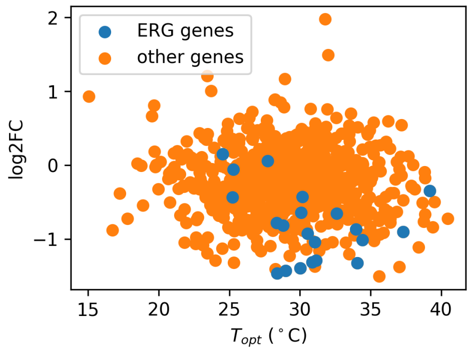

6. Does their model perhaps predict significant flux changes that distinguish enzymes with high experimental Topt from those with lower experimental Topt to satisfy flux balance constraints between 32°C and 40°C?

R: There are 14 enzymes with known experimental T_{opt} involved in 43 reactions in the model. The maximal fluxes through the reactions catalyzed by these enzymes were obtained by flux variation analysis at 32 and 40 °C. As shown in the below figure, there is no strong correlation between flux changes and enzyme T_{opt}s, thereby at least for those 14 enzymes, the flux changes that cannot be used to distinguish enzymes with different experimental T_{opt}.

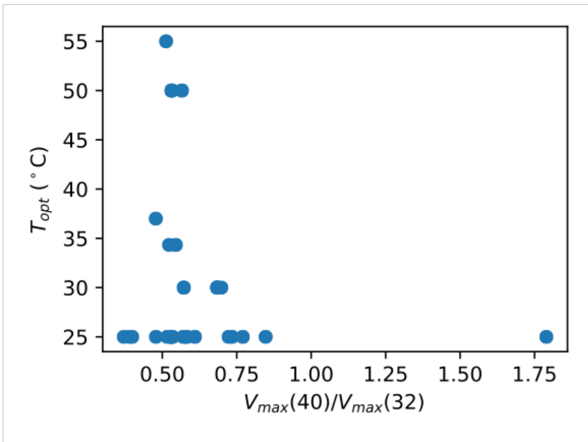

7. The authors observe by way of validation the recapitulation of ATP production shift from the respiratory pathway to glycolysis and claim that it is due both to the ATP-per-protein-mass efficiency combining with their total protein constraint and also that ATP1, HEM1, and PDB1 have relatively lower Tms. If the authors raise the Tms of these proteins or raise the total protein constraint, do they still observe the same shift? These tests would help to distinguish the contribution of each factor to this observed shift.

R: Yes, there is still a metabolic shift observed when doubling the total protein constraint (Fig 4b) or raising the T_m of three mitochondrial enzymes (ATP1, HEM1 and PDB1) to 50 °C (Fig 4c). By doubling the total protein constraint, the metabolic shift occurs at around 38 °C when the total protein usage hits the upper bound. With increased T_m of those three enzymes, the shift occurs at the same temperature point (~37 °C) as the original one, while the ATP production shift from mitochondria to glycolysis is reduced at temperatures higher than the shift temperature point. These simulations support our claims that this temperature induced metabolic shift can be explained by ATP-per-protein-mass efficiency combining with their total protein constraint.
To clarify this effect, we updated Fig 4 by adding two additional panels: one with increased T_m of ATP1, HEM1 and PDB1, and another with doubled total protein constraint. The corresponding Results section was also updated accordingly (lines 288-289, 296-298).

8. In their validation of ERG1 replacement with KmERG1, the authors passaged 6 times and compared their background strain to the engineered strain. In G1 there was no significant difference in growth, and in G2, it appeared that the background strain outgrew the KmERG1 strain with weak statistical significance. In successive passages up to G6, the KmERG1 strain significantly outgrew the background strain, but this effect waned with each passage. Given the observed diminishing effect, I would have expected the authors to continue with additional passages to see if the growth patterns of the strains converge after >6 days. Did the authors continue the experiment beyond G6, and if so, what was the result? Yeast can adapt to some extent to non-lethal stress over multiple generations simply by induced gene regulation, which seems to be exhibited by both the background and KmERG1 strains in their data after G2. Therefore, I would have expected the beneficial effect of KmERG1 to be more likely to show at an earlier generation after the shift from 30°C to 40°C before the difference in ERG1 protein

stability and activity could be diluted-out by adaptation. It seems those early generations had the opposite effect to that intuition if any. Further complicating the issue, as the authors note, ERG1 and other ergosterol pathway enzymes are downregulated transcriptionally and translationally at superoptimal temperatures. They posit that this could have to do with resource conservation to increase fitness, but if that is the case, how would replacement of just one enzyme far upstream in the pathway be expected to compensate for the likelihood of a new pathway bottleneck due to downregulation of 20 enzymes in the pathway? Do they suspect that squalene or squalene-2,3-epoxide could act as a regulator of these downstream genes to rescue their expression in the KmERG1 strain? Such a claim could be tested by transcriptome profiling, but that seems beyond the scope this study. Could the authors at least layer additional constraints on the ERG genes in their model to represent their downregulation both with and without the thermal constraint of ERG1 to see if their model still predicts the ERG1 rescue? More generally, the authors should discuss the noted experimental observations and offer more comprehensive analysis in the manuscript.

R: We thank the reviewer for this very good comment. Firstly, we repeated the experiments for 7 passages. It shows that after 3 passages of adaptation, both WT and KmERG1 strains reach the steady stage and the KmERG1 strain grows significantly better than WT strain (Fig 5c).

Secondly, to test the effect of down-regulation of genes in ergosterol pathway, we simulated the maximal specific growth rate of strains with a wild-type ERG1 or a temperature insensitive ERG1 by down-regulating other genes (ERG10, ERG13, HMG1, HMG2, ERG12, ERG8, MVD1, IDI1, ERG20, ERG9, ERG7, ERG11, ERG24, ERG25, ERG26, ERG27, ERG6, ERG2, ERG3, ERG5 and ERG4) in the ergosterol pathway. The down-regulation was done by firstly determining the maximal flux (V_{max}) through reactions catalyzed by those enzymes with flux variability analysis and then constraining the upper bound of those reactions by multiplying V_{max} with a percentage value. The simulation showed that when those enzymes are down-regulated to a percentage level higher than about 40%, a temperature-insensitive ERG1 can rescue the cell growth, while it fails when they are down-regulated to less than 40% (Fig S11). Thereby there should be a critical down-regulation level (40% in this case) that when the down-regulation is higher than this level, the ERG1 is the rate-limiting enzyme and over-expression of other enzymes in the ergosterol pathway is not necessary.

Based on these new simulations we updated the discussion section about ERG1 in the Discussion section (lines 400-403).

9. Model validations for specific growth rate and specific fluxes (Fig S2) appear quite accurate for T<36°C but noticeably less accurate thereafter. The authors should comment on this observation in the text.

R: As requested, we expanded the comments about validation accuracy at higher temperatures in the revised MS (lines 147-158).

Minor points:

1. The authors introduce some new acronyms (etcGEM, GETCool) in a field that already has a high volume of acronyms and jargon. It is my opinion that such practice should be kept to a minimum to avoid alienating non-experts. GETCool is especially egregious given that the authors never define this as an acronym in the manuscript. It can be useful to abbreviate complex concepts like their method, but it isn't very helpful if there is no mnemonic relationship defined between the abbreviation and the concept itself. The nested layers of jargon represented in a term like Posterior etcGEM (etymology: GEM --> ecGEM --> etcGEM --> Posterior etcGEM) may also be confusing to non-experts, although the authors do define each of these reasonably well.

R: As suggested, we removed the term GETCool.

2. Typographical errors:

a. "Principal component analysis of all 21,504 parameter sets generated in the approach showed how the Priori distributions were gradually updated to distinct Posterior distributions (Fig 2d)." should read "Principal component analysis of all 21,504 parameter sets generated in the approach showed how the Prior distributions were gradually updated to distinct Posterior distributions (Fig 2d)."

R: Corrected from "*Priori*" to "*Prior*" (line 175).

b. "Predicted maximal specific growth rate of wide-type yeast and the one without any temperature constraints (fully functional) on ERG1 enzyme at 40 °C." should read "Predicted maximal specific growth rate of wild-type yeast and the one without any temperature constraints (fully functional) on ERG1 enzyme at 40 °C."

R: Corrected from "wide-type" to "wild-type" (line 334).

c. "Furthermore, mitochondria only exists in eukaryotes and almost all of them have evolved to have an optimal growth temperature below 40 °C 3." should read "Furthermore, mitochondria only exist in eukaryotes and almost all of them have evolved to have an optimal growth temperature below 40 °C 3."

R: "exists" changed to "exist" (line 418).

3. The legend for Fig S3 should include more explicit definitions of panels a, b, and c. I can see that they represent each fold of the validation, but that may not be immediately obvious to all readers.

R: We have updated the legend in the revised MS.

4. The legend for Fig S5 does not explain the blue areas in the plots.

R: The descriptions for the blue areas were added to the legend of Fig S5 (Now Fig S6 in the revised MS).

5. In the supplement there are two Fig S9s and two Fig S10s. The authors need to fix the figure numbering and also double-check the references to these figure numbers in the text throughout. Also, the description "The strains were cultivated at 40°C for six generations to reach the steady state of growth" corresponds to Fig 5; the first Fig S9 should say "cultivated at 42°C for two generations."

R: Both the figures and their captions were revised as suggested by the reviewer.

Reviewer #2 (Remarks to the Author):

In this manuscript, the authors aim to incorporate enzyme temperature dependences into genome scale metabolic models, using a machine learning approach to determine and optimize the temperature dependent properties of all enzymes contributing to the fluxes in the model. They start with a well-established GEM of the yeast Saccharomyces cerevisiae, and describe the temperature dependent properties of enzymes as the T dependent folded/functional fraction and an Arrhenius function for kcat, to generate T dependent specific activities based on known, set temperature kinetics, on primary sequences. The model they generate in this way with known data does not reflect available data describing temperature dependent growth and central carbon metabolic fluxes. From this prior model, they optimize the T-dependent parameters using statistical methods. After optimization, they can predict an important contribution of several mitochondrial proteins and (reduction of) mitochondrial ATP flux to the reduced respiratory rate and reduced growth at high temperatures, as well as a strong temperature sensitivity of ergosterol synthesis. The latter is confirmed when the authors supplement cerevisiae ERG1 with the ERG1 from the temperature tolerance Kluyveromyces marxianus, which indeed improves growth at high temperatures.

R: We sincerely thank Reviewer 2 for detailed comments on our manuscript!

My main comment stems possibly from lack of precise understanding of the consequences of the fitting approach taken, but I could also not clearly find information on this topic in the manuscript or its supplements: I presume all datasets on temperature dependent bahavior were used in the optimization? I cannot find if some of the data were used as a trainingset, to show that other data were now more closely reproduced? Should such an approach not be used in these kinds of simulations? Because several properties of all enzymes could be optimized, this is essentially a fit with many variables, so what is the independent validation of he fit if there is no separation between training and testing datasets?

R: To assess the quality of the models, three datasets were used that included (i) the maximal specific growth rate in aerobic batch cultivations (number of temperature points *n* = 8), (ii) anaerobic batch cultivations (*n* = 8), and (iii) fluxes of carbon dioxide (CO_2), ethanol and glucose in chemostat cultivations (*n* = 6) (Fig S2, Methods M5).

Two different validations were performed. In the first three-fold cross-validation (Fig S3), two above datasets were used to fit the model with SMC-ABC approach and then validated on the third one. The results showed that there was both overlapping and orthogonal information among the datasets (Fig S3), suggesting that all three datasets should be used for updating the model parameters.

In the second validation, all three datasets were combined together and divided into training (50%) and test (50%) datasets based on the temperature points (Fig S4). The results showed that the obtained *Posterior* models performed similarly on the training and test datasets (Training median R^2 score: 0.90, 5-95% percentile range: [0.89, 0.93]; Testing median R^2 score: 0.84, 5-95% percentile range: [0.71,0.91]), indicating the high generalizability of the *Posterior* models (Fig S4).

Finally, all three datasets were combined and used to fit the final *Posterior* models, which were used for interpretation and prediction in the whole paper.

We have updated the results section to make it clearer in the revised MS (lines 143-146, 159-168).

Related to this, after the fitting the model fits better, which is logical. One of the major disadvantages of machine learning approaches is that we do not know whether the optimization performed to make the model fit the data, are actually reflecting the optimization that has taken place in nature during evolution, to adjust cellular functioning to temperature, in this case. The authors do carefully analyze the changes that have been introduced during the machine learning optimization, and find that particularly the changes in Topt contribute to the better fit, and that the major change there is the statistical uncertainty, or the variance. I cannot see from the data presented how much these have changed, only the number of proteins for whcih they have changed (Figure 2e). Also, the PCA analysis that appears to describe the fitting trajectory, and the separation of the prior and posterior model fits, has a PC1 whcih only explains 1.15% of the model adjustment, whcih basically implies that there is no way you can truly analyze what has brought about the better fit. I am of the opinion that there should be a validation of the biological relevance of the changes in model parameters, probably tested in vitro. Do the fitted parameters indeed better describe enzyme temperature dependence?

R: As shown in Fig S6, we compare the mean value and variance of the three types of parameters between *Prior* and *Posterior*, which showed how much they have changed. It should be noted that updated parameters in the *Posterior* models still have big uncertainties, as reflected as the *Posterior* variance shown in the Fig S6. An example of an enzyme (GET3) with significantly reduced uncertainties in all three parameters is shown below.

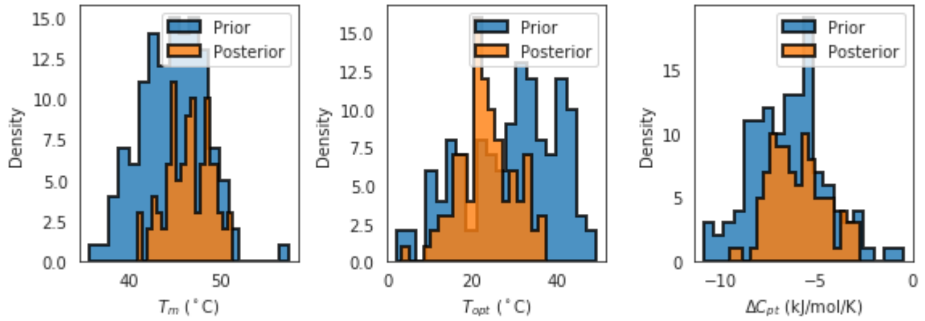

Thereby, direct in vitro validation of those parameters would be challenging since those estimated parameters themselves still do not have a precise value. We thereby firstly compared T_m in the *Posterior* models and experimentally values used in the *Prior* and showed that the *Posterior* mean values are strongly correlated with experimental ones (r=0.97) while the individual ones vary from the mean (Fig 2g). This indicates that the SMC approach does not yield parameters which are very far from experimental measurements to fit the model to the experiment data well. We also compared the T_{opt} values in the *Posterior* models to the experimental data collected from BRENDA. There are 14 such enzymes, and there is a weak correlation (*Pearson*'s r = 0.49) found between the *Posterior* mean value and experimental data. Further comparison was

done by comparing the distribution of *Posterior* mean value of all enzymes and 662 enzyme T_{opt} records for *S. cerevisiae* in BRENDA (only 14 of them can be mapped to specific enzyme in the model based on UniProt ID), we can see that distribution of T_{opt} values in the *Posterior* is more close to the experimental T_{opt} distributions than ones in the *Prior*, indicating that the SMC-ABC approach did improved the estimation of those T_{opt} values.
Lastly, the PCA plot in Fig 2d was not used to analyze which factors contributed to the better fit of the *Posterior* models. It was used to visualize the parameter sets generated by the SMC-ABC approach. It showed a clear trend of how the *Prior* distributions were gradually updated to distinct *Posterior* distributions, despite the first two components explaining less than 2% of the total data variance (Figure 2d). The small variance explained by the first two PCs indicate that those parameter sets are very similar to each other and uniformly distributed in the parameter space.

We updated the Results section accordingly (lines 173-176, 189-200).

The second major comment concerns the following: Each enzyme specific rate was described as a combination of "active fraction" and T dependent kcat. In figure 1a, the term [E]N(T)is also affected by expression levels, and the term kB also depends on the specific isoenzyme performing the reaction. How does the model account for changes in expression levels, affecting reaction capacity without affecting T dependent active fraction, and changes in isoenzymes expressed, which affect both T dependence of kcat (as it is a different enzyme performing the same reaction) and T dependence of the active fraction (as it has a different primary sequence/ stability, etc).

R: In the original study of ecYeast model (Sanchez et al, 2017, PMID: 28779005), which is the starting point of the etcYeast model developed in this manuscript, all the reactions that are catalyzed by isoenzymes are splitted into several different pseudo-reactions, each of which is for one enzyme. For example, for the following reaction

A + B -> C + D

which has two isoenzymes E_1 and E_2. This reaction is represented with two pseudo-reactions in ecModel as follows:

$1/k_{cat,\ 1,j}\ E_1$ + A +B -> C + D
$1/k_{cat,\ 2,j}\ E_2$ + A +B -> C + D

Therefore, the effect of temperature on different isoenzymes can be evaluated separately.

The ecModel does not account for the changes in expression levels. In ecModel, the k_{cat} is used to constrain the maximal flux that one reaction can carry as $v_j \le k_{cat}^{ij} \cdot [\mathrm{E}]_i$, in which $v_j$is the flux through reaction j, $k_{cat}^{ij}$is the turnover number of enzyme $i$catalyzing reaction j, $[E]_i$is the amount of enzyme i. Since the total amount of all enzymes is limited by $\sum_{i=1}^{n}[E]_i \le [E]_t$, the model can optimize the allocation of this total enzyme amount to different reactions to optimize an objective (e.g. maximize specific growth rate). To describe the temperature dependence of enzymes in the ecModel, we need to make k_{cat} temperature-dependent ($k_{cat}(T)$) and also account for the amount of enzymes in the denatured state ($\sum_{i=1}^{n}([E]_{Ni}(T) + [E]_{Ui}(T)) \le [E]_t$). For reactions catalyzed by isoenzymes, the model will use the cheapest enzyme at a specific temperature

considering both k_{cat} and stability. Detailed description of the model can be found in Method M1.

A last major remark concerns the conclusions about the mechanism for the strong reduction of mitochondrial flux at high temperatures, which the authors attribute to a constraint in total protein synthetic capacity. They themselves already observe a strong temperature dependence of several mitochondrial proteins, how are the two quantitatively contributing? Besides, other authors have suggested a strong temperature dependence of the chaperone machinery for protein import into the mitochondria (Moro and Muga, 2006, https://doi.org/10.1016/j.jmb.2006.03.027) and a defect in mitochondrial biosynthesis/repair (Postmus et al., 2011, https://doi.org/10.1099/mic.0.050039-0). How are these observation related/relatable to the mechanisms inferred from the model?

R: To quantify the contributions of total protein constraint and unstable mitochondrial enzymes to the metabolic shift in chemostat at high temperatures, we first simulated the ATP production at different temperatures when the total protein limit in the model is doubled (Fig 4b and Fig S9 for direct comparison). It showed that doubling this protein constraint can delay the shift from around 37 °C to 38 °C and at 38 °C the total ATP produced by mitochondria is increased to 3-fold. Secondly, by stabilizing three unstable mitochondrial enzymes (by increasing the T_m of ATP1, HEM1 and PDB1 to 50 °C), it showed the same shift temperature point (~37°C) while with increased ATP production from mitochondria at temperatures higher than 37°C (2.5 fold at 38 °C, Fig 4c for different fluxes, Fig S9 for direct comparison between different settings). These results support our statement that the metabolic shift happens due to the protein constraint and unstable mitochondrial enzymes would make the mitochondrial pathway more cost-inefficient for ATP production.

Our approach suggests that yeast cannot use mitochondria for ATP production at high temperatures (>37 °C) due to the high cost and unstable enzymes. These two factors were shown to be sufficient to explain the experimental observed loss of mitochondria functions at temperatures above 37°C. However, they may not be the only factors given the evidence from previous studies about the temperature dependence of yeast mitochondria. From the papers suggested by the reviewer, Moro F et al found that a mitochondrial matrix protein Mge1p, which is essential for mitochondrial functions, loses its functions at temperatures higher than 37 °C due to denaturation and dimer dissociation. Postmus J et al found that at 38°C the inner membrane of mitochondria was severely affected by temperature and this may result in impaired function of mitochondria. All these findings indicate that mitochondria are not evolved to be functional at high temperatures.

We revised the Discussion section by addressing that all these and our findings indicate that mitochondria cannot function well at high temperatures (lines 288-289, 296-298, 413-417).

Minor remarks

1. page numbers and line numbers really facilitate the reviewing process

R: We added page numbers and line numbers in the revised MS as suggested.

2. In the first paragraph of the introduction the authors generously distribute term like the most common (environmental parameter), the most abundant, the most sensitive and the largest (effect), where the objectivity can be disputed. I particularly struggled with temperature being the most common parameter affecting cellular (cellular?) evolution. I would assume competition to be the most common, but this is an argument that would take forever, and cannot be objectively substantiated. The same hold for these other "quantitative" statements. Please adjust.

R: We have adjusted the introduction section as suggested.

3. 2nd paragraph of intro, 2nd sentence is incomplete

R: We have completed this sentence (line 56).

4. 4th paragraph, Much data, not multiple data

R: It has been corrected in the revised MS (line 77).

5. page 7, paragraph 3: "Here, we first used....approach" is a sentence that is grammatically incorrect and therefore incomprehensible

R: We have rephrased this sentence (lines 159-162).

6. Figure 2: explain colors in legend, and in panel a rather than d

R: We added the legend in Fig 2a as suggested.

7. Figure title: statistical uncertainties, because true uncertainties haven't been changed (yet).

R: We have replaced the phrase "parameter uncertainties" with "parameter statistical uncertainties" in the revised MS (lines 134 and 202).

8. Figure 3 d-f, use color-blind friendly colors.

R: We have changed it to color-blind friendly colors.

9. Figure 3 caption, last word, the same instead of equal?

R: we have changed "equal" to "the same" as suggested (line 275).

10. The erg1 mutant was transferred/passaged multiple times, but this is not the same as generations (=cell number duplications), so growth from OD0.1 to OD1.8 is >4 generations within one transfer. Please correct in text and figure.

R: we have replaced the word “generations” to “passages” in both text and figure in the revised MS.

11. Figure 5c, why is growth reduced for both strains after the first passaging? Why does WT also recover after 6 transfers?

R: As the seed was cultivated at 30 °C, in the first passage both mutant and wild-type strains may have not adapted to 40 °C and thereby showed a better growth. This phenomenon was also found by a previous study (Caspeta L., et al. *Science.* 2014, PMID:25278608). Caspeta L. et al suggested that after 7-8 days of subcultivation, the yeast strains can reach the steady stage of growth rate. We followed the same protocol to test our strains.

We repeated the experiments for more passages and found that after two passages of adaptation both wild-type and mutant strains showed stable growth across passages and no recovery was found. The Fig 5c was updated with the new results.

12. Figure 5, caption, I presume the cells were grown until glucose/carbon depletion/stationary phase (a discussion by itself) rather than to steady state?

R: We have replaced the description to “The strains were passaged for 7 times (24 hours for each passage) to obtain stable growth at 40 °C”. We didn’t characterize the exact phase of cell growth in each passage while we did passages every 24 hours instead, as the growth phase in each generation could be different, and the time point that wild-type and mutant strain that reach a specific phase can be different (lines 337-338).

Reviewer #3 (Remarks to the Author):
In this article, the authors develop a new methodology to incorporate temperature-dependent enzyme activity into genome-scale metabolic models through a Bayesian estimation technique. In the technique, the authors develop a model of temperature-dependent enzyme activity that integrates with flux balance analysis modeling. This model requires parameters for each enzyme that determine how its activity changes with respect to temperature. The authors propose a technique that uses three experimental measurements for each enzyme: melting temperature, heat capacity change, and optimal temperature. These measurements are used as prior distributions for a Bayesian inference optimization. When these experimental measurements are missing, the authors use mean values. Using a new sequential monte-carlo, approximate Bayesian inference technique, the authors find distributions of temperature-dependent enzyme parameters that are consistent with a dataset of growth rates and ethanol fluxes for yeast. From these 'posterior' distributions, the authors identify ERG1 as a key bottleneck in the thermo-tolerant growth of the wild-type organism. Experimental integration of a thermo-tolerant ERG1 appears to improve the growth rate after several generations of adaptive laboratory evolution vs wild-type.

R: We sincerely thank Reviewer #3 for detailed reading of our manuscript!

Incorporation of thermotolerance and temperature-dependent growth into predictive metabolic models is indeed an important research topic, and the use of Bayesian modeling to reconcile single-enzyme measurements with systems-level growth rates does indeed appear to be a good way of addressing this issue. However, I have some concerns with the way this strategy was implemented in this study. Most concerning is the sequential monte carlo approach employed by the authors to improve the convergence of their approximate bayesian computation (ABC) algorithm. A key modification they have made to the ABC approach is that the prior distributions are 'updated' during each iteration to be closer to the parameter sets that have been shown to match experimental data. While this would indeed result in more parameter sets that match experimental data, it likely reduces the procedure to a simple stochastic optimization approach (i.e., a genetic algorithm or particle swarm), rather than an estimation of a true posterior distribution. More simply, the prior distributions include information on experimental measurements of enzyme temperature dependence -- by abandoning these priors, the authors likely loose the influence of these measurements on their final parameter distributions. As a result, the authors may be overfitting the relatively few growth and ethanol flux measurements they use as evaluation criteria.

R: The main advantage of using ABC approach over other methods like genetic algorithm and particle swarm is that we can quantify and reduce the uncertainties in the model parameters and also quantify the uncertainties in the prediction made by the *Posterior* models. Only a single model would be obtained from a converged Genetic algorithm or particle swarm algorithm, which may be overfitted given that there are more than 2,000 parameters to be estimated from data of aerobic, anaerobic and chemostat cultivations at data at 8, 8, 6 temperature points, respectively. With the ABC approach, a population of different *Posterior* models (100 in our case) can be generated and each of them can accurately describe the experimental data. As reflected by the big variances of those parameters in the *Posterior* models (Fig S7) and some individual T_m and T_{opt}

points shown in Fig 2g and 2h, parameters of those 100 *Posterior* models are very different from each other. There is truly a high risk that each of these *Posterior* models is overfitted. In machine learning, researchers have found that ensemble learning, which ensembles the predictions from many different models, can take advantage of overfitted individual models (Peter Sollich 1996, http://papers.nips.cc/paper/1044-learning-with-ensembles-how-overfitting-can-be-useful.pdf). A similar concept can be applied to the ensemble of 100 *Posterior* models. To support this, we performed train-validation experiments (Fig S3 and Fig S4) and demonstrated that the ensembled predictions from 100 *Posterior* models generalize well on the unseen dataset and are not overfitted.

The comparison between T_m values in the *Posterior* models and experimental values showed that they are very strongly correlated with each other (Fig 2g), indicating that the SMC-ABC approach does not abandon the priors and that the majority of prior knowledge is not lost.

The corresponding results were updated in the revised MS (lines 159-168, 189-200).

Additional comments:
- using experimental error for the standard deviation of priors with missing measurements seems incorrect, typically you use a wide prior to express uncertainty in those measurements.

R: We did not use experimental error while use the standard deviation of experimental T_m distribution (5.9 °C), which is larger than experimental error (~3.4 °C) for missing values.

- for the experimental confirmation, the wild-type doesn't appear to have reached steady-state growth; additional ALD might make the difference statistically insignificant. I'm also confused why the first generation has such high OD compared to the second generation.

R: We repeated the experiments for more passages and found that after two passages of adaptation both wild-type and mutant strains showed stable growth. The Fig 5c was updated with the new results.

As the seed was cultivated at 30 °C, in the first passage both mutant and wild-type strains may have not adapted to 40 °C and thereby showed better growth. This phenomenon was also found by a previous study (Caspeta L., et al. *Science.* 2014, PMID:25278608). Caspeta L. et al suggested that after 7-8 days of subcultivation, the yeast strains can reach the steady stage of growth rate. We followed the same protocol to test our strains.

Minor Comments:
- "Fig SX" in Figure 3 caption is unclear

R: It should be Fig S1 and was fixed in the revised MS (line 263).

- Fig 5 caption: wide-type instead of wild-type

R: The caption was fixed as suggested (line 335).

- how many data points are used during the ABC calculation? This should be spelled out more clearly.

R: We have updated the number of data points in the results section (lines 143-146).

- what's the difference between fig 5C and fig S9?

R: The experiments for Fig 5C were conducted at 40 °C, while ones for Fig S9 (Fig S11 in the revised MS) were conducted at 42 °C, as stated in figures and figure captions.

Reviewer #1 (Remarks to the Author):

The following responses address the authors' responses to comments from both Reviewer #1 and #2. Overall, the authors have adequately addressed these reviewers' points, and their manuscript is clearer and more compelling as a result.

The additional validations and robustness analyses performed by the authors adequately address major comments from both reviewers and increase confidence in model generalizability.

The authors have addressed the comment about cold denaturation adequately, including caveats noted in the text and expanding the range of temperatures covered on the x-axes in Fig 3d-f.

Additional analyses regarding the relationship between kcat, Tm, and specific activity convincingly support that kcat is more limiting in enzyme specific activity than Tm in the superoptimal temperature range of focus for this study.

The authors have better explained the limited nature of available experimentally-determined thermal parameters, which suggest a lack of precision in critical parameters reported in the BRENDA database. Compensating somewhat for the uncertainty associated with these parameters is seen as one of the major strengths of the work the authors present.

The authors investigated possible relationships between thermal parameters and both changes in gene expression and enzymatic reaction fluxes as a function of superoptimal growth temperatures. Although there was no significant relationship found, the authors sufficiently addressed this point as far as currently reasonably possible.

New simulations to decouple the contribution of total protein constraint and key mitochondrial enzyme Tms in leading to metabolic shifts observed with increased growth temperature are at least qualitatively consistent with the authors' initial claims. The simulations of increasingly limiting constraints on ergosterol pathway genes were a nice and intuitive way to find a theoretical threshold at which ERG1 is no longer predicted to be the most growth limiting reaction with elevated temperatures. There is a fairly wide range of specific growth rates in these simulations, but the overall result further supports the authors' hypothesis about ERG1.

The authors have repeated the genetic experiments validating their prediction of ERG1 as a metabolic bottleneck in the 40-42C range. Their new data include an additional passage and appear much cleaner than the data they replaced in the initial submission.

Through their revision and direct responses, the authors have adequately addressed all of the additional more minor points raised by Reviewers #1 and #2, except the following minor typographical errors:
1) There is still a typo "wide-type" in the legends for Figures S11 and S13.
2) The Figure S11 legend still says "40C" when the rest of the legend and figure say "42C". Please accurately and consistently note the relevant temperature.

Roger Larken Chang, Ph.D.
Associate Lab Director
Department of Systems Biology
Harvard Medical School

Reviewer #2: unavailable.

Reviewer #3 (Remarks to the Author):

On the whole, the authors have improved the manuscript considerably in their revision, and I think the resulting paper is a high quality study that more than meets the strict quality standards of Nature Communications. However, I have a few remaining minor concerns that it would be helpful if the authors could clarify their work in a revised version.

In their response letter, the authors mention that older stochastic, population-based optimizers only yield a single, best-performing model. While they were often used to produce a single solution, these optimization approaches maintain a population of candidate parameter sets, and thus the entire solution set could be viewed as an ensemble solution, and resulting parameter confidence intervals an be found. The algorithm on Page 21 in particular reads a lot like a population-based evolutionary strategy; where a population of best-performing solutions are kept from round to round and used to generate new, better-performing solutions. This is not a criticism of the manuscript, but I think some discussion of the prior literature on population-based stochastic optimization, and the relevant strengths, differences and similarities of the proposed method might be appropriate (10.1371/journal.pone.0056310 has some references to these types of studies).

A posterior distribution is not just an ensemble population of parameter sets that fit the data, but rather a distribution formally defined by both the prior and likelihood functions. In approximate Bayesian computation, the true posterior is only exactly recovered when the acceptance criteria approaches zero, and the authors have therefore correctly identified that computational time is often limiting. Indeed, closely approximating the true posterior distributions for large, complex models with any statistical certainty is exceedingly difficult, even with more sophisticated Bayesian techniques that place analytical constraints on the prior and likelihood functions that can be considered. This being said, I think the authors need to be a bit more careful with their terminology. In proposing a new Bayesian inference technique, I would expect to see some guarantee of convergence to the true posterior distribution, (if not a mathematical proof, than at least a demonstration for a relatively small model where posteriors could be found via analytical methods or well-accepted MCMC-type approximations.) I suspect the new method likely does not always converge to the true posterior, and so some discussion of these limitations should be noted.

Minor comments:

Line 190: "experimentally values"

Response to Reviewers (R: refers to our response):

We thank the reviewers for the constructive comments. Our point-by-point replies to each reviewer's comments are detailed below. All text changes were highlighted in red in the revised manuscript.

REVIEWER COMMENTS

Reviewer #1 (Remarks to the Author):

The following responses address the authors' responses to comments from both Reviewer #1 and #2. Overall, the authors have adequately addressed these reviewers' points, and their manuscript is clearer and more compelling as a result.

The additional validations and robustness analyses performed by the authors adequately address major comments from both reviewers and increase confidence in model generalizability.

The authors have addressed the comment about cold denaturation adequately, including caveats noted in the text and expanding the range of temperatures covered on the x-axes in Fig 3d-f.

Additional analyses regarding the relationship between kcat, Tm, and specific activity convincingly support that kcat is more limiting in enzyme specific activity than Tm in the superoptimal temperature range of focus for this study.

The authors have better explained the limited nature of available experimentally-determined thermal parameters, which suggest a lack of precision in critical parameters reported in the BRENDA database. Compensating somewhat for the uncertainty associated with these parameters is seen as one of the major strengths of the work the authors present.

The authors investigated possible relationships between thermal parameters and both changes in gene expression and enzymatic reaction fluxes as a function of superoptimal growth temperatures. Although there was no significant relationship found, the authors sufficiently addressed this point as far as currently reasonably possible.

New simulations to decouple the contribution of total protein constraint and key mitochondrial enzyme Tms in leading to metabolic shifts observed with increased growth temperature are at least qualitatively consistent with the authors' initial claims. The simulations of increasingly limiting constraints on ergosterol pathway genes were a nice and intuitive way to find a theoretical threshold at which ERG1 is no longer predicted to be the most growth limiting reaction with elevated temperatures. There is a fairly wide range of specific growth rates in these simulations, but the overall result further supports the authors' hypothesis about ERG1.

The authors have repeated the genetic experiments validating their prediction of ERG1 as a metabolic bottleneck in the 40-42C range. Their new data include an additional passage and appear much cleaner than the data they replaced in the initial submission.

Through their revision and direct responses, the authors have adequately addressed all of the additional more minor points raised by Reviewers #1 and #2, except the following minor typographical errors:
1) There is still a typo “wide-type” in the legends for Figures S11 and S13.
R: Corrected from “wide-type” to “wild-type” in the legends of both figures.

2) The Figure S11 legend still says “40C” when the rest of the legend and figure say “42C”. Please accurately and consistently note the relevant temperature.

R: Corrected from “40 °C” to “42 °C” in the legend.

Reviewer #2: unavailable.

Reviewer #3 (Remarks to the Author):

On the whole, the authors have improved the manuscript considerably in their revision, and I think the resulting paper is a high quality study that more than meets the strict quality standards of Nature Communications. However, I have a few remaining minor concerns that it would be helpful if the authors could clarify their work in a revised version.

In their response letter, the authors mention that older stochastic, population-based optimizers only yield a single, best-performing model. While they were often used to produce a single solution, these optimization approaches maintain a population of candidate parameter sets, and thus the entire solution set could be viewed as an ensemble solution, and resulting parameter confidence intervals can be found. The algorithm on Page 21 in particular reads a lot like a population-based evolutionary strategy; where a population of best-performing solutions are kept from round to round and used to generate new, better-performing solutions. This is not a criticism of the manuscript, but I think some discussion of the prior literature on population-based stochastic optimization, and the relevant strengths, differences and similarities of the proposed method might be appropriate (10.1371/journal.pone.0056310 has some references to these types of studies). A posterior distribution is not just an ensemble population of parameter sets that fit the data, but rather a distribution formally defined by both the prior and likelihood functions. In approximate Bayesian computation, the true posterior is only exactly recovered when the acceptance criteria approaches zero, and the authors have therefore correctly identified that computational time is often limiting. Indeed, closely approximating the true posterior distributions for large, complex models with any statistical certainty is exceedingly difficult, even with more sophisticated Bayesian techniques that place analytical constraints on the prior and likelihood functions that can be considered. This being said, I think the authors need to be a bit more careful with their terminology. In proposing a new Bayesian inference technique, I would expect to see some guarantee of convergence to the true posterior distribution, (if not a mathematical proof, than at least a demonstration for a relatively small model where posteriors could be found via analytical methods or well-accepted MCMC-type approximations.) I suspect the new method likely does not always converge to the true posterior, and so some discussion of these limitations should be noted.
R: We sincerely thank the reviewer for the insightful evaluation and constructive comments. To evaluate the performance of the SMC-ABC approach proposed in this work, as well as to compare it to other population-based methods, we performed simulations by using several models with known true parameter values, including three linear models ($p<N$; $p>N$ with small p, and $p>N$ with large p), a simple non-linear model and an ODE-based model (Elowitz and Leibler, Nature, 2000. PMID: 10659856) where the parameters in the *Posterior* are correlated. Different fitting algorithms were tested, including least squares fitting, genetic algorithm and classical Sequential Monte Carlo based Approximate Bayesian Computation (Toni et al. J R Soc Interface, 2009. PMID: 19205079), which were compared to our approach. The results showed that

(1) GA, classic SMC-ABC and the one proposed in this work showed similar performance when the model is simple, and the number of parameters is smaller than that of samples ($p<N$);

(2) In the case of $p>N$, The SMC-ABC proposed in this work showed the best performance and can make the model less overfitted by showing a larger feasible parameter space; GA and classic SMC-ABC tend to overfit the model. The classic SMC-ABC fails when the number of parameters is high (~100).
(3) In case of correlated parameters in the true *Posterior*, the SMC-ABC proposed in this work can fail as it ignores the covariance between parameters.

Thereby, in the case of the etcYeast model where there are 2,292 parameters to be estimated, the SMC-ABC proposed in this work is the best option among others tested here.

We added the simulation results and discussion in the Supplementary file and referenced to the Method section in the main text. (Line 521-523)

Minor comments:
Line 190: "experimentally values"
R: Corrected from “experimentally values to “experimental values”. (Line 191)

Reviewer #3 (Remarks to the Author):

The authors have more than adequately addressed my concerns, and I appreciate the inclusion of the additional benchmark results for direct comparison against other literature algorithms. The paper in its current form is much more compelling with this addition.
